# Brace technology thematic series: the 3D Rigo Chêneau-type brace

**DOI:** 10.1186/s13013-017-0114-2

**Published:** 2017-03-16

**Authors:** Manuel Rigo, Mina Jelačić

**Affiliations:** 1 Elena Salvá Institute (Rigo Quera Salvá S.L.P.), VÍa Augusta 185, 08021 Barcelona, Spain; 2 Specijalističa Ordinacija za fizikalnu medicine I rehabiliraciju “Ledja I vrat”, Stojana Protića 48, Belgrade, Republic of Serbia

**Keywords:** Idiopathic scoliosis, Non-operative treatment, Bracing, Rigo-Chêneau brace, Scoliosis classification

## Abstract

**Background:**

Chêneau and Matthias introduced in 1979 a brace concept inspired in casting. The brace was initially named “CTM” from Chêneau-Toulouse-Münster. The name “CTM” is still popular in France but “Chêneau-type brace” is its common name in the rest of the world. Principles to construct this brace were originally based on anatomical descriptions rather than biomechanics, and its standard is poor.

**Methods:**

This paper follows the format of the “Brace technology thematic series.” The Chêneau-type brace has been versioned by many authors. The contribution of the present authors is about to the description of the principles based on biomechanics and a specific classification created to help to standardize the brace design and construction. The classification also correlates with specific exercises (PSSE) according to the Barcelona School, using Schroth principles (BSPTS). This current authors’ version has been named “3D Rigo Chêneau-type brace.” The 3D principles are related to a detorsional mechanism created by forces and counterforces to bring the trunk into the best possible correction: (1) three-point system; (2) regional derotation; (3) sagittal alignment and balance. A custom-made TLS brace (thoracolumbosacral) is built in order to provide highly defined contact areas, which are located, shaped, and oriented in the space to generate the necessary vectors of force to correct in 3D. Expansion areas are also essential for tissue migration, growth, and breathing movements, although body reactions depend basically on how well designed are the contact areas. The brace is open in front and can be considered rigid and dynamic at the same time.

**Results:**

Blueprints for construction of the brace according to the revisited Rigo classification are fully described in this paper.

**Conclusions:**

Different independent teams have published comparable outcomes by using Chêneau-type braces and versions in combination with specific exercises and following a similar scoliosis comprehensive care model. This present version is also supported by scientific results from several independent teams.

## Background

This paper, which is about the author’s custom-made version of the popular Chêneau brace, follows the format for the “*Scoliosis* brace thematic series.”

**Fig. 1:**
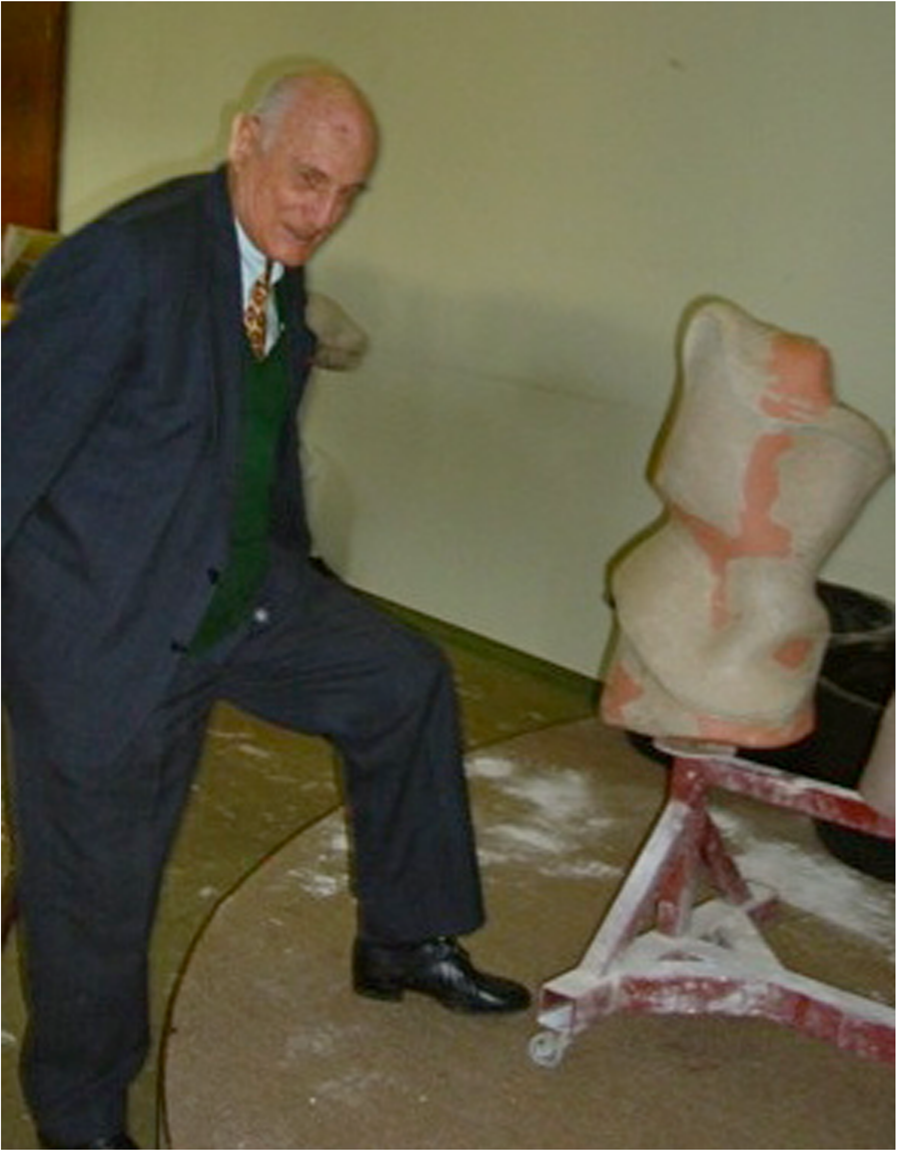
Jacques Chêneau in Bad Sobernheim (Germany) circa 1998. Photo by Sanomed

The effectiveness of bracing in the treatment of adolescent idiopathic scoliosis is no longer a controversial issue. In a systematic review of the effectiveness of bracing treatment, Maruyama et al. [1] concluded that, although the quality of the evidence is limited due to the low methodological quality of the studies, “the available data suggest that, compared with observation, bracing is more potent in preventing the progression of scoliosis and may not have a negative impact on patients’ quality of life.” A previous Cochrane systematic review had showed low-quality evidence in favor of using braces [2]. However, a recent multicenter study about the effects of bracing on adolescents with idiopathic scoliosis, enrolling both a randomized cohort and a preference cohort, concluded that bracing significantly decreased the progression of high-risk curves to the threshold requiring surgery [3]. Corroborating the results of previous prospective observational studies, this study showed a strong brace dose-response relationship, with increased benefits from longer hours of brace wear. However, the statement about the effectiveness of bracing is too general, which raises questions regarding the relevance of such a statement for each individual patient. First, the indications, limitations, and contra-indications are not universally established. Second, the amount of brace concepts with different principles of correction is too extensive to encompass in “a standard.” More so, few principles of correction have achieved the desired consensus among experts [4]. Third, according to SOSORT guidelines (International *Society on Scoliosis Orthopaedic and Rehabilitation Treatment*), independent of the prescribed brace concept, the multidisciplinary treatment team seems to play a contributing role in the success or failure of bracing treatment [5]. Nevertheless, two well-defined factors have been associated with positive results and bracing success: (1) short-term in-brace correction of the Cobb angle and (2) compliance. However, since the reasons behind bracing success are extremely complex, using these two factors as the key points of bracing treatment and scoliosis management is obviously a simplification. In-brace correction depends on several factors, such as the correction principles of the prescribed brace, brace design according to curve pattern, specific quality of design achieved by a particular orthotist, brace fitting, and patient’s characteristics. These combined factors determine how much Cobb angle correction will be achieved; however, ideally, the correction of the Cobb angle should be achieved through a 3-dimensional (3D) correction of the trunk and spine. Historically, the Chêneau-type brace was designed to oppose the spinal torsion and correct scoliosis in three dimensions. The original Chêneau-type brace has been defined and described in several books, primarily published in French and German [6–9] by Jacques Chêneau and his collaborators (Fig. 1), and many European doctors have used the brace since its presentation in 1979. The first author of this paper (MR) initially collaborated with Jacques Chêneau and Hans Rudolf Weiss to develop the technical evolution of the brace and is basically responsible for the re-definition of brace principles using biomechanical descriptions instead of the original anatomical descriptions provided by Jacques Chêneau. The author (MR) is also responsible for the additional brace designs that use a specific classification according to the scoliosis curve pattern [10]. This paper will provide a complete description of the correction principles according to the 3D nature of idiopathic scoliosis. The classification will be revisited with the introduction of some minor changes, and complete descriptions of specific brace designs for each curve pattern will be provided. Finally, indications, limitations, protocols, results, and case reports will be presented according to the recommended format of the brace thematic series introduced by Negrini S and Grivas TB in *Scoliosis* [11].

The original brace, which was presented for the first time by Dr. Jacques Chêneau (Toulouse) and Prof. Matthias (Münster) around 1979, was initially called the Chêneau-Toulouse-Münster (CTM) brace by French physiatrists. The CTM brace was defined as a custom TLSO brace made from a corrected positive mould from a patient’s negative mould. The correction of the positive mould consisted of a complicated process of removing plaster to build a series of pad areas that coincide with prominent regions of the patient’s body in combination with an even more extensive process of adding plaster to build large expansion spaces that coincide with sunken regions of the patient’s body. The pad areas were located, shaped, and oriented to provide a combined deflection-derotation effect, while the expansions had to provide the necessary room for tissue migration, growth, and breathing movements. Chêneau was inspired by Abbot’s plaster cast. Abbot used this same deflection-derotation principle, putting the patient in the best possible corrective position by pushing the humps and decollapsing the sunken regions of the trunk, keeping the correction with a plaster cast that basically contacted the body on the humps.

**Fig. 2:**
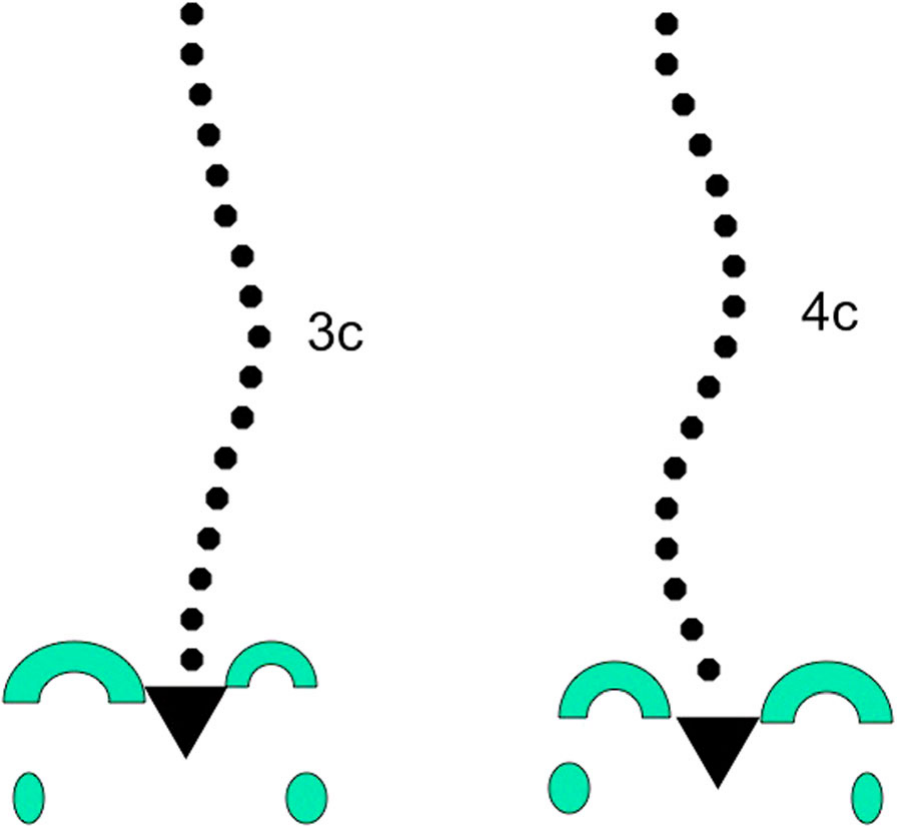
Chêneau classification recognized two types: three curves and four curves, corresponding to simple structural and double structural. This classification was introduced early in the 1990s but considered insufficient later by some expert clinicians

**Fig. 3:**
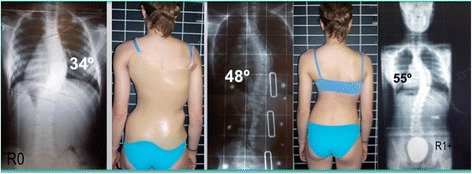
This adolescent was first seen with a right thoracic curve measuring 35°. She was recommend wearing a Chêneau-type brace and received a totally wrong designed brace (deficient four-curve design for her three-curve pattern). The X-ray in brace showed an increased angle of 48° with a change in the curve pattern, adding on the curve some lumbar vertebrae due to the inexplicably strong left thoracolumbar pad. The girl was recommended to continue wearing the wrong brace with no modifications. One year later, a new X-ray out-brace showed a curve progression to 55° Cobb. The persistence of the original curve pattern, demonstrated the improper action of the left thoracolumbar pad, real cause of a temporary in-brace adding on phenomenon

Chêneau made a highly detailed description of the prominent and sunken regions of the scoliotic body in order to explain where to build pads and where to place expansion rooms when performing the correction on the positive mould. All the regions (prominent and sunken) where numbered, forming a numerical map with the purpose of helping the orthotists in their correction task. He also used the concept of “correction of the flat back.” Some French masters used this concept in the 1950s when applying corrective plaster casts. The original mechanism proposed by Chêneau was “to build a strong pad region in the front at the level of the anterior rib hump and the sternum, leaving room on the back to create a kyphosant effect at the thoracic spine region.” Some pictures and descriptions from the abovementioned masters suggest that Chêneau used their concept of overcorrection in the frontal plane for most effectively decollapsing all the concavities. In accordance with the teachings of Christa Lehnert-Schroth, Chêneau also introduced the simple classification of the 3- and 4-curve pattern (Fig. 2) into the field. It is evident that Chêneau was always open to his European colleagues’ opinions and suggestions when improving the brace design. The number of people who contributed to the evolution of the brace is too numerous to list. The benefit of these myriad contributions is that it enhanced the logical evolution of the brace; however, it conversely produced an endless supply of Chêneau-type versions that lacked the high standards associated with the originals. In addition, Chêneau’s classical anatomical descriptions and explanations about where and how to make the corrections to the mould, while easy to interpret by some orthotists, were confusing to most, which has been associated with a very poor bracing standard, resulting in the serious consequence of brace failures and worsened prognosis (Fig. 3). Therefore, we should ask ourselves: What is a Chêneau brace? There is only a possible answer: A Chêneau brace is a brace made by Chêneau himself or made following his direct instructions. Any other brace could be called a “TLSO custom-made brace constructed according to Chêneau’s principles” or, to simplify, a “Chêneau-type brace,” where every prescribing doctor and constructing orthotist has the final responsibility of the brace design and manufacture, the fitting, and, consequently, the end result. In other words, the Chêneau-type brace should not be considered to be simply an “orthopedic product” that can be prescribed by any doctor and built by any orthotist, but a very complex corrective device that must be applied by highly experienced doctors and orthotists. To successfully and safely use this technique, both the MD and orthotist need a relatively long learning curve before reaching the desired standard; therefore, nobody should attempt this technique without being extensively and thoroughly supervised by a recognized master.

The main author (MR) has been intermittently in contact with Jacques Chêneau since 1989 and has been correcting the moulds of patients being treated at the Institut Elena Salvá in Barcelona since 1991. The observed poor standard, with totally different Chêneau-type brace designs for the same curve pattern, was the main impetus behind this author’s proposition of a standardized treatment method in the late 1990s. The standardized treatment method consisted of redefining the theoretical principles, brace construction, and classification. Since 2002, the results of this proposition have been shared with many orthotists, MDs, and PTs during a yearly course offered at the Bundesfaschule für Orthopadie Technik (BUFA) under the name of “Chêneau Korsett nach Rigo,” and have been partially published in two papers [9, 10]. In the next section, the authors describe in detail the theoretical principles of the Chêneau-type brace according to their own interpretation of how the brace should work. Consequently, the following principles would be better called “principles and recommendations from Rigo and Jelačić to construct a Chêneau-type brace or Rigo-Chêneau-type brace.” Classical anatomical descriptions, such as the region map (i.e., pads and expansions) published by Chêneau in the past, shall not be reproduced here. To clarify, some orthotists improperly use the name RSC when building their own Chêneau-type braces following these current principles; it exists in a CAD CAM version—a commercial product with the registered name of Rigo System Chêneau or RSC®—which uses a German company to reproduce braces from a library of original plaster moulds designed by the main author (MR), so the name RSC should not be used by those creating their own custom-made versions of the Rigo-Chêneau-type brace.

## Methods

The 3D TLSO Rigo-Chêneau-type brace is a corrective device uniquely constructed to bring the trunk and spine into the best possible postural and morphological 3D corrected alignment by using a combination of forces applied to the trunk surface by specifically designed pads, facilitated by expansion or escaping spaces. As such, this is not a full-contact or almost-full-contact plastic, anatomic, and symmetric brace with pads inside to push the humps. All the pads are located, shaped, and oriented in a highly specific manner to push on selected regions of the trunk to bring the patient into the best possible 3D correction, while the remaining areas are not touched by the brace (i.e., areas of expansion or escaping spaces). The corrective reaction of the body depends on the level, shape, and orientation of the pads.

The authors have been following the general principle of correction defined in 1992 by Jean Dubousset during his amazing lecture about the importance of the 3D concept in the treatment of scoliotic deformities [12]. Dubousset defined the scoliotic deformity as “a combination of torsional regions joined by junctions; every torsional region formed by a variable number of vertebrae in anatomical lordosis, rotated and translated to the same side.” In the section about practical considerations on cast and brace treatment, Dubousset remarked that “efforts at reducing a scoliotic curve had to be directed toward reduction of the structural lordosis and application of a detorsional force rather than the previous distraction force.” Thus, by applying a detorsional mechanism, the objective is to achieve maximum derotation with the best possible alignment in the frontal as well as the sagittal planes.

The necessary detorsional forces to achieve the desirable 3D correction can be produced with a static brace by combining the following three mechanisms or systems:

1. Three-point systems in the frontal plane
2. Pair-of-force for regional and local derotation
3. Correct balance and physiological alignment in the sagittal plane

It is important to note that these principles do not work in isolation but rather in combination and, consequently, the isolated description of one principle after the other will always be imperfect. However, to maintain a logical format, the principles are explained separately below.

### Three-point systems

**Fig. 4:**
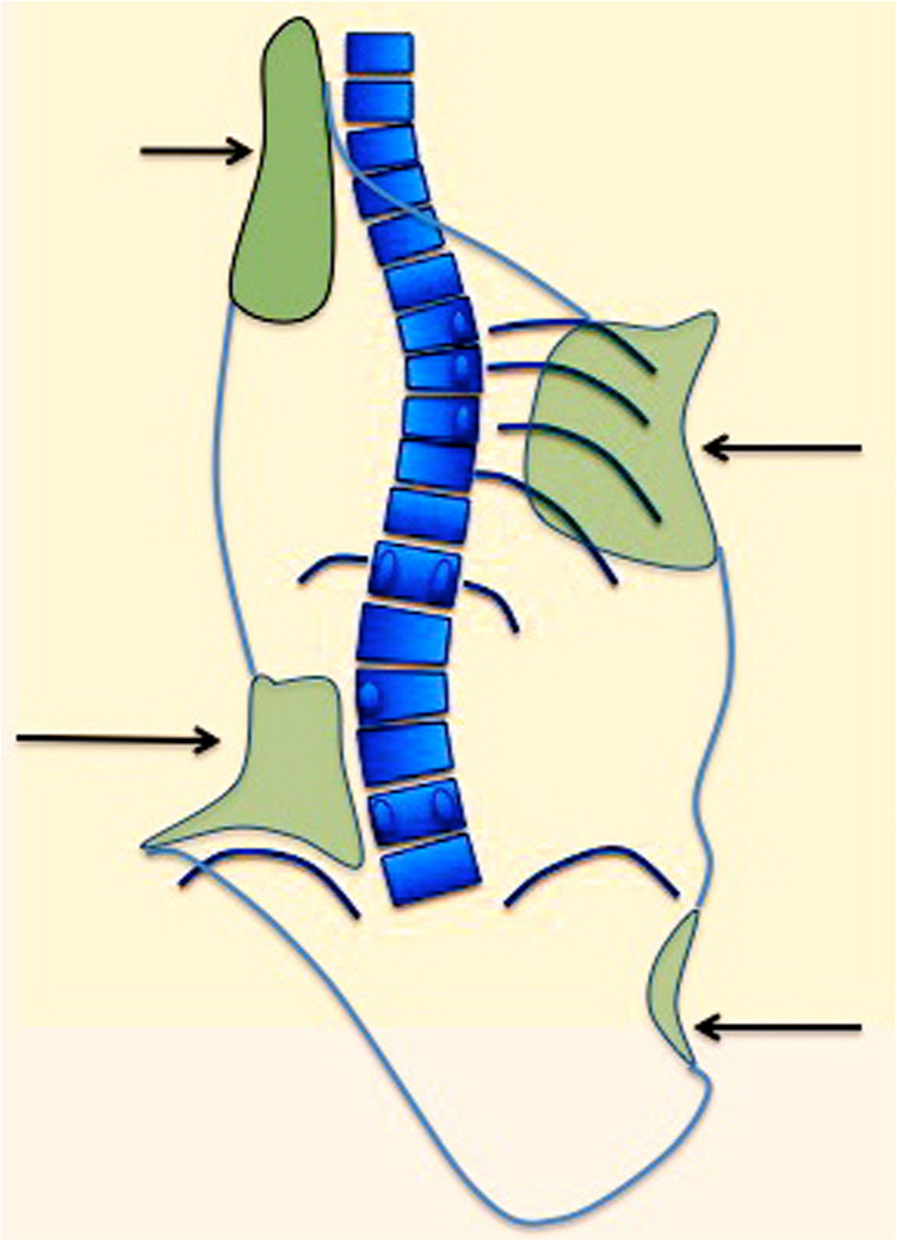
This figure shows the classic principle of the “three-point system,” accepted by most of the specialists treating scoliosis. However, there is no consensus on which level the pads should work to produce the maximum force at the apical region. The corrective force has to be applied on the more prominent regions of the body, but at the same time, the “three-point system” has to bring the trunk into the best possible correction accepted by the postural and soft tissue components

**Fig. 5:**
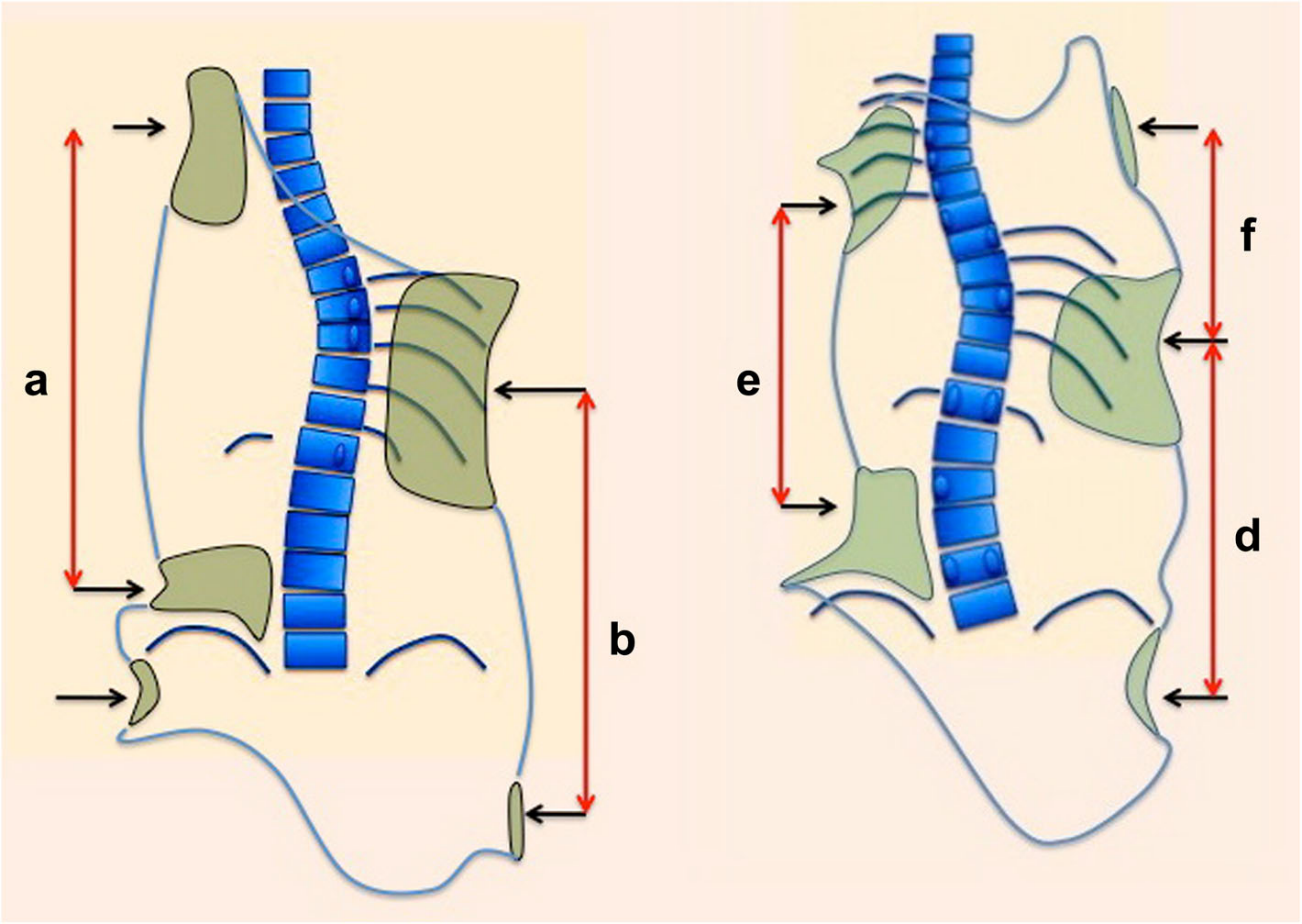
The efficiency of the “three-point system” depends on several factors, among them, the distance between the two proximal and distal points of the system. Curve pattern by itself is one of the factors determining a major or minor correction. Using here the simplest classification of single, double, or triple structural curve, the best correction might be achieved in the simple structural curve pattern and the worse in the triple structural curve pattern. Looking at the main thoracic curve, the distance between proximal and distal pads is higher in the first case (**a**) than in the last case (**e**). The very low distance between the two pads converts the proximal thoracic curve in the most difficult to correct (**f**) in the hypothetical case of locating all the pads underarm

In scoliosis, the lateral curvature of the spine produces a collapse of all the tissues on the concavity, ribs included, when referred to the thoracic region. Alternatively, on the convexity, tissues are expanded. The application of a single three-point system serves to correct single spinal curvature in the frontal plane. The correction of the lateral curvature, which we will refer to as deflection, frees up space on the collapsed concavity and releases tension on the convexity. This correction is essential to allow for derotation. A three-point system is formed by a force and two counterforces applied proximally and distally to the first one (Fig. 4). The direction of the forces and counterforces are always from lateral to medial, but the pads—mainly lumbar and thoracic—providing the vector forces are oriented in an oblique plane rather than in a single frontal plane, so they will also provide the forces for derotation in the transversal plane. The efficiency of the three-point system depends on the level and distance between the three pads designed to create this effect, as well as its orientation in the space (Fig. 5). Thus, the shape and orientation of most of the pads allow them to work as part of a specific three-point system and simultaneously as a pair-of-force system working on the transversal plane to derotate, as explained below. Our speculative theory is that the sum of forces could be producing a detorsional mechanism, to the extent it is associated with an automatic effect of axial elongation, in absence of any traction force. The spatial location (level), distance, shape, and orientation are important not only in both the frontal and transverse planes but also in the sagittal plane to achieve the best sagittal alignment of the trunk and normalization of the sagittal geometry of the spine (in the sense of the physiological profile).

The observed curve pattern determines the specific design of the pads and expansion spaces. Therefore, it is necessary to use a specific and reliable classification to ensure a good standard. This classification has been described in a previous paper [10] and will be revisited later in this section.

### Pair-of-force system

The pair-of-force system consists of two contrary forces in different directions applied on a somewhat wide section of the trunk at the same level in order to derotate the section (i.e., regional derotation). The pair-of-force system has to apply the highest force at the apical level, where the vertebra is more rotated (i.e., local derotation).

**Fig. 6:**
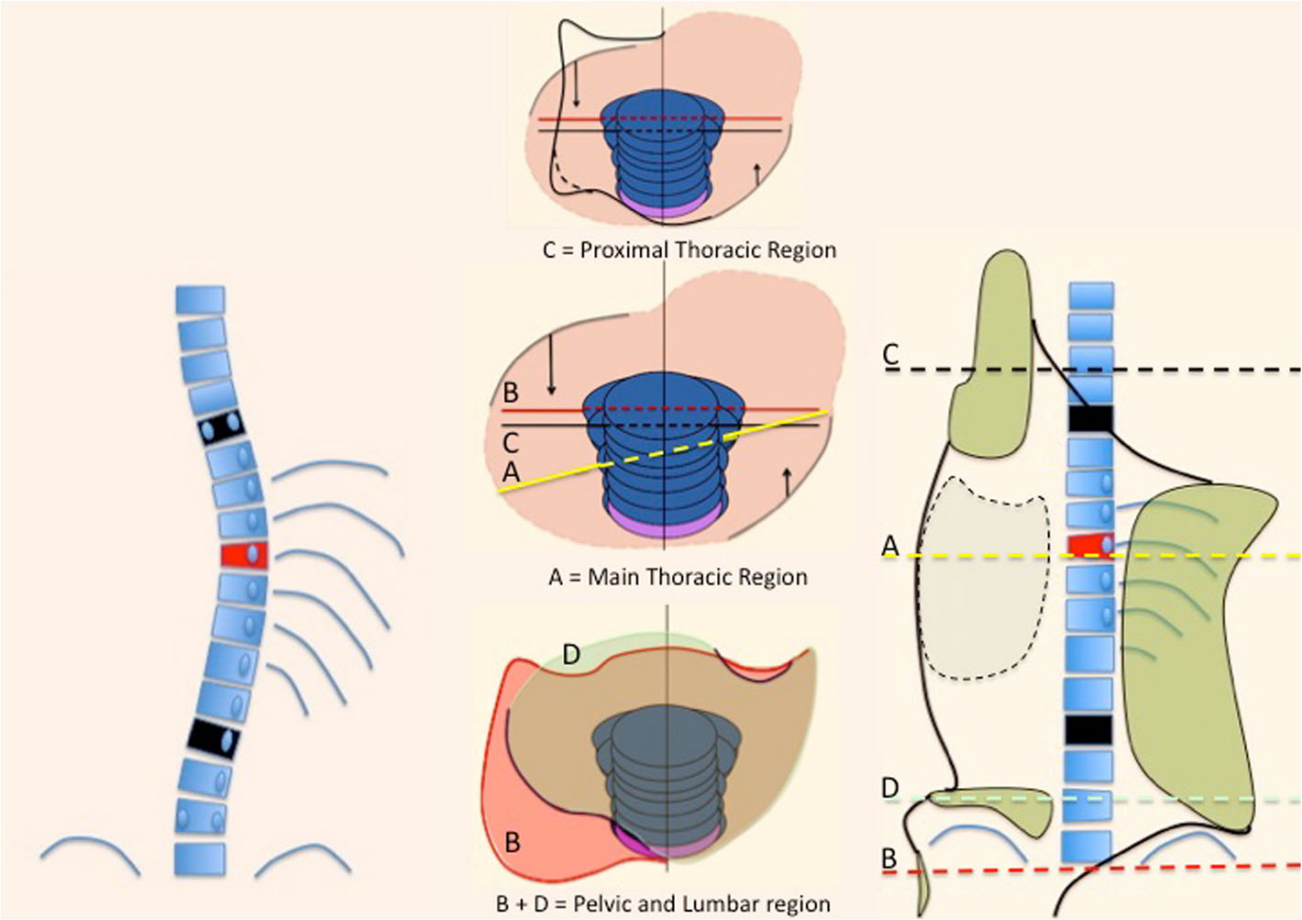
This figure shows the corrective principles for a single long-low thoracic curve with the apical vertebra still in the main-low thoracic region (described later as A1 type in Rigo classification): “regional derotation” and “three-point system.” The region of the trunk affected by the single structural curve is over-derotated to the left (yellow line A) throughout a dorsal-lateral pad and a ventral pad, against the two caudal and cranial regions. Pelvis and lower lumbar regions (B + D) are fixed in the frontal plane of reference (0° of rotation). The pelvis section of the brace is asymmetric, with the lateral-dorsal part opened in the right side and supported by left lumbar contact as well as anterior abdominal contact. The proximal thoracic region (C) is also fixed in the frontal plane of reference with a dorsal left counter-rotation pad. A left lateral to medial pad acts in the proximal thoracic region as the third proximal point of the “three-point system.” The lateral component of the dorsal-lateral pad is the second point, on the right side. The left pelvis section together with the lateral component of the left lumbar support acts as the first caudal point of the system. The brace provides a left lateral-dorsal and a ventral right expansion rooms to facilitate breathing expansion and growth. The dorsal-lateral and anterior pads forming the pair of forces for derotation work both at the same level (maximum force at the apical level). This original design—A1 type—has shown to produce the highest percentage of in-brace correction [64]

To simplify, let us imagine a quite rare case of a right convex single structural curve staring at T5 and finishing at L2 (apex at T9–10), where a relatively wide section of the trunk has to be derotated against the two proximal and distal adjacent regions. In order to get the best correction effect, the proximal and distal adjacent regions can be fixed in the frontal plane of reference (0° of rotation in the transversal plane), while the region affected by the main structural single curve can be over-derotated to the left. Thus, a big region of the trunk, from T5 to L2 is over-derotated against the two adjacent proximal and distal regions, fixed in the frontal plane of reference. The proximal region involves the proximal thoracic region (T1 to T4 in this case) and the distal involves the pelvic and low lumbosacral regions (L4—sacrum and pelvis). The distal section of the brace included a very short low lumbosacral support on the left to ensure that the lowest lumbar vertebrae will remain unrotated, while its immediate proximal region receives an over-derotation force to the left (Fig. 6). This mechanism of regional derotation provides the required detorsional force.

The pair-of-force system also has a special function in the main thoracic region: the correction of structural or anatomical lordosis of the main thoracic curve. This does not refer to the global or regional geometry of the scoliotic spine observed in the lateral projection on the X-ray. The structural or anatomical flat back is related to the torsional phenomena. It has also been defined as relative anterior spinal overgrowth (RASO) [13–23], and although it has been shown to be secondary to the torsion of the spine (Stokes’ vicious cycle modified by Burwell [24, 25]), it could hypothetically be primary [26]. The objective is to achieve the best possible correction of this anatomical lordosis using only the detorsional mechanism while keeping the trunk in a correct sagittal alignment without forward flexion or backward extension. This is the hypothesis of the main author (MR) against the proposal of some colleagues promoting a forced forward flexion applied on the thoracic region in order to correct the flat back. The experience of the author is that, by following this proposal, the anatomical flat back does not attain a better correction, but the proximal and distal regions become more kyphotic. In any case, it must be accepted that, in scoliosis cases with significant potential for progression from a rapid and strong lordotization of the thoracic spine, no matter the design of the brace, the morphological flat back cannot be avoided.

**Fig. 7:**
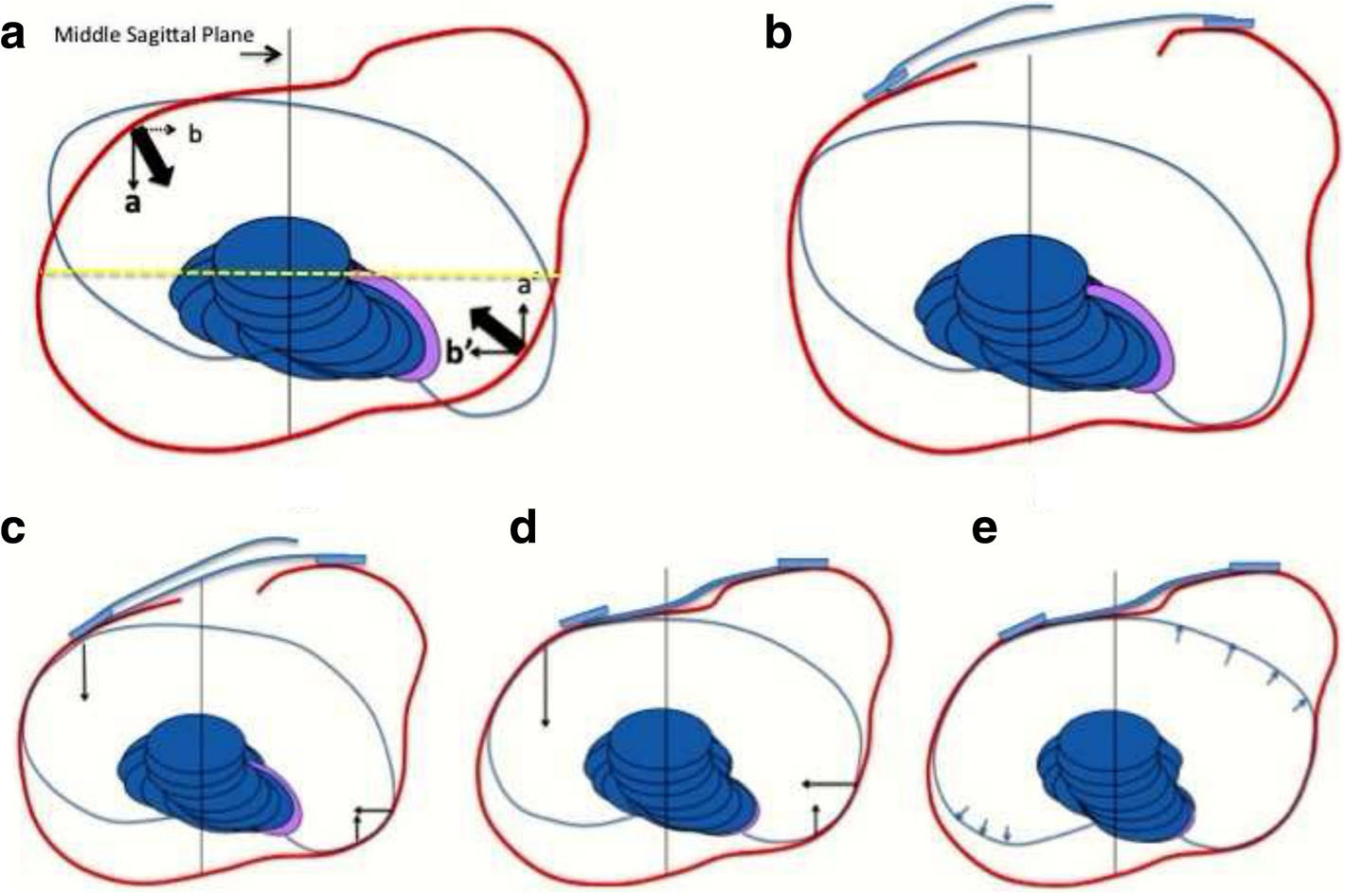
The transversal section of the brace at the main thoracic region is shown in this figure **a**. The sequence **b**, **c**, **d**, and **e** shows the correction induced by the brace, while fitted, at the apical level of the main thoracic region for a right convex scoliosis. The orientation and shape of the pads facilitate local derotation, as shown in figure **a**. The right dorsal pad produces a main vector oriented from dorsal-lateral to ventral-medial direction. This main dorsal vector can be decomposed in two forces, one to medial (*b’*) and the other one to ventral (*a’*). The dorsal pad is closed enough to ventral in its anterior part to reach and still contact the middle axial line, providing lateral support enough. The ventral pad is oriented closest to the frontal plane and produces also a vector, which can be decomposed in two forces, one to dorsal (*a*) and a second to medial (*b*). The section of the brace at the frontal plane passing throughout the “middle axillar line” (*yellow line*) shows how the pad is still contacting the body on the right side but leaving room on the left side. The brace action (biomechanics) is explained by the final forces *a–a’*, forming a pair of forces for derotation, where *a* is a major force than *a’*, and *b’*, which produce the necessary right to left force for translation. While derotating to the left, the apical region will also translate to the left and at the same to the back coupled to the ribs (force *b* will be cancelled by this translation). Breathing mechanics will produce some dynamic reactive forces, increasing the three components *a*, *a’*, and *b’*, with the consequent expansion of non-contact areas. This expansion supposes an additional dynamic effect of derotation, reshaping the thorax, and fighting well against the morphological lordotization of the main thoracic region by increasing the sagittal diameter of the thorax

The proposed principle against the anatomical flat back is not only related to the passive correction but also to a dynamic effect from the breathing mechanics. Figure 7 shows a transversal section of the brace profile at the apical level for a right thoracic scoliosis. The whole section is more or less translated to the left in relationship to the two distal and proximal sections, depending on how translated to the right this region is in the pathological situation. The transversal section shows the two main pads acting at this apical level: the dorsal pad and the ventral pad. It also shows the two main expansion spaces: the ventral expansion space on the right side and the dorso-lateral expansion space on the left side. The shape of this section remains a distorted ellipse rotated to the left (over-correcting the pathological right rotation). The two pads offer two main forces with an oblique direction. The orientation of the pads is always oblique and defines the direction of the forces: (1) the direction of the force coming from the dorsal thoracic pad is from dorso-lateral to ventro-medial and (2) the direction of the force coming from the ventral thoracic pad is from ventro-lateral to dorso-medial. However, the two forces are not in the same direction because the pads are not parallel to each other but are mildly divergent in a dorsal direction. In other words, the ventral pad’s orientation is slightly more frontally oriented than the dorsal pad. The shape of the pads is also an essential point. Both pads are round with a radius that is significantly larger than the radius of the contacted rib humps. Looking at the orientation and shape of the pads it can be noted that the dorsal pad maintains body contact until reaching the middle frontal plane (middle axillar line). On the left side, however, the ventral pad loses contact before reaching this line from the ventral. The main force produced by each pad can be decomposed in two vectors at each contact point and the direction of the vectors is (1) to ventral and to lateral from the dorsal pad and (2) to dorsal and to medial from the ventral pad. Consequently, two vector forces, to ventral and to dorsal, establish the pair-of-force system for derotation, where the vector to dorsal offered by the ventral pad is the major one. On the other hand, the vector force to medial produced by the dorsal pad is more significant than the vector force to medial produced by the ventral pad. Also addressed by the two pads, the whole rib cage section translates to the left, which cancels the vector force to medial created by the ventral pad. The humps adapt to the pads becoming less angular, taking the shape of the pads. This reshaping effect is empowered by breathing mechanics, in inspiration. The ventral flat zone on the right side expands as well as the dorso-lateral concave area on the left. Thorax expansion also creates a dynamic pair-of-force system for derotation. As far as the major vector of force for derotation offered by the ventral pad in a dorsal direction, the apical vertebra, coupled to this force, comes backwards and the sagittal diameter of the thorax increases, with the consequent reduction of the anatomical lordosis or flat back. Each breathing cycle produces a gentle mobilization of the anatomical flat back in the corrective direction. This happens automatically, although the patient can increase this effect by forcing inspiration and trying to keep the two regions expanded during exhalation. Also, maintaining the distance between the sternum and the spine will increase the sagittal diameter of the thorax.

Pelvis section has to be fixed in the frontal plane of reference, with 0° of rotation, or can be mildly over-derotated when it is rotated in the pathological situation. This can be done using a fully closed pelvis section or a partially open pelvis section (i.e., plastic covering one hemi-pelvis). Indications are discussed later in this section (brace design according to curve pattern).

**Fig. 8:**
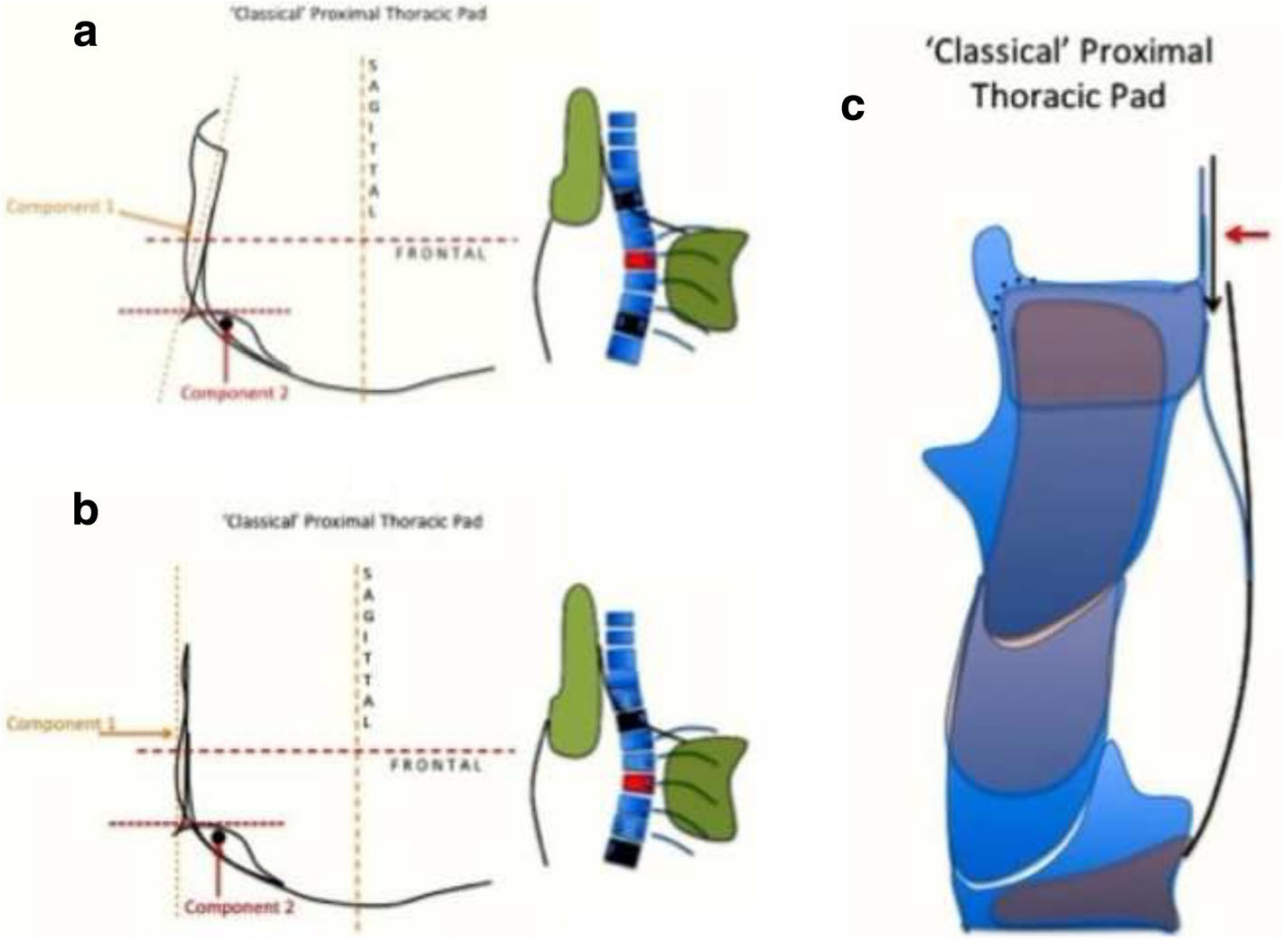
This figure shows the classical proximal pad. It has two differentiated components: component 1 and component 2. Component 1 is the lateral component forming part of the “three-point system” correcting the main thoracic curve. This pad pushes the proximal thoracic region left to right, acting as the proximal point of the “three-point system.” Its orientation depends basically on the observed plane of maximum deformity of the main thoracic curve. It is not so accurate like measuring the angle of the plane of maximum deformity. Scoliosis where the main thoracic curve is more oriented in the frontal plane, component 1 is oriented more in the pure sagittal plane (**b**). Scoliosis where the main thoracic curve is oriented in a more oblique plane to dorsal, component 1 is a little bit closed to ventral (**a**). Component 2 is a counter-rotation pad. Proximal region will tend to rotate to the left when the main thoracic region is over-derotated to the left. This pad stops rotation in the proximal region and help to produce a detorsional effect between the main thoracic curve and the proximal thoracic region. The orientation of this counter-rotation component when observed from the left side is perpendicular to the transversal plane of reference (**c**). The reason for is explained later in the text and Fig. 18. The proximal section is complemented by a ventral pad, which acts preventing the scapular anterior rotation

**Fig. 9:**
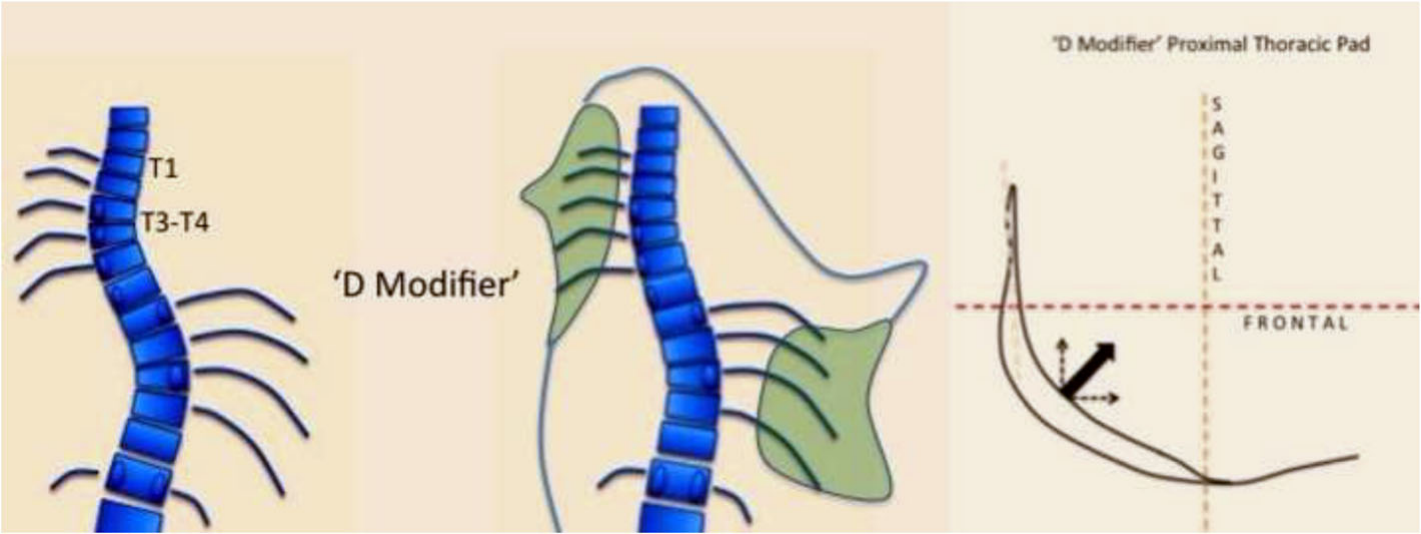
The so-called D modifier is necessary when there is a structural curve in the proximal thoracic region. In such a case, the classical approach has to be modified to this “D modifier” design. The proximal pad has a unique component, like the main pad acting dorsally at the main thoracic region, but its radius is smaller. It does not work so high like the classical proximal pad but still high enough to produce correction of the main thoracic curve as the third proximal point of the “three-point system.” The classical approach is not the best for this case because it will tend to increase the proximal structural curve, if this is already present in a clear way or hidden. The “D modifier” can be complemented by a compressing-traction principle (Fig. 12a)

**Fig. 10:**
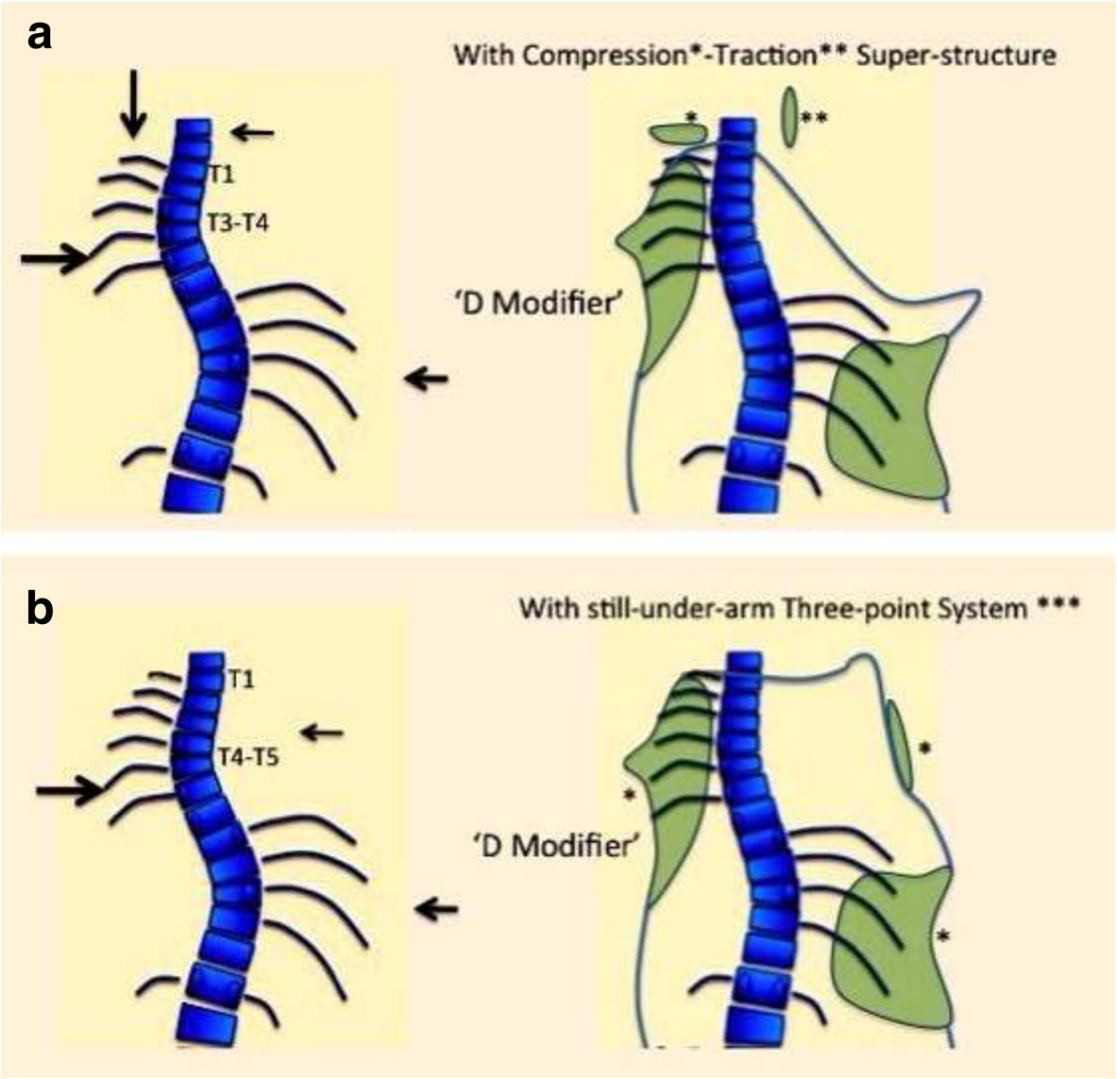
The “D modifier” can be complemented by a compression-traction superstructure, when the apical region of the proximal curve is around T3–4 (**a**). The traction principle, applied from the left side, to pull the neck right to left, is the equivalent to the proximal point of the secondary “three-point system” working to correct the proximal curve, and it is complemented by a compressive force applied on the left trapezium prominent line, classically associated to the structural proximal curve. This proximal point of the “three-point system” can also be provided by an extra-high, but still underarm, right proximal pad, but only when the apical vertebra of the proximal curve is around T4–5 (**b**). Figure 13 shows the prototype of these two approaches

**Fig. 11:**
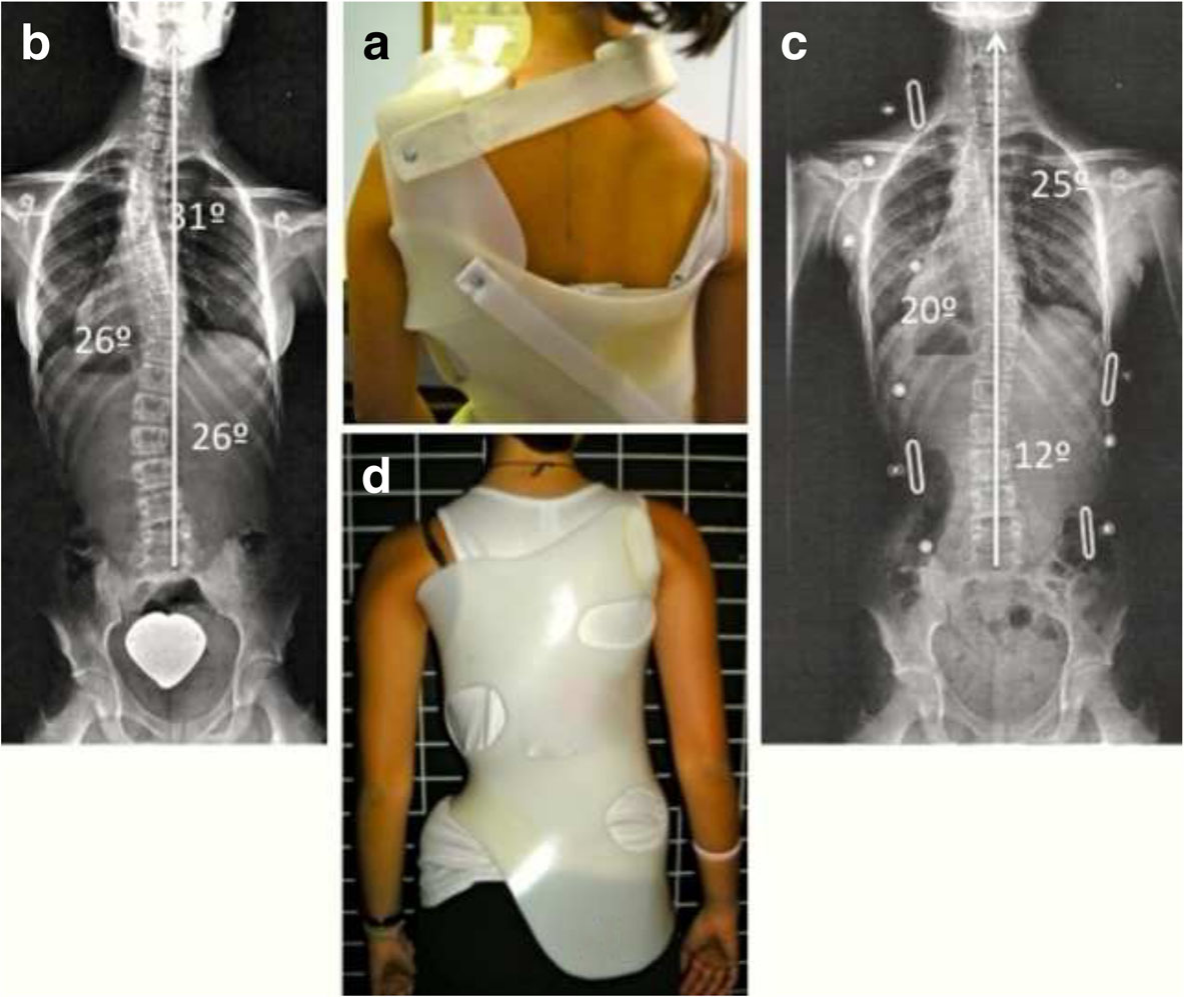
A girl with a primary triple structural curve (left proximal 31°, right main thoracic 26°, and left lumbar 26°) (**b**), was fitted with a first prototype including “D modifier” proximal pad in combination with a removable compression-traction superstructure (**a**). The in-brace X-ray showed a mild correction of all the curves (**c**). Spinal balance was significantly improved with this brace, and proximal curve did not increased but decreased also significantly. After observing that the apical region of the proximal curve was relatively low (disc T4–5), it was decided to design a complex brace, which included a third “three-point system” still working underarm (**d**) (no X-ray available). This exclusive design formed by three underarm “three-point systems” had not been used ever before, to our knowledge, and helped to stabilize this scoliosis. The risk of failure is the highest for this curve pattern, especially in this particular case where the proximal curve is the major and most rigid one

The proximal thoracic region is treated differently depending on the presence of a proximal structural curve or not. If there is no proximal structural curve, the design piece is called “classical.” The classical proximal piece is not a real pad but a combination of two different counter pads. It works at the concave thoracic side and is composed first in a counter pad working oriented more or less on the sagittal plane as a third or proximal point of the three-point system correcting the main thoracic curve in the frontal plane (Fig. 8 explains this in more detail). The second component is a counter-derotation pad, from dorsal to ventral, fixing the proximal thoracic region and shoulder girdle region in the frontal plane of reference (also in Fig. 8). This counter-derotation force is necessary to prevent this region from coming back as a consequence of the over-derotation force applied distally at the main thoracic region. As a counter-derotation pad, this sort of “stopping plate” is oriented on the frontal plane and has to be strictly perpendicular when observed from the lateral view (see next point about sagittal alignment and physiological profile). A second design is used when there is a primary or secondary (from previous brace usage) proximal thoracic structural curve. This second design is called “D modifier” (in accordance with the specific terminology used in the classification), and it is defined as a real proximal thoracic pad, which, like the main thoracic pad, is oriented to ventral and medial, and round shaped with a radius lightly bigger than the one of the ribs forming the proximal dorsal rib hump (Fig. 9 explains this in more detail). Most of the time, the “D modifier” pad needs to work in combination with a removable compression traction or just compression superstructure (Figs. 10 and 11). Please see further explanations in the text (cervico-thoracic region).

### Correct balance and physiological alignment in the sagittal plane

A necessary base for correct trunk balance in the sagittal plane is a neutral pelvis inclination. Pelvis inclination must be in accordance with the individual “pelvic incidence.” A correct sagittal balance depends basically on the relationship between pelvic indexes and the values of the maximum lumbar lordosis and maximum thoracic kyphosis, in absence of any sagittal morphological deformity. Thus, the possibility to achieve a correct sagittal balance and a more or less individualized correct sagittal alignment depends on the amount of morphological lordotization observed in the main thoracic region in relationship with the pelvic incidence. Reaching a correct sagittal balance and alignment is more difficult in cases with higher component of morphological lordotization in any region of the spine.

**Fig. 12:**
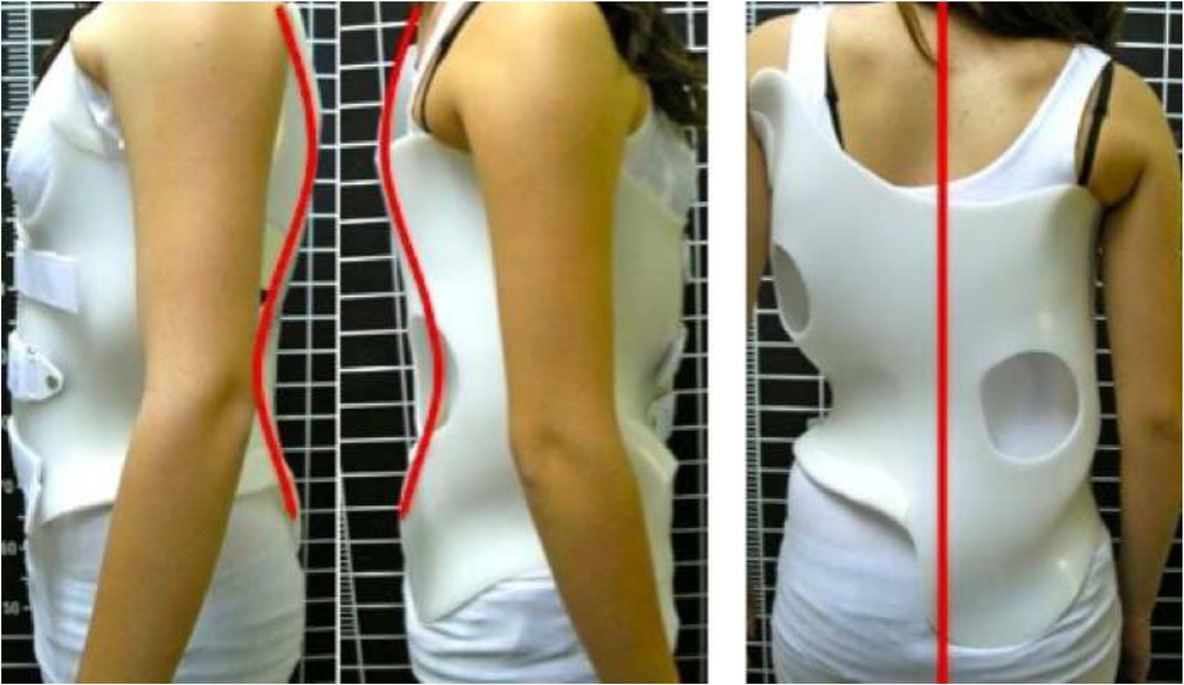
The brace is always built with a more or less physiological profile. However, this cannot be observed from any side because it affects the middle sagittal plane. We currently suggest doing it more physiologic when the subject has a high “pelvic incidence” angle and less (hypo-lordotic/hypo-kyphotic) when the subject has a lower “pelvic incidence” angle. In any case, sagittal postural balance will be the main sign confirming that the designed sagittal profile is accepted or not by any particular patient

Therefore, this brace is not constructed to bring the pelvis into retroversion but supports its normal inclination to provide proximal continuity to the standard lumbar lordosis, all according to three basic sagittal types: (a) normal pelvic incidence, (b) high pelvic incidence, and (c) low pelvic incidence. Neither pelvis retroversion nor lumbar flattening is necessary to achieve good scoliosis correction when the above-explained principles are properly applied. Ventrally, the brace is constructed with expansion enough to produce abdominal contention but not pressure. Unselective abdominal pressure will only produce a flattening of the lumbar spine, opposite to the desired effect. Selective abdominal pressure, on the lumbar concave side, will be used to fix better the brace at this level, helping at the same time to derotate the lumbar scoliotic spine. The physiological sagittal alignment must be observed at the middle sagittal plane of the brace. Since some sections of the trunk are over-derotated, the physiological profile will be hardly recognized when observing the brace from one side or the other. In the classical design of a true double right thoracic/left lumbar curve, the brace will appear hypo-lordotic/hypo-kyphotic (lumbar and thoracic regions, respectively) when observed from the right convex thoracic side, while it will appear hyper-lordotic/hyper-kyphotic when observed from the left concave thoracic side (Fig. 12).

**Fig. 13:**
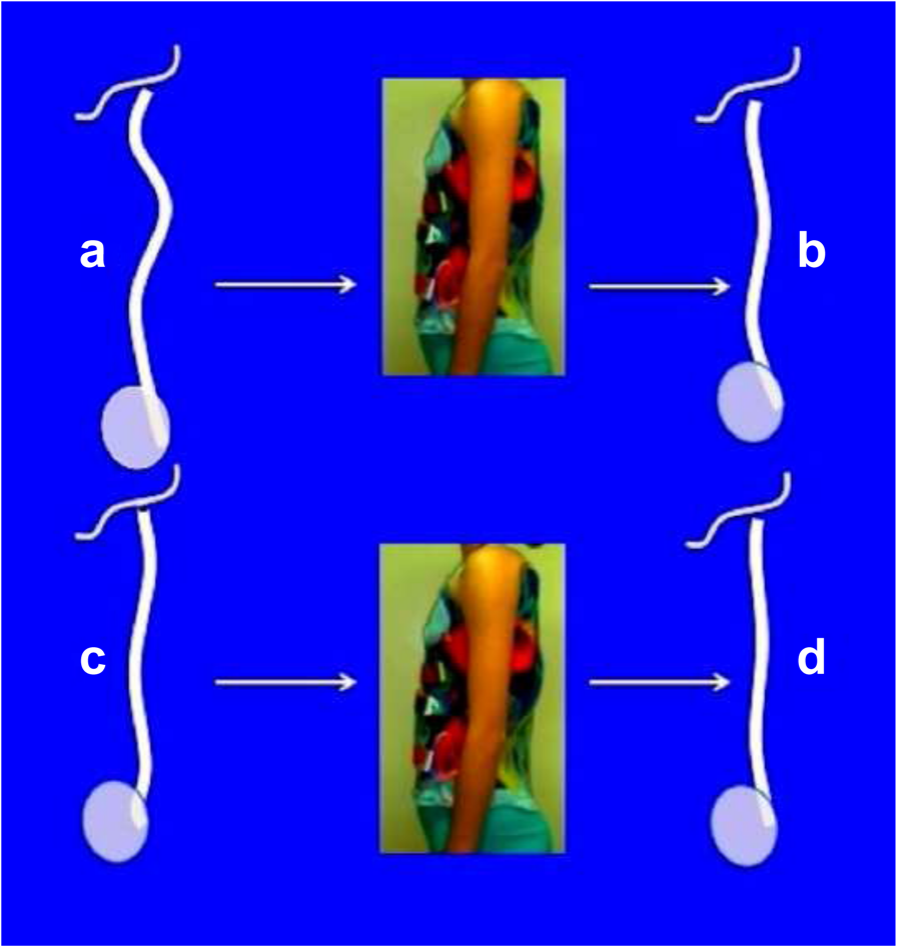
This figure shows the correct sagittal balance and alignment of the brace. No matter which is the observed geometry of the scoliotic spine (**a**, **c**), the brace will have always the tendency to bring it to a more physiologic geometry. However, knowing that the classical thoracic scoliotic deformity has a more or less market structural lordotic component in the main thoracic region we do not expect that the geometry of the spine can be totally normalized by the brace, the same way we do not expect a structural curvature to be reduced until 0°. The sagittal geometry of the spine in brace will be normally hypo-kyphotic/hypo-lordotic (**b**, **d**)

No matter how physiological the brace shape, the sagittal alignment of the spine can hardly be reconstructed as 100% normal unless it is accepted that the correction of the anatomical lordosis in the thoracic region can reach 100% of in-brace correction. Bringing this expectation to the frontal plane component, is it reasonable to expect 100% of in-brace correction in the frontal plane? Why should we expect this in the sagittal plane? In any case, some very flexible spines, particularly those with single, long curves in the thoracic region can achieve total, or almost total, 3D in-brace correction. In most cases, however, the anatomical thoracic lordosis cannot be fully corrected such that the normal anatomical thoracic kyphosis is reconstructed 100%. For example, when considering the Cobb angle in the frontal plane, 50% of in-brace correction would be considered an excellent correction. Thus, a certain flattening of the spine will remain such that the geometry will usually be hypo-kyphotic in the thoracic region and, if well balanced, relatively hypo-lordotic in the lumbar region (Fig. 13).

**Fig. 14:**
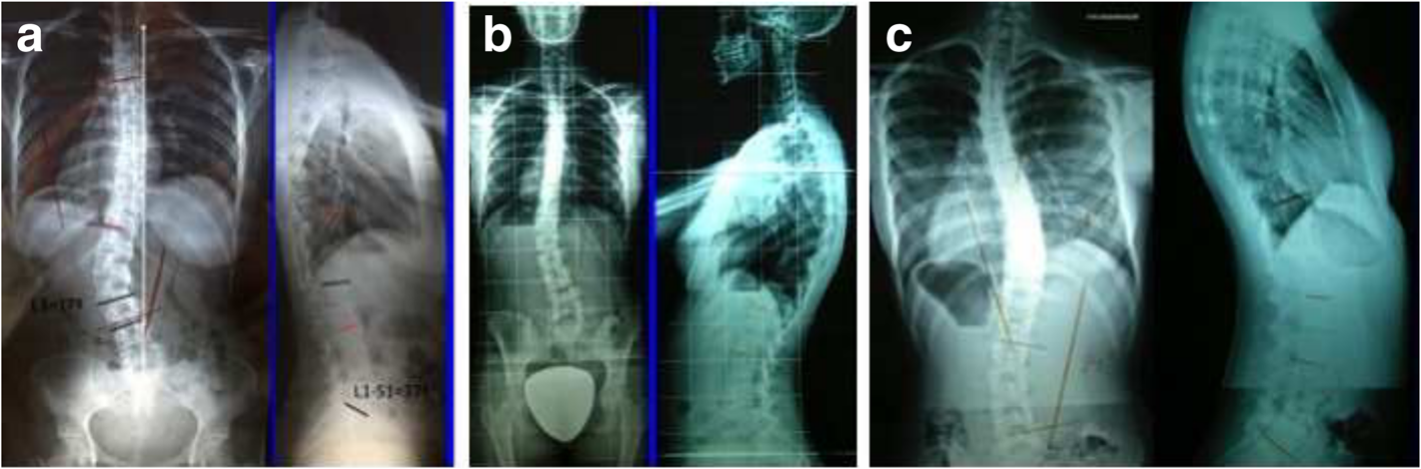
The sagittal configuration of the scoliotic spine is too variable to be simplified with simplistic “dogmas” and solutions, like “scoliosis is a flat back deformity” or “scoliosis comes from **a** kyphotization of the thoracolumbar spine.” The low thoracolumbar curve observed in **a** (apical vertebra L1) presents indeed a thoracolumbar kyphosis in the lateral projection, but the second one (**b**), with much less torsion, shows a still full lordotic configuration in the lateral projection. The very low thoracic curve observed in (**c**) is associated with a proximal thoracic curve and a distal short lumbar curve. The projection of this last scoliotic spine is clearly lordotic at the thoracolumbar region. Junctional thoracolumbar kyphosis is most commonly observed in true double major thoracic/lumbar curves. A torsional phenomenon rather than a single uniplanar failure can explain the high variability of sagittal configurations observed in relationship with the frontal curve pattern

The sagittal geometry of the spine is highly variable [27, 28]. To be pragmatic, scoliosis geometry in the sagittal plane can be described by using general terms: normo-kyphosis and normo-lordosis, hyper-kyphosis and hypo-lordosis, and hypo-kyphosis and hypo-lordosis. Additionally, the point where kyphosis becomes lordosis can be called geometrical transition, and should be located around the anatomical thoracolumbar anatomical region. In scoliotic spines, the geometrical transition can be located more proximally or distally to this region, such that the lumbar lordosis appears to be cranially extended or the thoracic kyphosis appears to be caudally extended. More so, the scoliotic spine can display better or worse harmony in its sagittal geometry; therefore, the regional thoracic and lumbar angles are not enough to describe the sagittal configuration of the scoliotic spine and should not be used to evaluate a brace’s 3D correction. According to Bernhard and Bridwell [29], the anatomical thoracolumbar region (T10 to L2) should also act as the geometrical transition from thoracic kyphosis to lumbar lordosis; it should be neither fully kyphotic nor fully lordotic. This “normal” transitional geometry is observed in many scoliotic spines, while others present “abnormal” fully kyphotic or fully lordotic geometries. Figure 14 describes some examples that support this aforementioned variability in relationship with the curve pattern in the frontal plane.

**Fig. 15:**
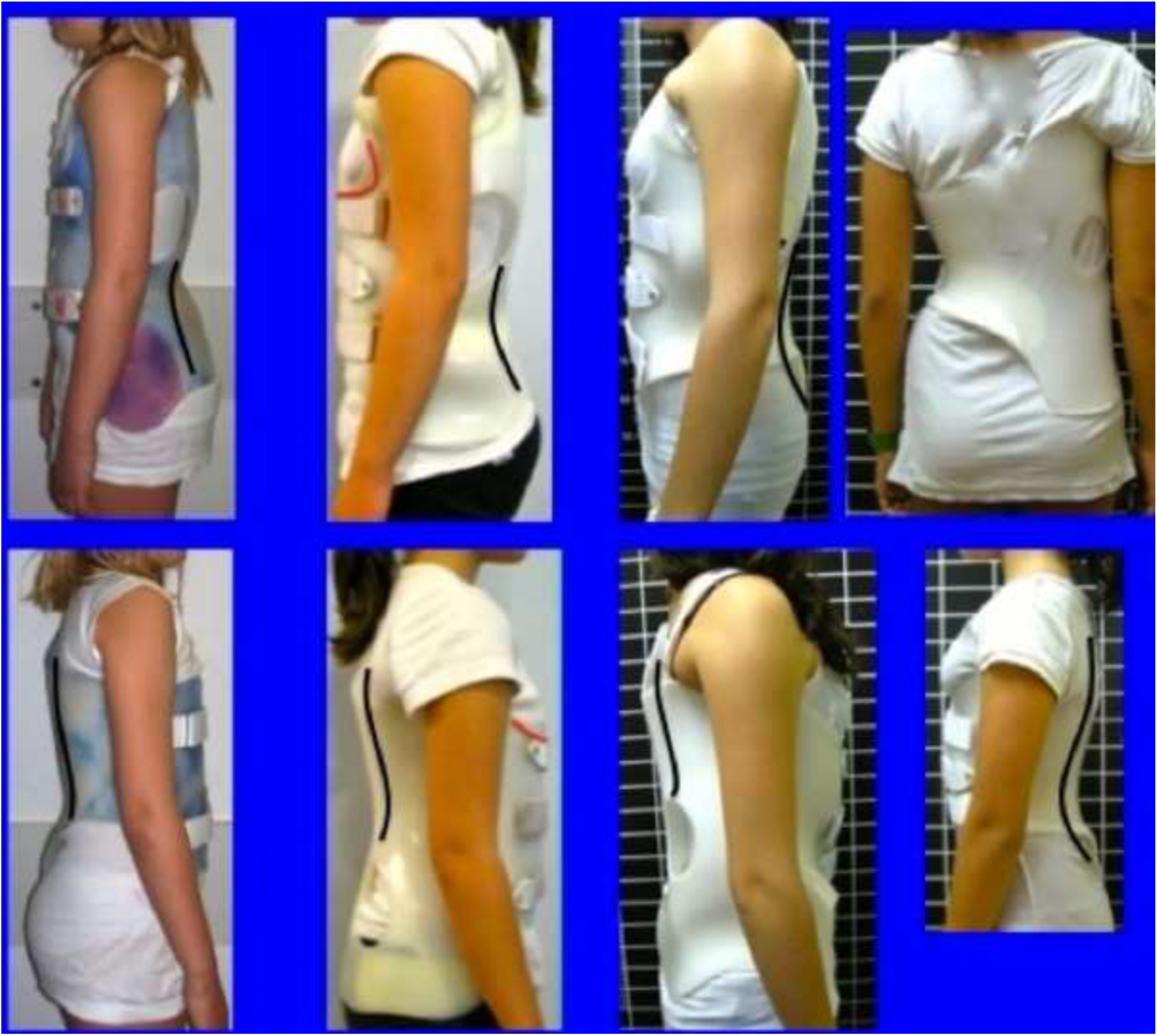
This figure shows four different brace designs. All of them are physiological at the middle sagittal plane but their very market asymmetric design makes them appear very different when observed from one side or the other. First one is an A1 type, second is A2, third is B1, and last E1 (names according to the Rigo classification and brace design)

During treatment, regardless of the curve pattern, a logical objective is to maintain the anatomical thoracolumbar region with its normal transitional kypho-lordotic geometry. As far as the brace using pads on both sides of the spine but not on the spine itself, the best possible restoration of the physiological spinal sagittal profile, including the anatomical thoracolumbar region with its transitional geometry, depends on how the pads from one side or the other are designed from the sagittal view. This method of pad placement is technically complex, as explained in Fig. 15.

**Fig. 16:**
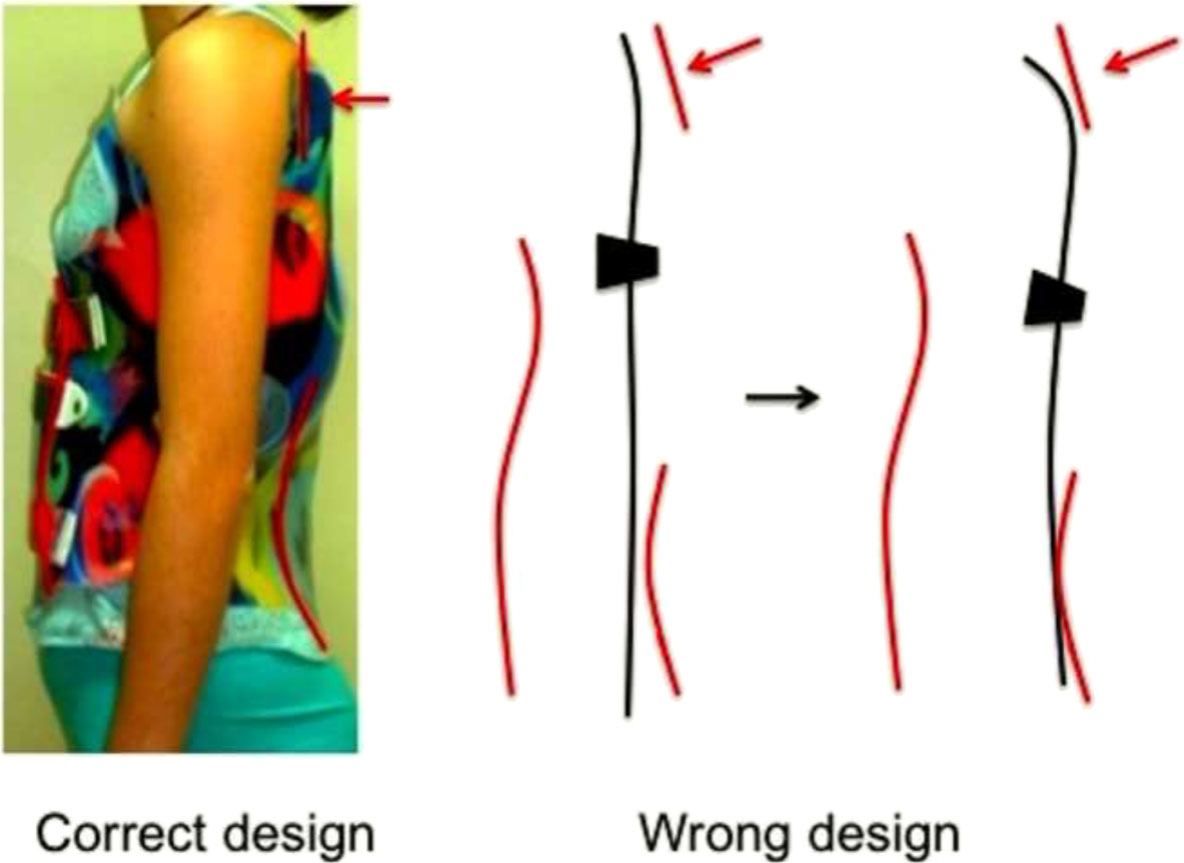
The counter-rotation pad (component 2 of the proximal pad), in all the braces with a classical proximal pad, has to be perpendicular to the transversal plane when observed from the side. The brace is physiological in the middle sagittal plane but on the left side (for right thoracic/left lumbar) the sagittal profile is hyper-lordotic at the lumbar region, hyper-kyphotic at the main thoracic region, and flat and vertical at the proximal, with the counter-rotation pad acting as stopping point. Many orthotists build Chêneau-type brace with this point tilted to ventral, like shown in the figure, but this is a wrong design. When this wrong design is used in a Chêneau-type brace with its classical lumbar lordotic and ventral shapes, the sagittal configuration of the spine shall not be normalized like it is pretended with this proximal pad inclination, but contrary, the main thoracic spine will become even more lordotic and failing in the counter-rotation effect, it will appear a structural proximal curve, which will become rapidly hype-kyphotic. According to our observations, using this wrong design is associated to kyphotization of the proximal thoracic region and the thoracolumbar junction. Inclination of the upper part of the brace, looking for a kyphotization of the main thoracic scoliotic spine, has been used by other concepts, and it could work properly when combined with different forces, but not with the forces provided with a classical Chêneau-type brace

The proximal thoracic region and the cervico-thoracic transition are also considered from the sagittal perspective. The first thoracic vertebrae are still slightly lordotic. The designs of the Chêneau brace concept and some other TLSO concepts, but not all TLSOs, include an extension to the proximal thoracic region. For a classical right thoracic scoliosis, the Chêneau brace is extended cranially, reaching the proximal thoracic region on the left side (i.e., the concave thoracic side in opposition to the convex thoracic side on the right in this example). From one side, this upper extension provides a pure lateral-to-medial force and forms part of the three-point system designed to correct the frontal component of the main thoracic curve (also in Fig. 8). Alternatively, the upper extension also provides a counter-rotation force in a dorsal-to-ventral sense. In the classical Chêneau design, the counter-rotation support is applied on the dorsal aspect of the scapula on the concave thoracic side. From the lateral view, to provide the best counter-rotation force, the orientation of the support must be perpendicular (or parallel to the axial axis). Some orthotists, who were following Chêneau’s principles but were most likely influenced by other concepts, designed this support to be ventrally tilted with the purpose of increasing the thoracic kyphosis (Fig. 16). However, this tilted support, combined with the anterior pad, forces the trunk into a flexion position, which produces an undesirable effect: the support brings the scapula in ventral rotation and does not effectively halt the dorsal rotation of the proximal ribs. This represents a failure of the counter-rotation effect, with the proximal vertebrae rotating in response to the correction exerted on the main thoracic region. Overall, this improper design, rather than creating kyphosis in the main thoracic region, facilitates the creation of a secondary proximal structural curve, which also develops with kyphosis.

The classical upper extension on the concave thoracic side can be generally used unless there is a structural proximal curve, primary or secondary to a previous brace treatment. In that case, we recommend applying a specific design called a “D modifier,” where the upper extension works like a derotational pad rather than a counter-rotational pad. The D modifier is similar to the dorsal pad design for the main thoracic curve. The main limitation to treat a structural proximal curve is the theoretical need of a decollapsing effect on the proximal concavity. This can only be achieved by creating a three-point system with the use of a superstructure. The purpose of the superstructure is to provide a proximal counter-pressure applied on the lateral aspect of the neck on the convex thoracic side (main thoracic curve) while applying a compression mechanism for the proximal convexity on the trapezium prominence. Figure 13 has shown the D modifier working with a type of suggested superstructure, but any other design with similar effect could be used. In any case, the use of the superstructure makes the brace more visible and, thus, increases brace-related stress. We recently introduced a technical variation, which consists of an additional full three-point system, still working underarm, but only in case the apex of the proximal curve is T4–5, not T3. Since adolescents do not readily accept the first technical solution, we previously recommended a removable superstructure to be used only at home, enabling patients to experience a social life without the more visible superstructure. The second technical solution is theoretically more acceptable but, in practice, causes more discomfort due to the relatively short distance between points. Obviously, this curve pattern shows an increased risk of brace failure so clinical control should be very careful in these cases and expectations should be realistic.

## Results

We describe in this section the brace design and blueprints according to curve pattern.

The brace design is based on the application of the aforementioned principles of correction, according to the different curve patterns. A curve pattern-specific classification was developed based on clinical and radiological criteria. The classification contains most of the curve types that require treatment and has been shown to be reliable [10]. In this paper, the classification has been revisited and, after years of use in a clinical setting, some minor changes have been introduced to facilitate its use.

To use the classification properly, we recommend first examining the patient and then reviewing the radiograph.

The first step is to identify one of the following four basic clinical types:

1. Three-curve pattern or A type
2. Four-curve pattern or B type
3. Non-3, non-4 or C type
4. Single lumbar/thoracolumbar or E type

**Fig. 17:**
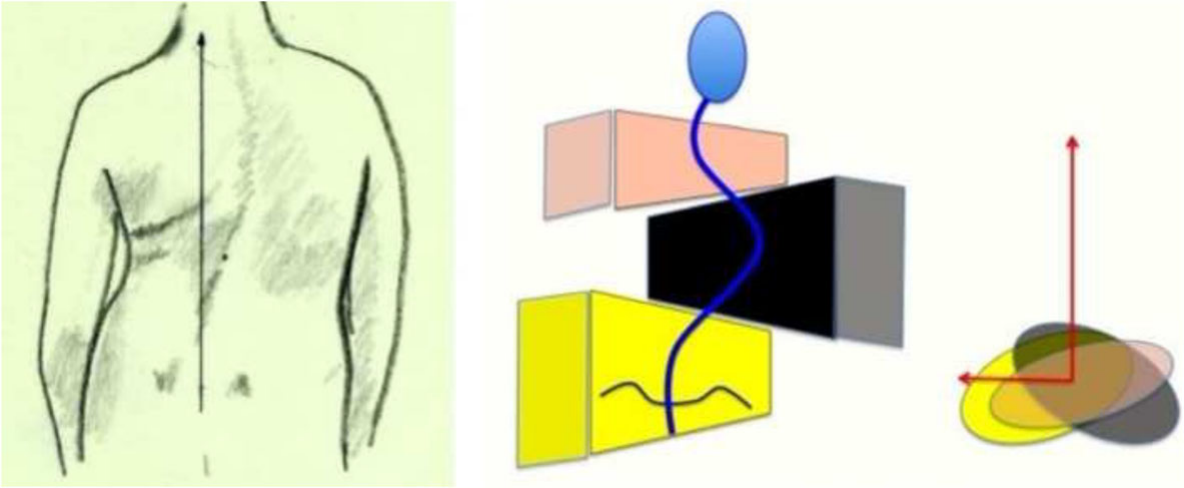
This figure shows the clinical picture and the modified Schroth’s schema of blocks for the functional three-curve pattern (3C). This functional pattern is called in Rigo classification A type. From observation of the clinical picture, the trunk can be here divided into three blocks or regions, with the main thoracic region affected by the main structural thoracic curve and the lower and upper trunk affected by both upper and lower compensations. The three consequent blocks are translated and rotated one against the other, collapsed on the concavities and expanded on the convexities. The main thoracic and proximal thoracic blocks are imbalanced to the right side according to the lower lumbo-pelvic block (including this last the central sacral line). The lumbo-pelvic block is translated to the left according to the polygon of sustentation, with the left hip joint in a relative adduction in comparison with the right hip joint. In case there is a lumbar structural curve, this is still coupled to the pelvis. The schema of blocks offers to the clinicians, physiotherapists, and orthotists a clear composition of the scoliotic phenomenon in 3D and can be taken as a guide for the 3D correction. When the main thoracic curve is convex to the right, the used term is “right 3D or right A type.” The mirror case exists and it is called “left 3C or A type”

**Fig. 18:**
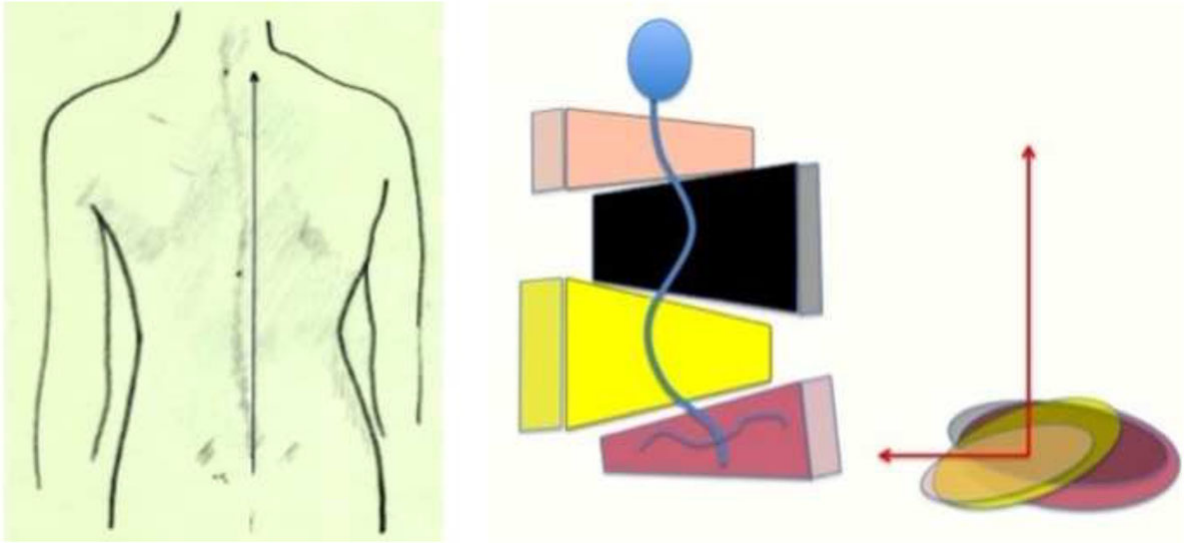
This figure shows the clinical picture and schema of blocks or regions for a four-curve pattern. This is called B type in Rigo classification. This type is characterized by a lumbosacral compensatory curve. The trunk is consequently divided into four blocks or regions, translated and rotated one against the other, collapsed on the concavities and expanded on the convexities. The three upper blocks, lumbar or thoracolumbar, main thoracic and proximal thoracic are imbalanced to the left according to the most caudal pelvic block (including this last the central sacral line). Pelvis is translated to the right according to the polygon of sustentation, so right hip joint is in relative adduction in comparison with left hip joint. This description corresponds to a “right 4C or B type.” The mirror case exists for a left convex thoracic curve combined with right lumbar or thoracolumbar and it is called “left 4C or B type”

**Fig. 19:**
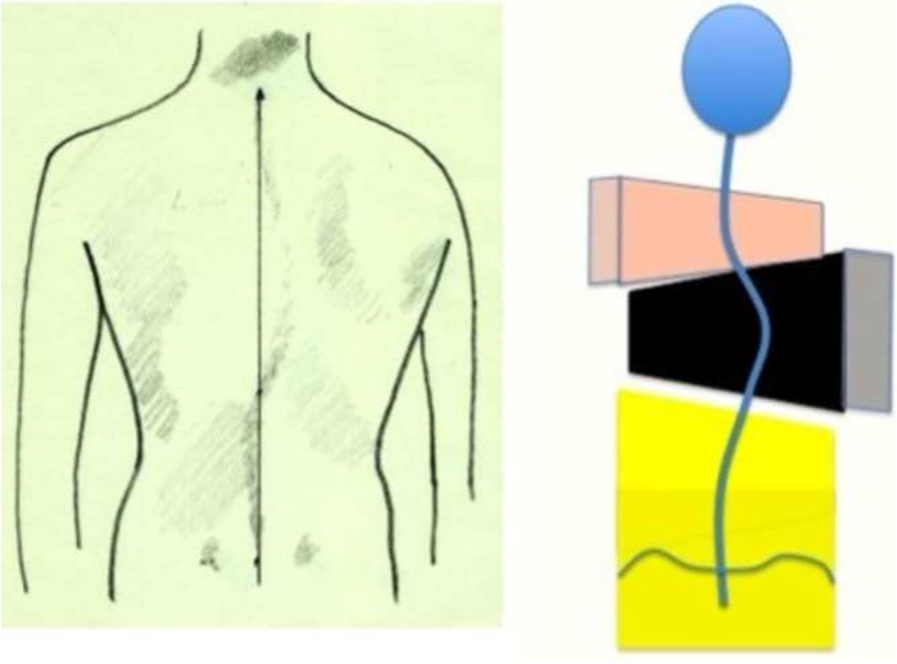
This figure shows the clinical picture and schema of blocks for the so-called non 3-non 4 curve pattern. This is called C type in Rigo classification. The trunk is also here divided into three blocks, like in A type, but there is a minimum translation and rotation of the main thoracic block against the two compensatory lower and upper bocks, which are both well-balanced on the polygon of sustentation. The main thoracic block is collapsed on the concavity and expanded on the convexity. The lower block can be also mildly collapsed on the concavity when there is a structural lumbar curve. The description on the figure is about thoracic curve convex to the side and is called “Right N3–N4 or C type.” The mirror case exists and it is called “left N3–N4 or C type”

**Fig. 20:**
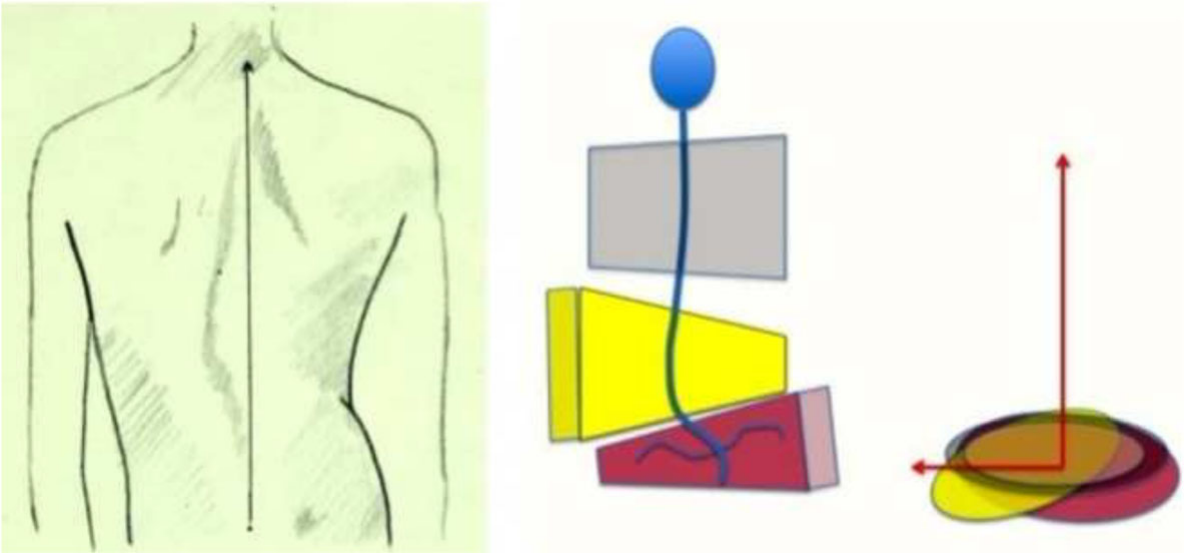
This figure shows the clinical picture and schema of blocks for the functional type defined by a single structural lumbar or thoracolumbar curve. This is called E type in Rigo classification. The trunk is also here divided into three blocks, but the term “3C” here is not a proper one according to the adopted terminology. In fact, looking at the lumbo-pelvic region, E type correlate better with “4C.” Pelvis and lumbar or thoracolumbar region are uncoupled, like in “B type.” Proximal to the lumbar or thoracolumbar curve, there is a unique block more or less symmetric. Trunk imbalance is to the side of the lumbar or thoracolumbar convexity and pelvis is prominent on the concave lumbar or thoracolumbar side. The two lower blocks are translated and rotated one against the other, collapsing in both concavities

This first clinical diagnosis is based exclusively on clinical observations of the patient, without any information from the radiograph. Christa Lehnert-Schroth was the first to describe these basic clinical types [30], whose descriptions herein are slightly different than Lehnert-Schroth’s descriptions. The four basic types, showed in Figs. 17, 18, 19, and 20, have been previously described in the original paper about the specific classification [10].

Also radiological criteria have been previously described [10]. This is a short review, introducing few little changes.

Three radiological criteria are used:

1. Curve pattern compatibility
2. Transitional point offset
3. L4–L5 counter-tilting

1. First radiological criterion: curve pattern compatibility

The curve pattern is defined according to a modified Moe and Kettleson classification [31]. Following is the SRS terminology that is used to define the name of the curve [32].

**Fig. 21:**
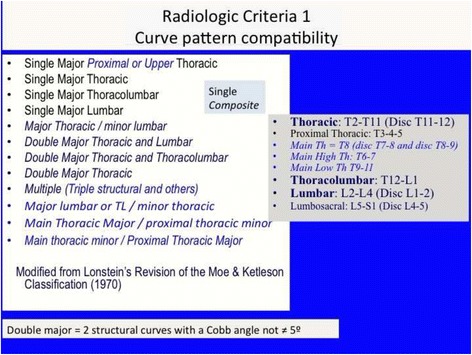
The first radiological criterion is called “curve pattern compatibility.” Any curve is defined according to the apical level following SRS terminology. Structural curve is not defined directly from the radiograph but from clinical observation and exploration. A clinically defined structural curve is used to be confirmed on the radiograph by certain amount of rotation or vertebral wedging (no matter the Cobb angle). Once the curve/s have been defined, we use a modified Lonstein’s revision of the classical Moe and Kettleson classification. Double major is defined when two structural curves have a Cobb angle not different to 5°. Single curve is used just when there is one single structural curve. One pattern more is defined in the composite group, called “major lumbar or thoracolumbar with minor thoracic.” This is here necessary because a real single lumbar or thoracolumbar is classified as E type and will get a short brace while “major lumbar or thoracolumbar with minor thoracic” is classified B type and will get a long brace. The term structural proximal curve is not only used for thoracic double major curve. A minor structural proximal curve can be observed, primary or secondary to bracing. Sometimes the proximal curve is clearly visible clinically but not easy to confirm radiologically (hidden proximal curve). Clinical signs for a proximal thoracic curve are elevation of the shoulder with a prominence of the trapezium line in combination with a deviation of the spinous processes line and costal prominence in forward bending. The proximal curve can be also a major, combined with a minor structural curve in the main thoracic region

Figure 21 summarizes the initial radiological criteria, which is used as a first step in the confirmation protocol once a clinical diagnosis of first suspicion is made. Curve pattern compatibility means that not all curve patterns fit in a particular basic type. As described below, every basic type finds some curve patterns that fit with it and, at the same time, defines a subtype: A1, A2, A3, B1, B2, C1, C2, E1, E2.

Two relevant changes from the original description:

Subtype A1 is characterized by a long-low single thoracic curve. Low means that apical vertebra use to be in the low thoracic region (T9, T10, T11). Long means that the curve goes down into the lumbar region, being L4 the first horizontal vertebra (sometimes L5). If L3 is horizontal, we classify then A2, no matter whether the curve is long and low. In B type, there are always two structural curves, one in the main thoracic region and another in the lumbar or thoracolumbar region. B type is typically a double major curve or a double major/minor, with the lumbar or thoracolumbar curve being the major one. However, it can be also major thoracic and minor lumbar or thoracolumbar. The subtype B1 is defined by the apical vertebra of the lower structural curve at L2 or L1. The subtype B2 is defined by the apical vertebra at T12, the same for subtypes E1 and E2. E1 is like B1 without structural curve at the main thoracic region. E2 is like B2 lacking structural curve at the main thoracic region.

2) Second radiological criterion: transitional point (TP) according to the central sacral line (CSL)

Transitional point was defined in the original paper on classification [10]. We do not use more T1 offset but just transitional point offset to confirm A, B, or C type. The reference is the central sacral line (CSL). Transitional point offset is to the convex thoracic side in A types and to the concave thoracic side in B types. In C type, transitional point is more or less balanced on the CSL. We have not been able to establish a threshold offset value to confirm A or B type at this present time, but we are working on this. It is not easy to differentiate between A and C types in some cases, when TP is not perfectly balanced on the CSL but the offset is not enough to produce a clinical picture where thorax-pelvis imbalance is so clear.

3) Third radiological criterion: L4–L5 counter-tilting

This criterion was also described in the original paper on classification [10]. It is positive when L4 is more tilted than L5 and negative when L4 and L5 are parallel. This criterion is only necessary to confirm B type and, when necessary, to differentiate between B type and C type. B types are associated with a positive L4–L5 counter-tilting. C types are associated with a negative L4–L5 counter-tilting.

E types are like B when describing the lumbosacral region, so it will always show a positive L4–L5 counter-tilting (at least in idiopathic scoliosis).

### The “D modifier.”

Any of the above described A, B, or C type could be associated with a primary or secondary (from previous bracing) proximal structural curve.

A full description of all the radiological criteria confirming the different subtypes A1, A2, A3, B1, B2, C1, C2, E1, and E2 can be seen in Figs. 22, 23, 24, 25, 26, 27, 28, 29, 30, 31, 32, 33, 34, 35, 36, and 37.

**Fig. 22:**
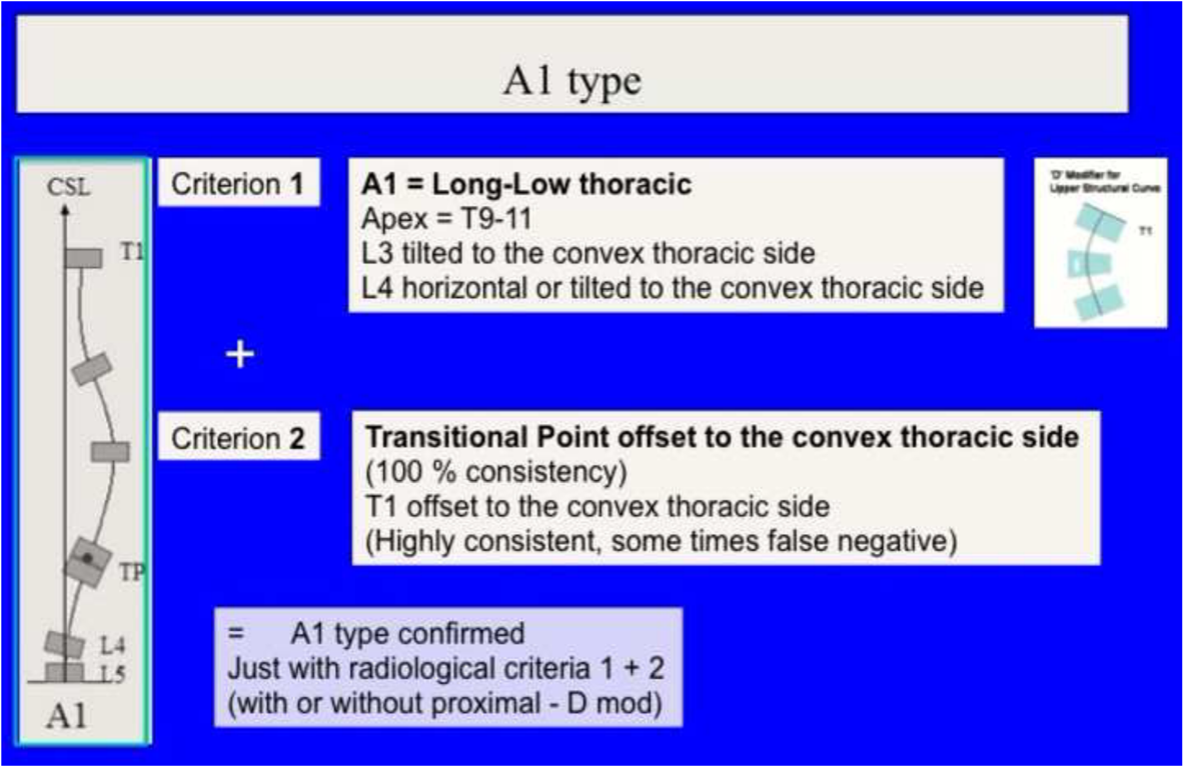
One of the 3C compatible curve patterns is the single main thoracic curve going down into the lumbar region. We can describe this as a “long-low main thoracic curve,” confirming that the apex is still in the main thoracic region, and we call it A1 type. The second radiological criterion is the imbalance of the “transitional point” to the convex thoracic side according to the central sacral line. The transitional point (TP) corresponds to the middle point between the lower end vertebra of the main thoracic curve and the upper end vertebra of the caudal curve, in this case just a functional lumbar counter-curve, which has the real apex at S1. Thus, for this particular curve pattern, the TP is in the middle of the lower end vertebra of the main thoracic curve. To be A1 type, L5 has to be horizontal and neutral; L4 can be already tilted to the convex thoracic side and could be also mildly rotated to that side, but most frequently is horizontal and neutral as well; L3 must be already tilted to the convex thoracic side, and usually with a mild rotation to the same side. It is better not to classify A1 when L3 is horizontal and neutral, even with the main thoracic curve being long and with a low apex. The lower end vertebra is used to be L1 or L2, so L3 could be considered a vertebra of the upper part of a lumbar curve but it is not, because it is rotated to the convex thoracic side, being L4 or sometimes L5 the first “neutral” vertebra. It can be and it is often combined with a structural proximal thoracic curve (D modifier)

**Fig. 23:**
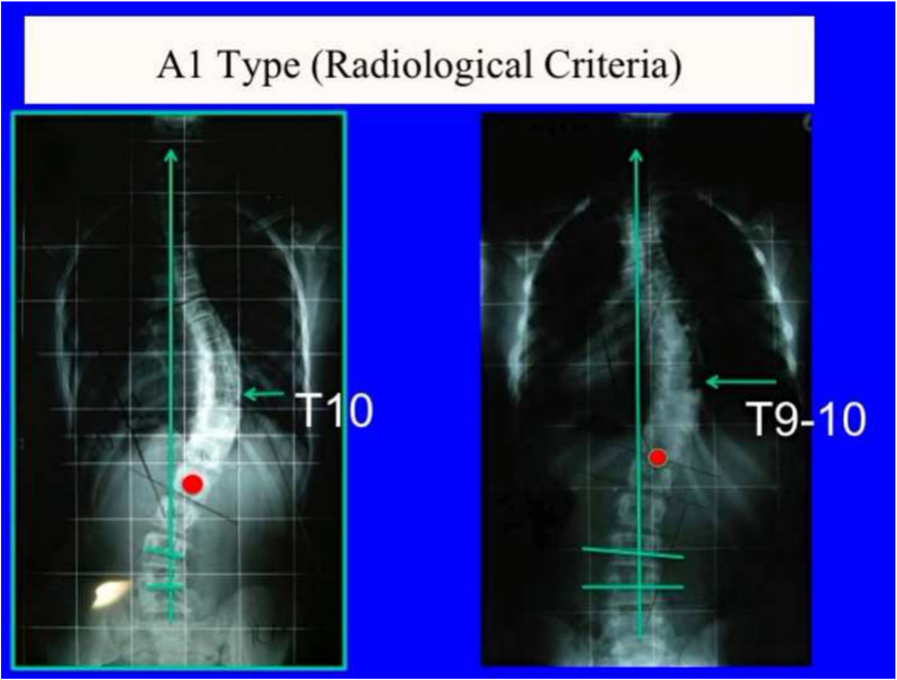
This figure shows two cases of A1 type. Radiological criteria for diagnosis can be found in Fig. 22

**Fig. 24:**
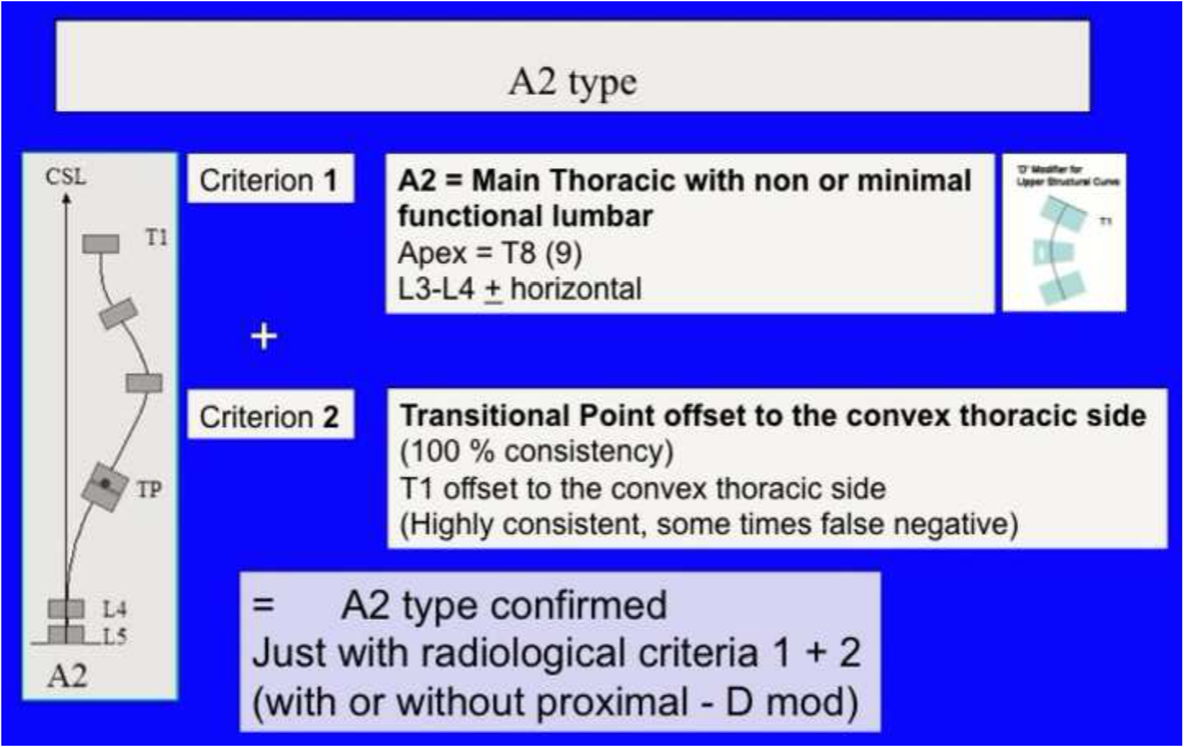
The second 3C compatible curve pattern is the classical “single main thoracic curve” with no lumbar or just a mild totally functional lumbar curve. This is called A2 type. Second criterion for diagnose is the TP offset to the convex thoracic side. It can be combined with a structural proximal thoracic curve (D modifier)

**Fig. 25:**
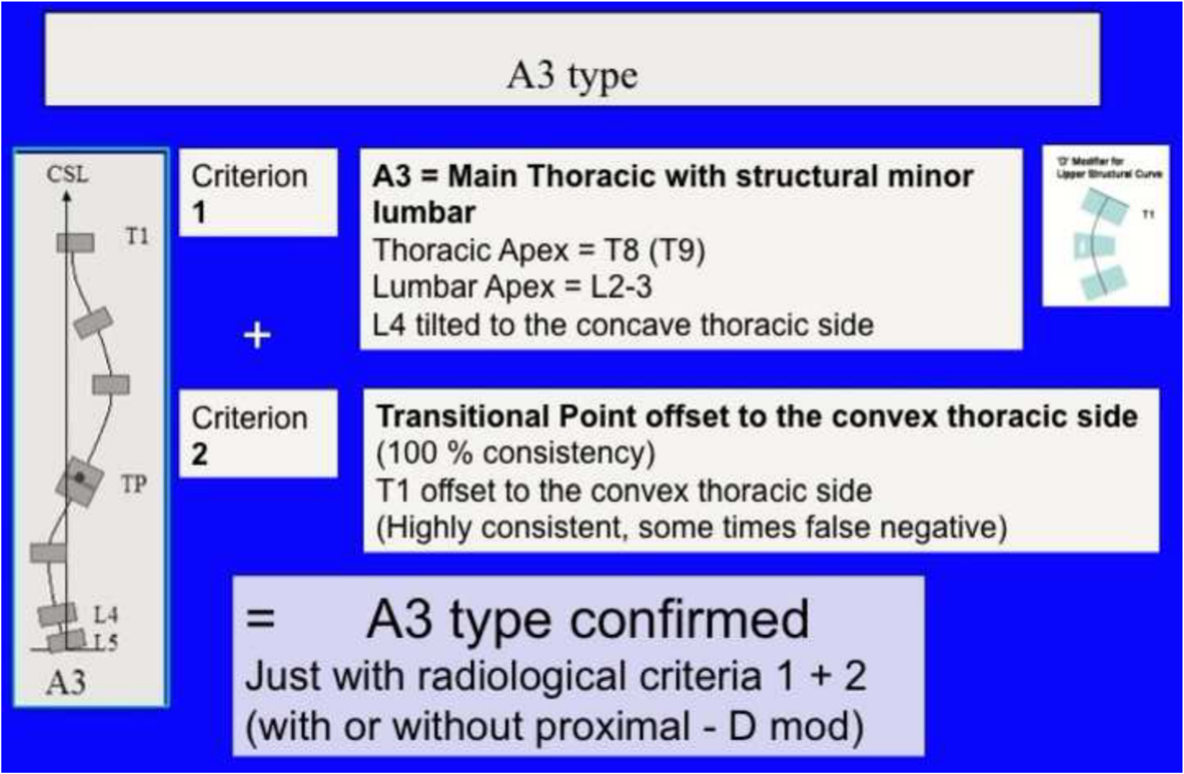
The third 3C compatible curve pattern is the composite “major main thoracic”/“minor lumbar.” Both curves are structural but lumbar is a more flexible, minor, probably secondary curve. This is called A3 type and the second radiological criteria to confirm 3C is the TP offset to the convex thoracic side, like in A1 and A2 types. Due to the lumbar structural curve, it is wrongly taken like 4C by many Chêneau followers, but a structural lumbar curve is not criterion enough to decide about using a 4C brace design. It can be combined, like most of types by a primary or secondary or iatrogenic proximal thoracic curve

**Fig. 26:**
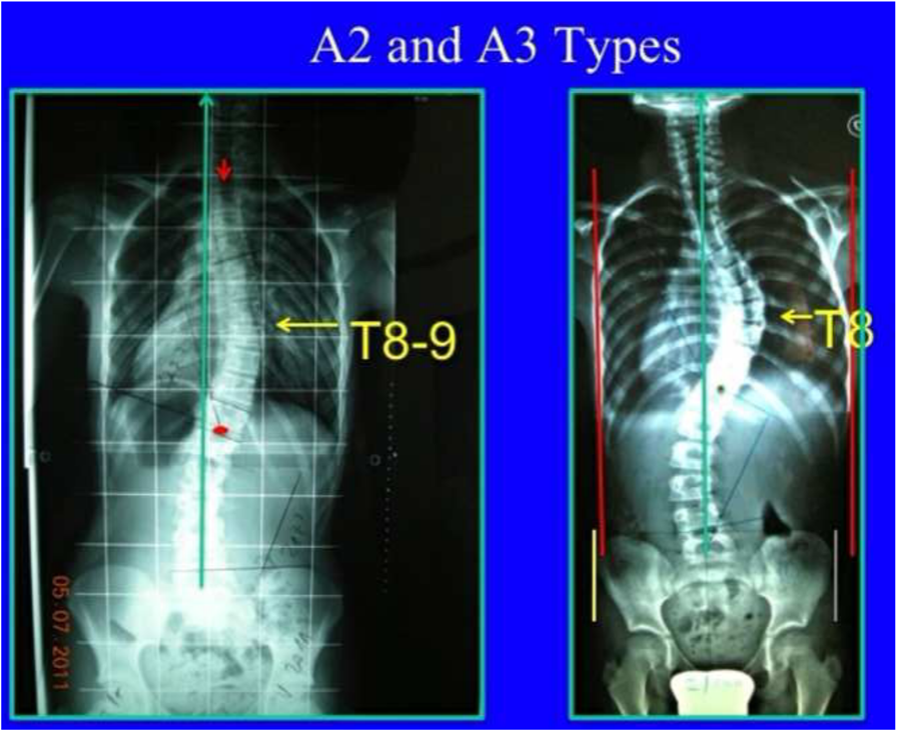
This figure shows two cases of A2 type (*left*) and A3 type (*right*). Radiological criteria can be found in Figs. 24 and 25

**Fig. 27:**
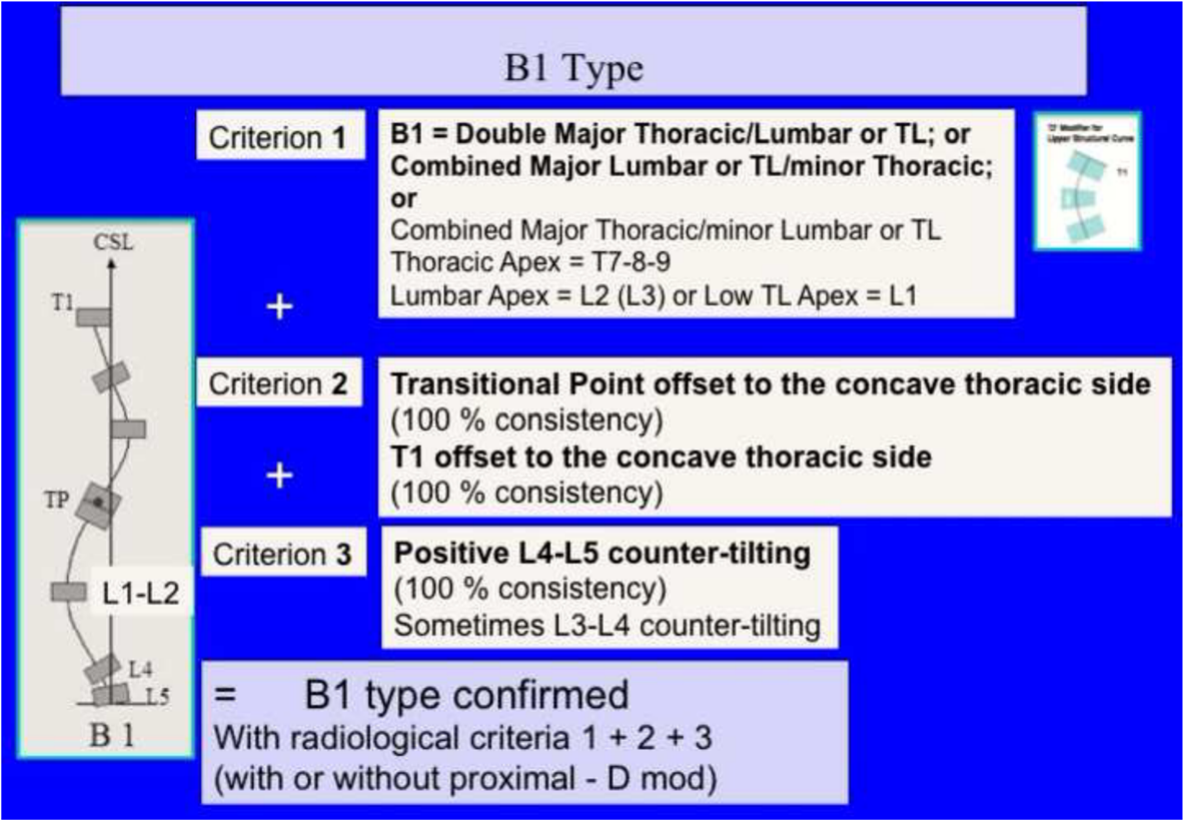
Classical double structural scoliosis, main thoracic/lumbar (apex L2) or low thoracolumbar (apex L1) is the most common 4C compatible curve pattern. This is called B1 type. It can be double major, major lumbar-low thoracolumbar/minor thoracic, or rarely major thoracic/minor lumbar-low thoracolumbar. The second radiological criterion is the TP (and T1) offset to the concave thoracic side according the CSL. A third criterion is the L4–L5 positive counter-tilting. It can be combined with a structural proximal thoracic curve (triple structural scoliosis)

**Fig. 28:**
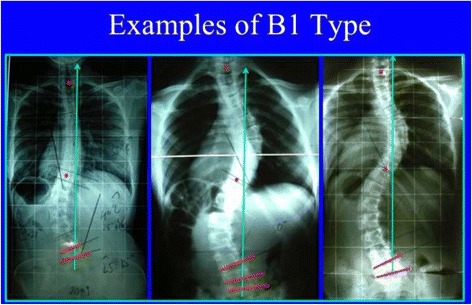
This figure shows three different cases fulfilling all the criteria for B1 type

**Fig. 29:**
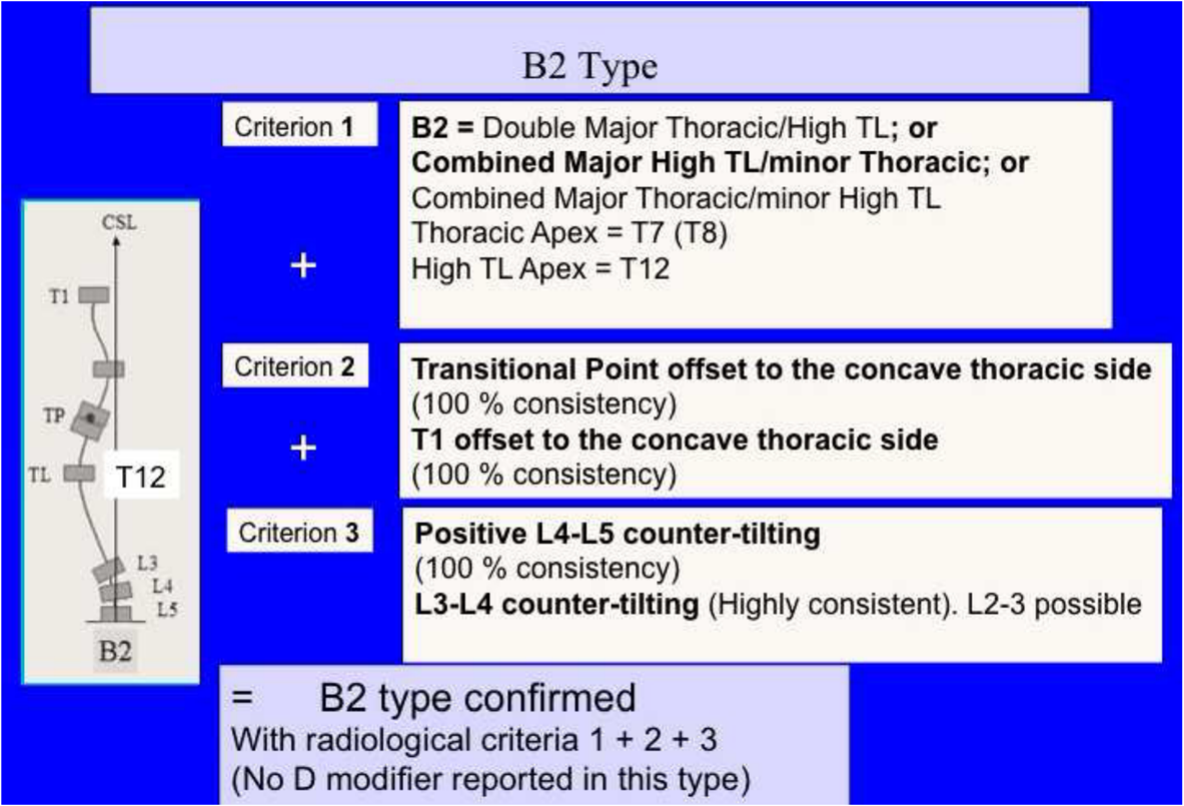
The so-called B2 type is a 4C compatible curve pattern defined by a high main thoracic structural curve combined with a long-high thoracolumbar curve, with the apex at T12. The second and third radiological criteria for 4C are also accomplished (TP offset to the concave thoracic side and L4–L5 positive counter-tilting). Very rarely it can be also combined with a short structural proximal thoracic curve (mostly iatrogenic)

**Fig. 30:**
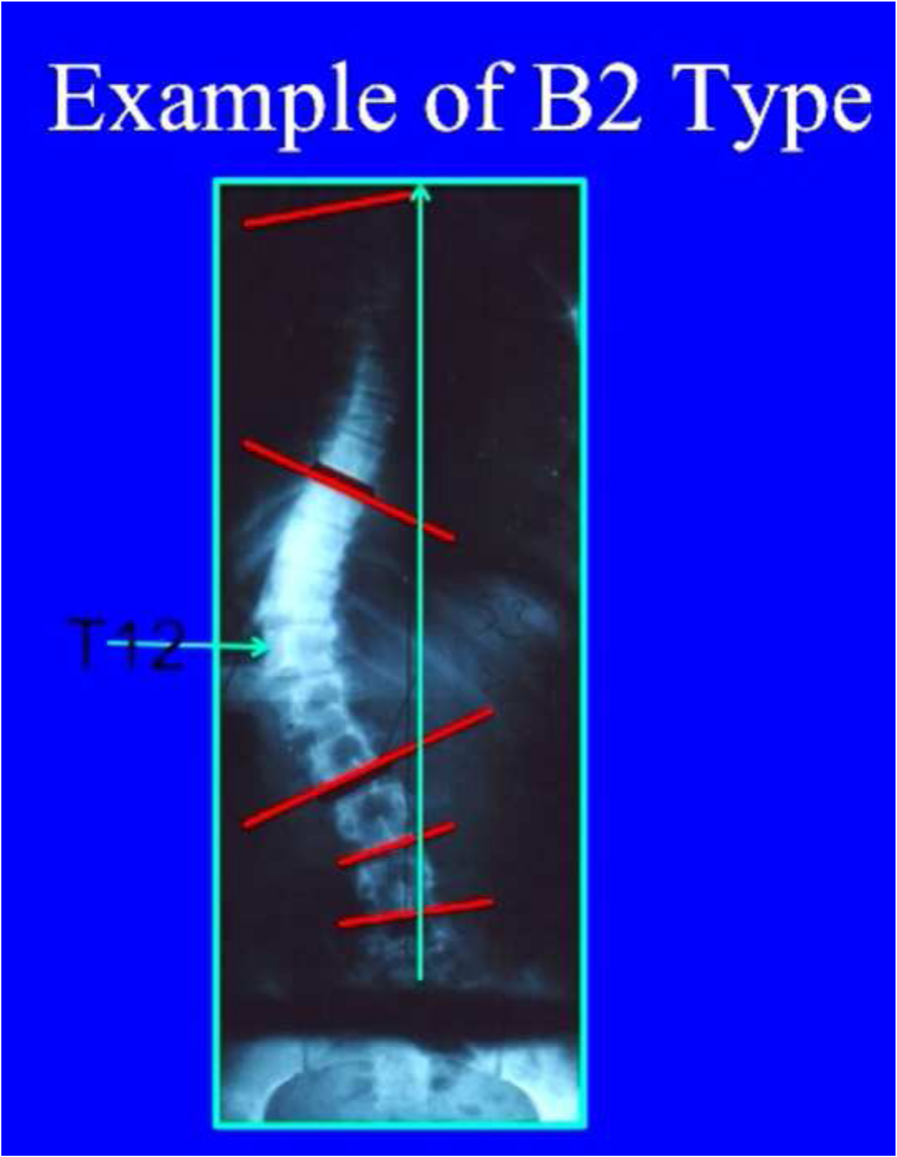
This figure shows a typical example of B2 type

**Fig. 31:**
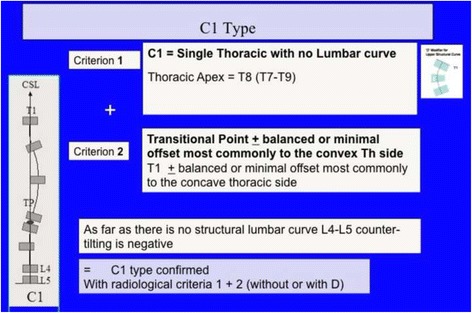
A single main thoracic curve with a more or less rectilinear lumbar spine (or minimal functional lumbar curve) is the N3N4 compatible curve pattern called C1 type. The transitional point (TP) is more or less balanced on the central sacral line. In this revisited version of the classification, we are not looking so much at T1 as an essential criterion. Many factors are associated to a non-relevant imbalance of T1, which is not against diagnosing a C1 type

**Fig. 32:**
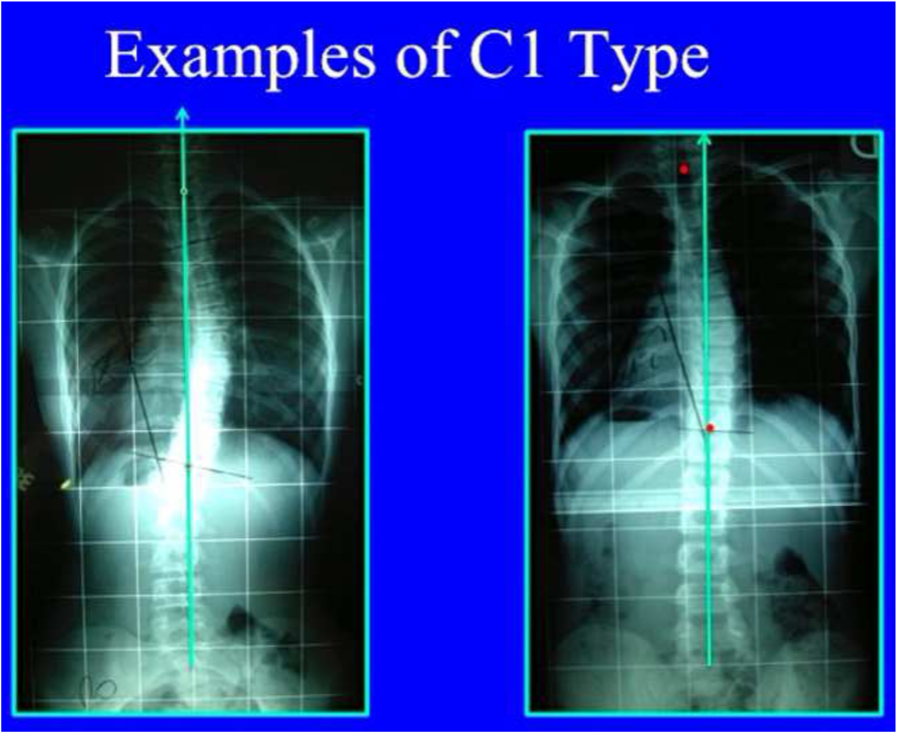
This figure shows two C1 cases. The negative T1 offset observed on the right is not a reason to reject C1 type

**Fig. 33:**
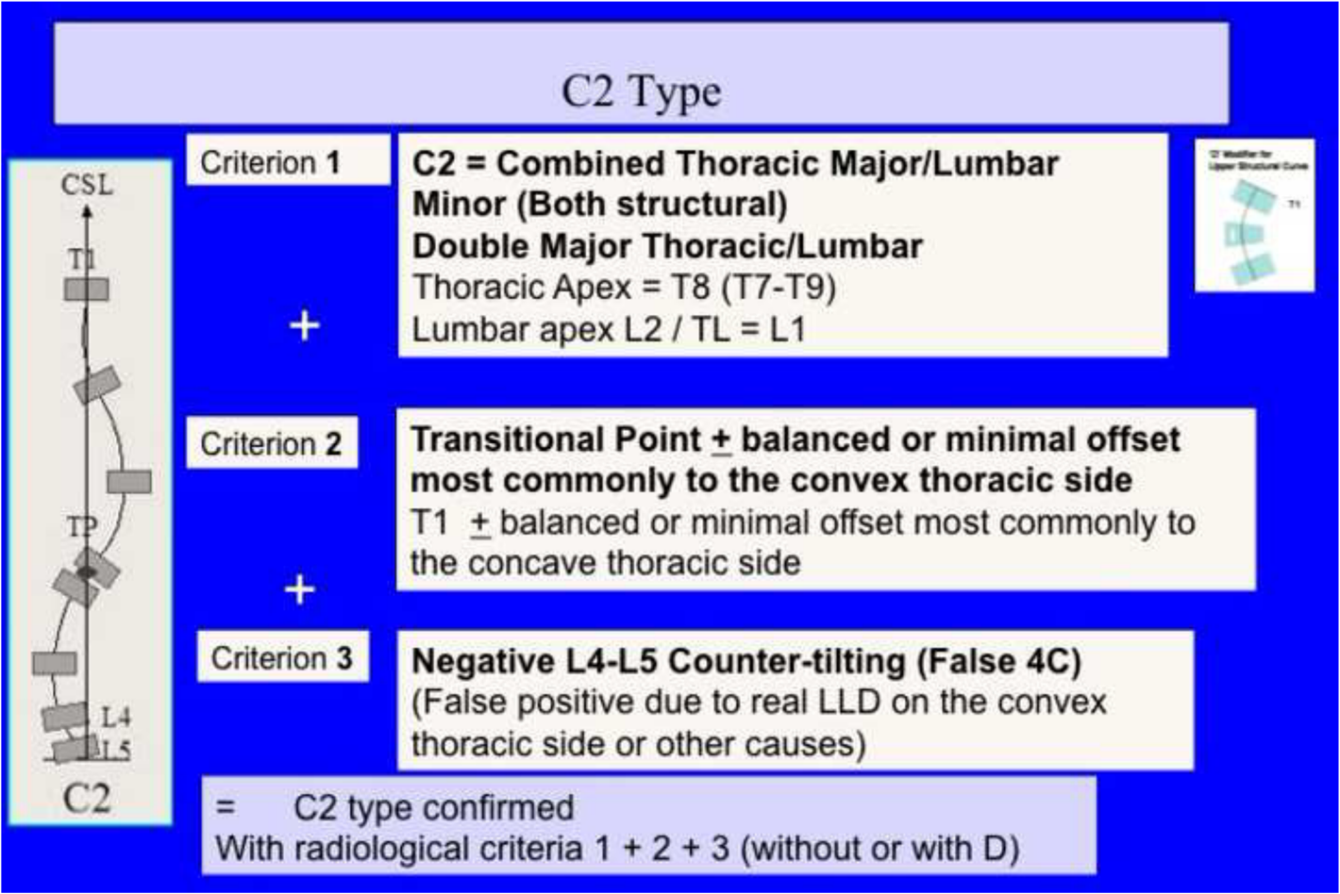
A composite structural scoliosis, main thoracic and lumbar is compatible with N3N4 and it is called C2 type. It can be major thoracic/minor lumbar or double major but we have not seen any N3N4 with lumbar structural curve where the lumbar component was major and the thoracic minor. The second radiological criterion is the TP and T1 on the CSL. Minimal offset (±4 mm) is considered not a reason to reject diagnosis of C2. The third radiological criterion is a L4–L5 negative counter-tilting (it can be observed a mild false positive counter-tilting, for example due to leg length discrepancy or pelvis asymmetry)

**Fig. 34:**
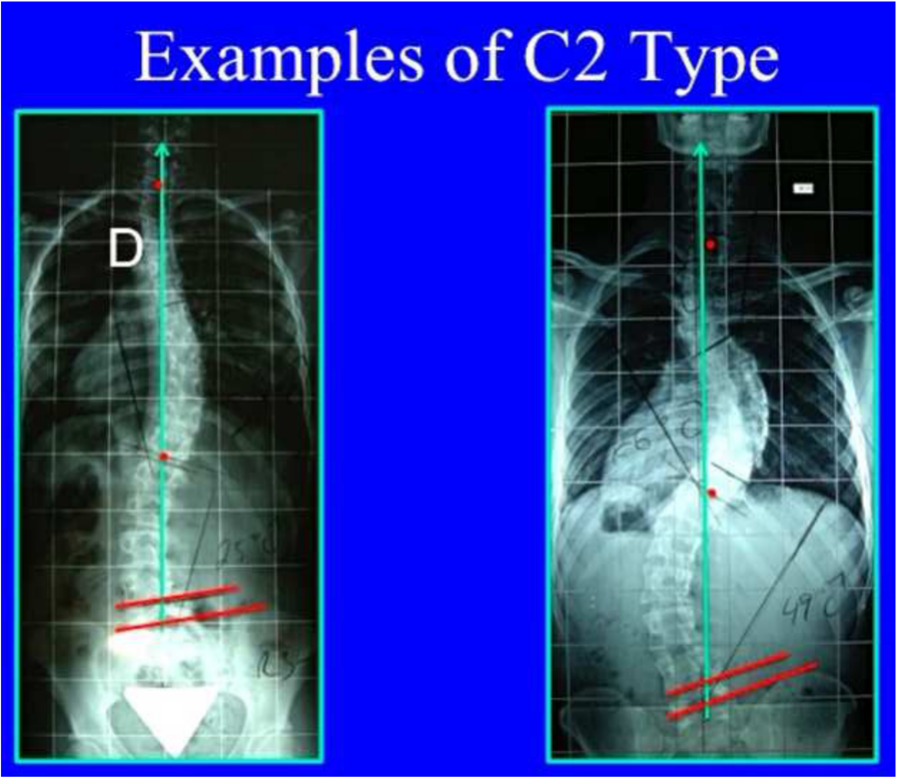
This figure shows two cases of C2 type. The second case, on the *right*, is associated to a minor structural proximal thoracic curve (D modifier), most probably an iatrogenic one, secondary to previous brace treatment

**Fig. 35:**
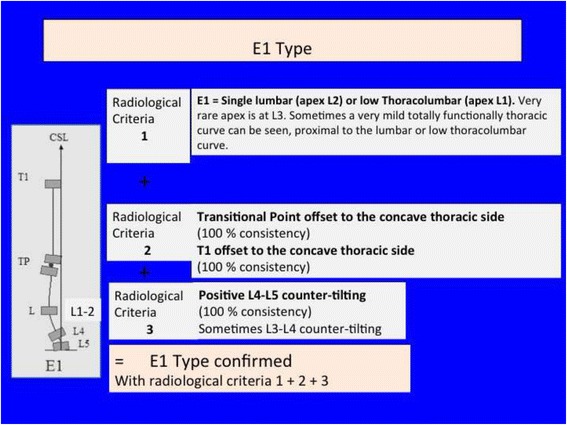
A single lumbar or low thoracolumbar curve is called E1 type. It is like a B1 type without structural curve in the main thoracic region. Criteria 2 and 3 are also like B1

**Fig. 36:**
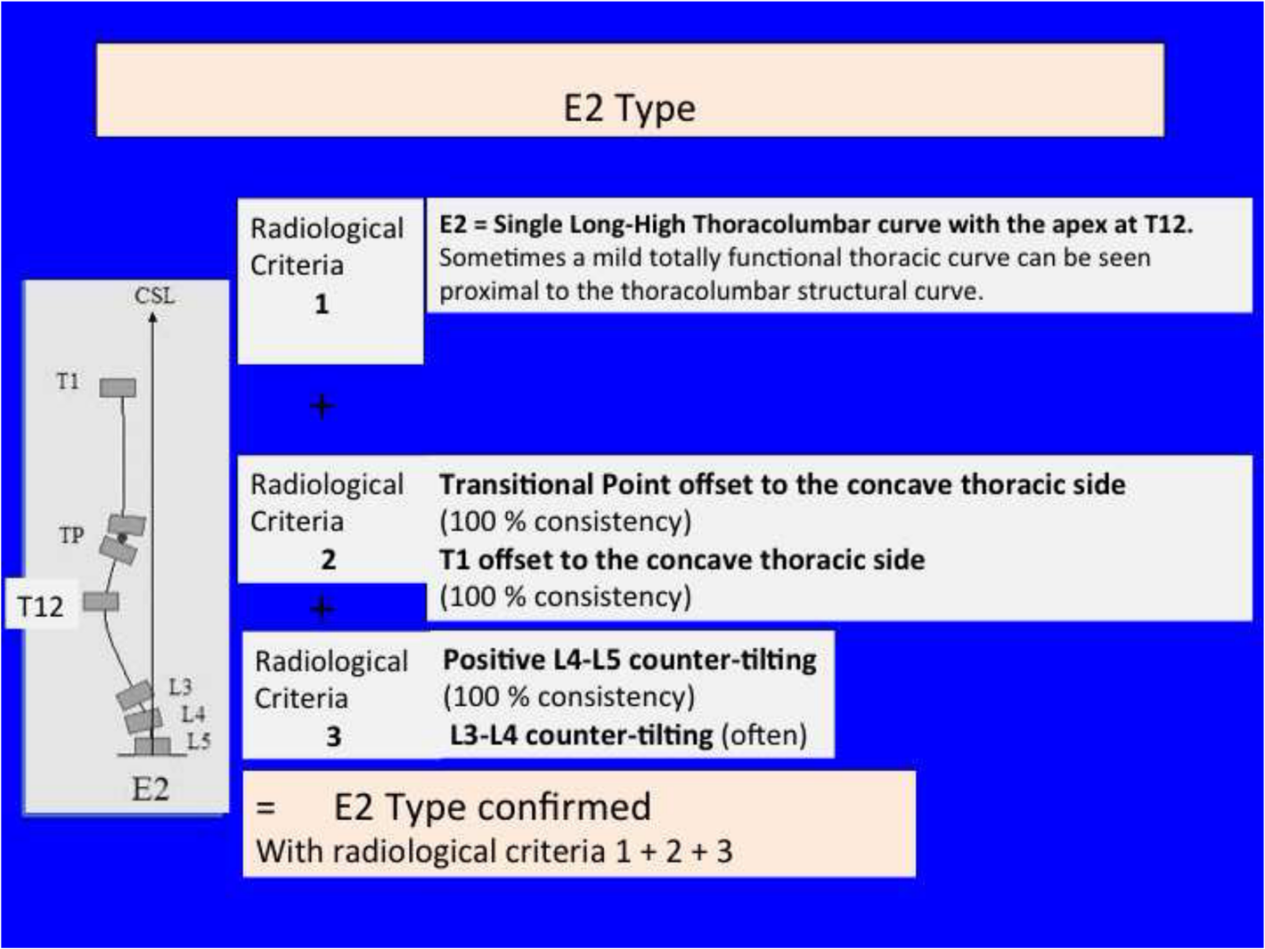
A single long-high thoracolumbar curve with the apex at T12 is called E2. It is like B2 type without structural curve in the main thoracic region. Criteria 2 and 3 are like B2

**Fig. 37:**
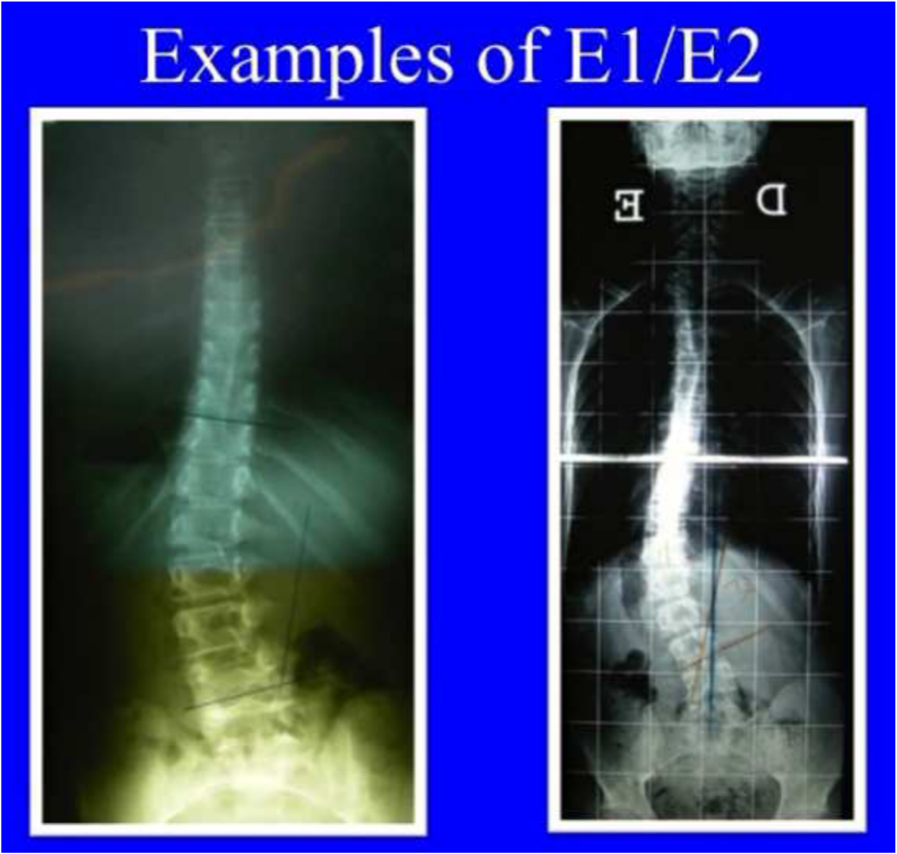
This figure shows two cases of E1 type (*left*) and E2 type (*right*)

### The brace (blueprints)

Every basic type 3C (A type), 4C (B type), N3N4 (C type), or single lumbar/thoracolumbar (E type) is treated following specific principles that were described in the previous sections. Below is the more specific application of the three-point system principle according to the different types.

#### Specific designs and construction for “A” types

**Fig. 38:**
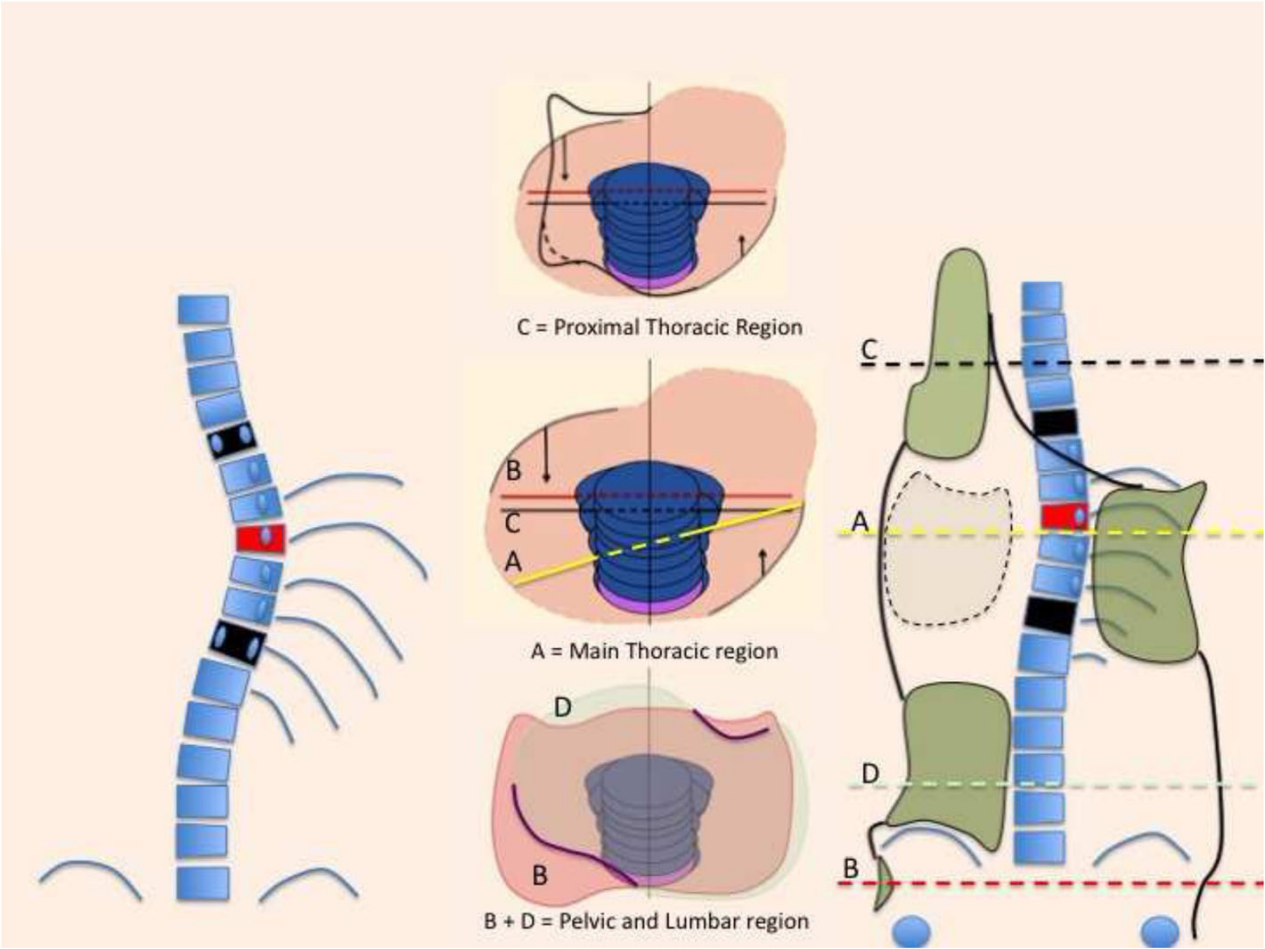
This figure shows the corrective principles for a classic single structural thoracic curve with no lumbar or mild lumbar functional curve and spinal imbalance to the convex thoracic side (defined later as A2 type in Rigo classification). “Regional derotation” affects the main thoracic region against the lumbo-pelvic region and the proximal thoracic region. The main thoracic pad (level A) is narrower than in the previous case (A1 type). Consequently, the lumbar support (level D) is wider that in the previous case. Pelvic section is asymmetric also but closed on both sides, with a short left pelvic pad (just infra-iliac) and a right counter-trochanter pad (just supra-trochanter, with a specific shape to fix down the right trochanter. This pelvic design provides a stable fixation and level of the pelvis in the frontal plane. Proximal region is exactly like in the previous case (see Fig. 6). A1 and A2 type are both considered functionally three-curve scoliosis (see Rigo classification), so these two designs are also called “three curves brace design“ (3C). When a main structural thoracic curve is associated to a structural curve (always minor and more functional) and spinal balance is still to the convex thoracic side we still classify as three curves functional type or A3 type in Rigo classification. The design for A3 is like A2, just with a stronger lumbar support. A2 type design uses a main “three-point system” and a secondary “three-point system,” formed by the most caudal counter-trochanter pad, the medium left pelvic + lumbar support and the cranial right thoracic pad

**Fig. 39:**
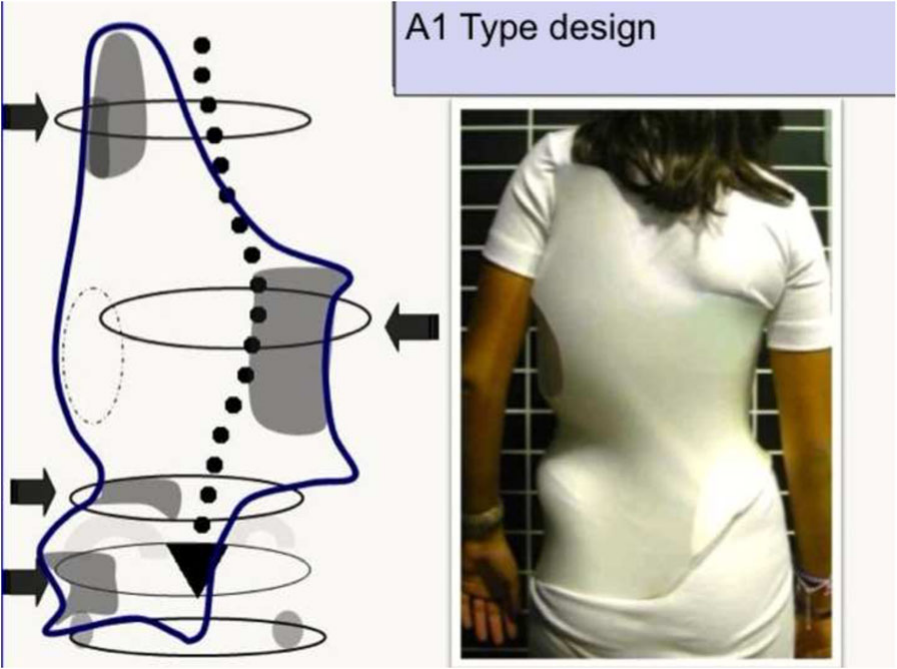
Specific brace design for A1 type. There is a “long-coming-from down” min thoracic pad, which allows using a “pelvis open” design

**Fig. 40:**
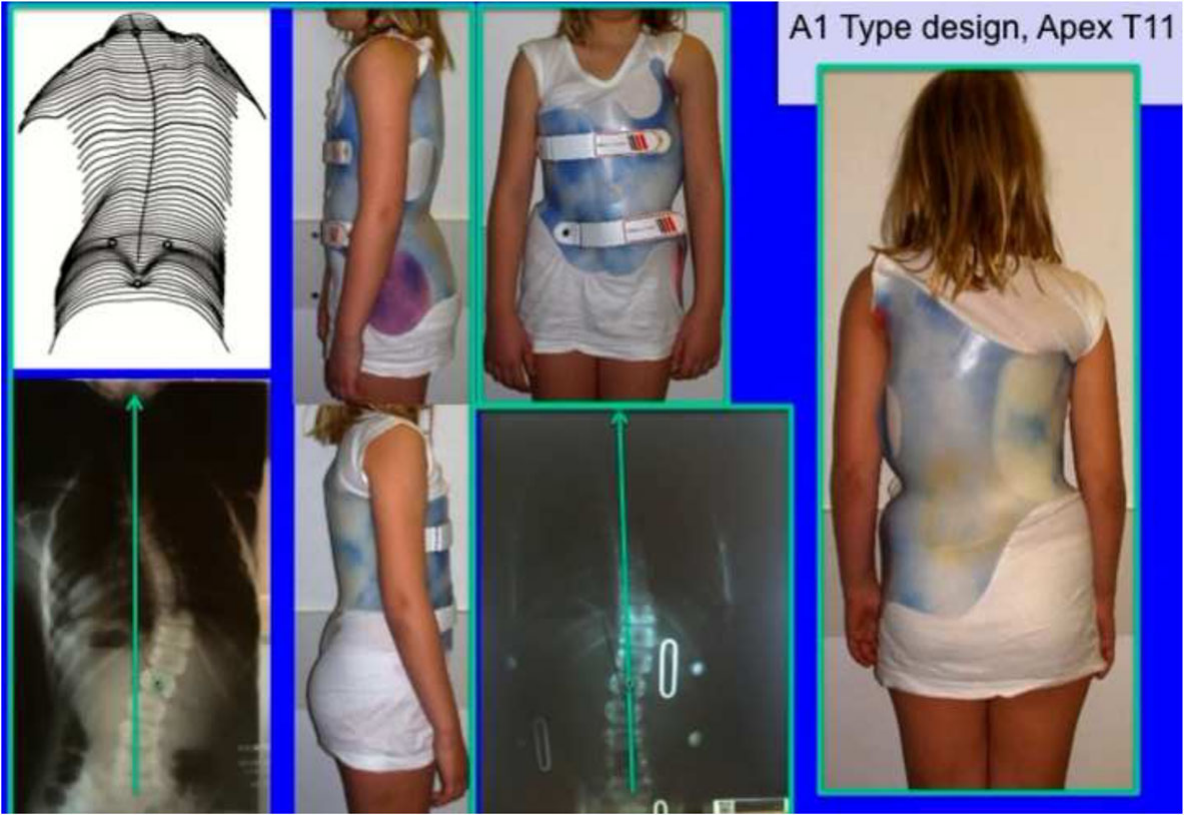
The specific brace design for A1 type has been related to the best in-brace correction as shown in this figure. In-brace correction of the Cobb angle has been reported to be 76% with this specific brace design, the highest in comparison with other curve patterns and brace designs

**Fig. 41:**
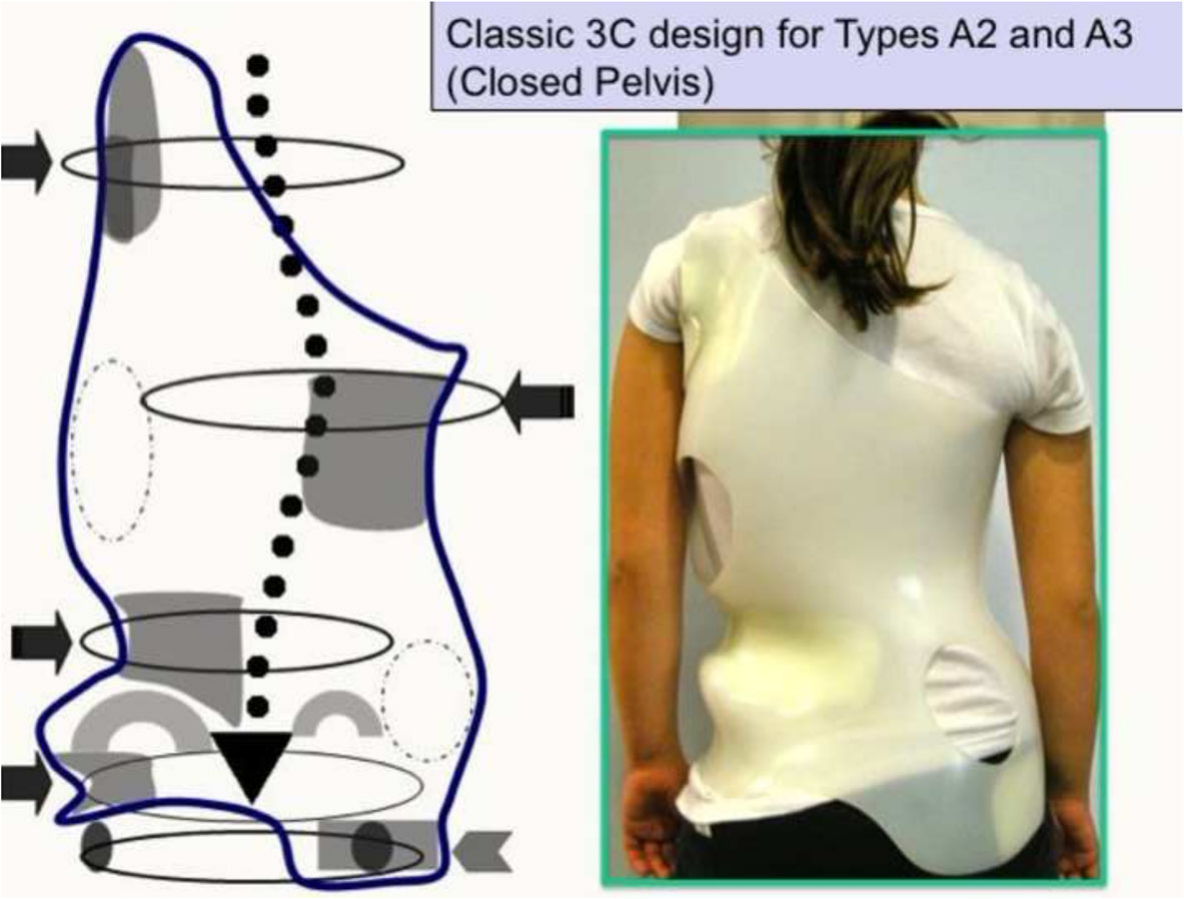
Brace design for A2 and A3 types is the same. The main thoracic pad is not so large like in A1 type. There is some expansion room caudal to the main thoracic pad, for the lumbar concavity. Pelvis has to be fixed by closing the pelvis, in order to provide a counter-trochanter pad on the convex thoracic side

**Fig. 42:**
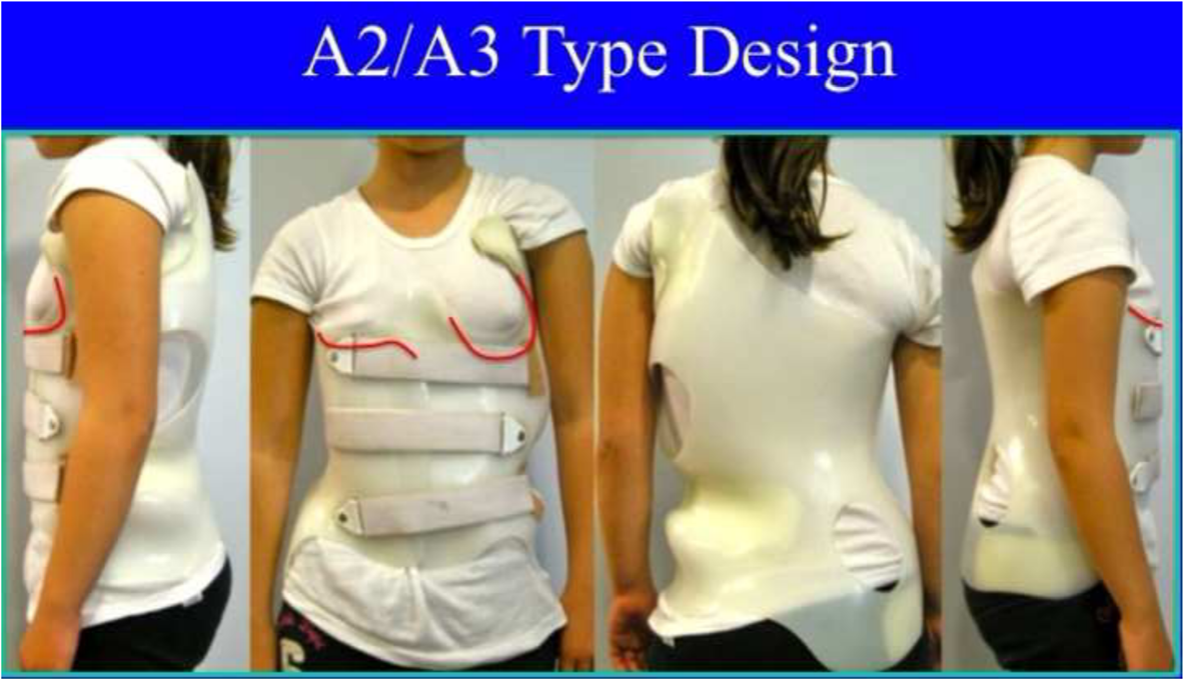
Brace design for A2 and A3 observed from ventral, dorsal, and both sides. It is important to note the sagittal balance and profile. Both sides show a different profile due to the asymmetric design. From the left it looks like the thoracic kyphosis is extended to caudal. From the right it looks like the lumbar lordosis is extended to cranial. In the middle sagittal plane the profile is more or less physiologic

The A1 type is treated with a simple main three-point system, while A2 and A3 need a secondary three-point system to complement the main one. Figures 6 and 38 show the application of corrective principles for A1 and A2/A3, respectively. Figures 39, 40, 41, and 42 show the blueprints and brace examples.

The function of the three-point system is to bring the trunk into the best possible correction in the frontal plane. For a classical right convex thoracic scoliosis, we need to translate the main thoracic region right to left in between the two caudal (lumbo-pelvic) and cranial (proximal thoracic) regions. A caudal pelvic pad with a strong dorso-lateral lumbar support on the left side, a more proximal main thoracic pad on the right side, and the most cranial pad for the proximal thoracic region on the left side form the main three-point system. When constructing these pads on the positive mould and to achieve the best possible correction, the technician should bring the left proximal pad as high and medial as possible. As described above, the orientation of the main thoracic pad allows it to work from one side and in its lateral component as a part of this main three-point system, while forming part of the dorsal component of the pair-of-force for derotation. The lumbo-pelvic as well as the proximal thoracic regions, including the shoulder girdle, have to be maintained in the best case with no rotation (i.e., the frontal plane of both regions should coincide with the frontal plane of reference). The proximal thoracic region will need a counter-rotation force integrated in the upper pad. In A2 and A3 types, a counter-trochanter pad is necessary on the right side to provide a secondary three-point system, facilitating a better postural balance in the frontal plane. To keep the patient vertical, it is necessary to stretch the soft tissues from the lumbo-pelvic concavity, which are shortened in the axial direction; otherwise, the trunk would bend to the right side due to their tension. In A1 type, the main thoracic pad is larger in the cranio-caudal direction compared with A2 and A3 types and enables the shortened soft tissues from the lumbo-pelvic concavity to be stretched more efficiently with the frontal plane translation obtained from the action of the left lumbo-pelvic pad and the right large thoracic pad, including the upper lumbar, the thoracolumbar, and the main thoracic regions. In this way, the A1-type brace can be constructed without the right counter-trochanter pad and the pelvic area can be opened on the right side.

#### Specific designs and construction for “B” types

**Fig. 43:**
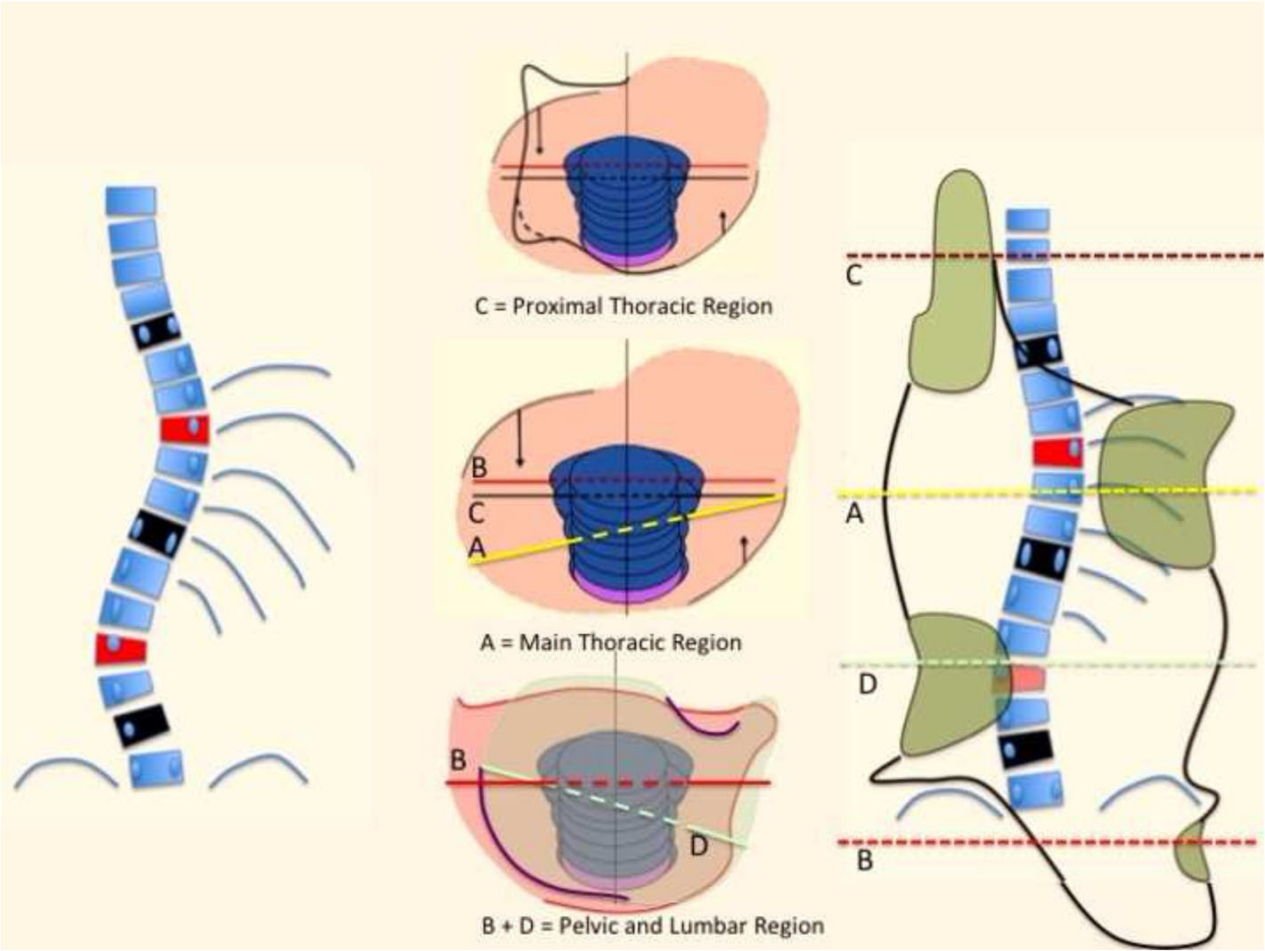
This figure shows the principle of correction for a true double structural curve. Most but not all the double structural curves are classified as “four-curve pattern” (4C) or “B type” (B1 and B2). The objective criteria to classify 4C (or B types) can be seen later in the main text and some more figures. This current figure is about a classical B1 type, with the apical vertebrae of the right main thoracic curve at T8 and the left lumbar curve at L2. “Regional derotation” is applied here at these two regions. The main thoracic region is over-derotated to the left (*yellow line A*) like in previous cases. The lumbar region is over-derotated to the right with the combination of a couple of forces formed by a real left lumbar pad and a right low abdominal pad (*green line D*). The lumbar pad is dissociated or uncoupled from the pelvic region (it is not just a lumbar support coupled to the pelvis section) and approaches the lumbar convexity, reaching the maximum pressure at the apical level, leaving room down. Pelvis region has to be translated to the left, bringing also the lower lumbar vertebrae to the left, to the provided room caudal to the lumbar pad. Pelvis is not only translated but also derotated to the left (not over-derotated but just derotated to 0°, fixing the pelvis region in the frontal plane of reference—*red line B*). Two main “three-point systems” are formed with the lateral component of all the pads. The right pelvic pad, the left lumbar pad, and the right thoracic pad form the lower “three-point system”

The B type is treated with two main three-point systems (the principle of correction can be seen in Fig. 43). The caudal system is designed to correct the lumbar or thoracolumbar structural curve and a more proximal system acts on the main thoracic curve. In B types, the pelvis and lumbar regions are not coupled, so they cannot be corrected together against the main thoracic region, but one against the other. Thus, for a classical left lumbar/thoracolumbar combined with right thoracic, the most distal pad of the caudal system is located on the lateral aspect of the right pelvis, between the iliac crest and the trochanter; the medium pad pushes on the left lumbar or thoracolumbar prominence; and the most proximal pad puts pressure on the thoracic rib hump. When doing the modification of the positive mould, pelvis has to be translated to the concave thoracic side (right to left for a right thoracic/left lumbar) in about 10 cm or more, while lumbar pad is built gently to offer a stopping point. For the proximal three-point system, the most distal pad presses on the lumbar or thoracolumbar prominence, the medium pad pushes on the thoracic rib hump, and the proximal pad puts pressure on the left proximal thoracic region. The construction of this proximal system in B type is similar to the A type, although it may be shorter in the cranio-caudal direction.

The B-type brace can be built with the pelvis open in most cases, but it may be necessary to use a counter-trochanter pad on the left side (for the example used here of right thoracic/left lumbar or TL). However, the decision about when to use an open pelvis or when to close it to provide the counter-trochanter pad is not based here, as in A type, on the diagnosis of a particular subtype. Both B1 and B2 can be built with a complete pelvis or with an open pelvis. We cannot give an evidence-based explanation about the cause or causes of the frontal plane imbalance in B type scoliosis, so we cannot explain why some patients attain in-brace balance of T1 and TP on the CSL, accepting with no relevant problems the open pelvis design.

**Fig. 44:**
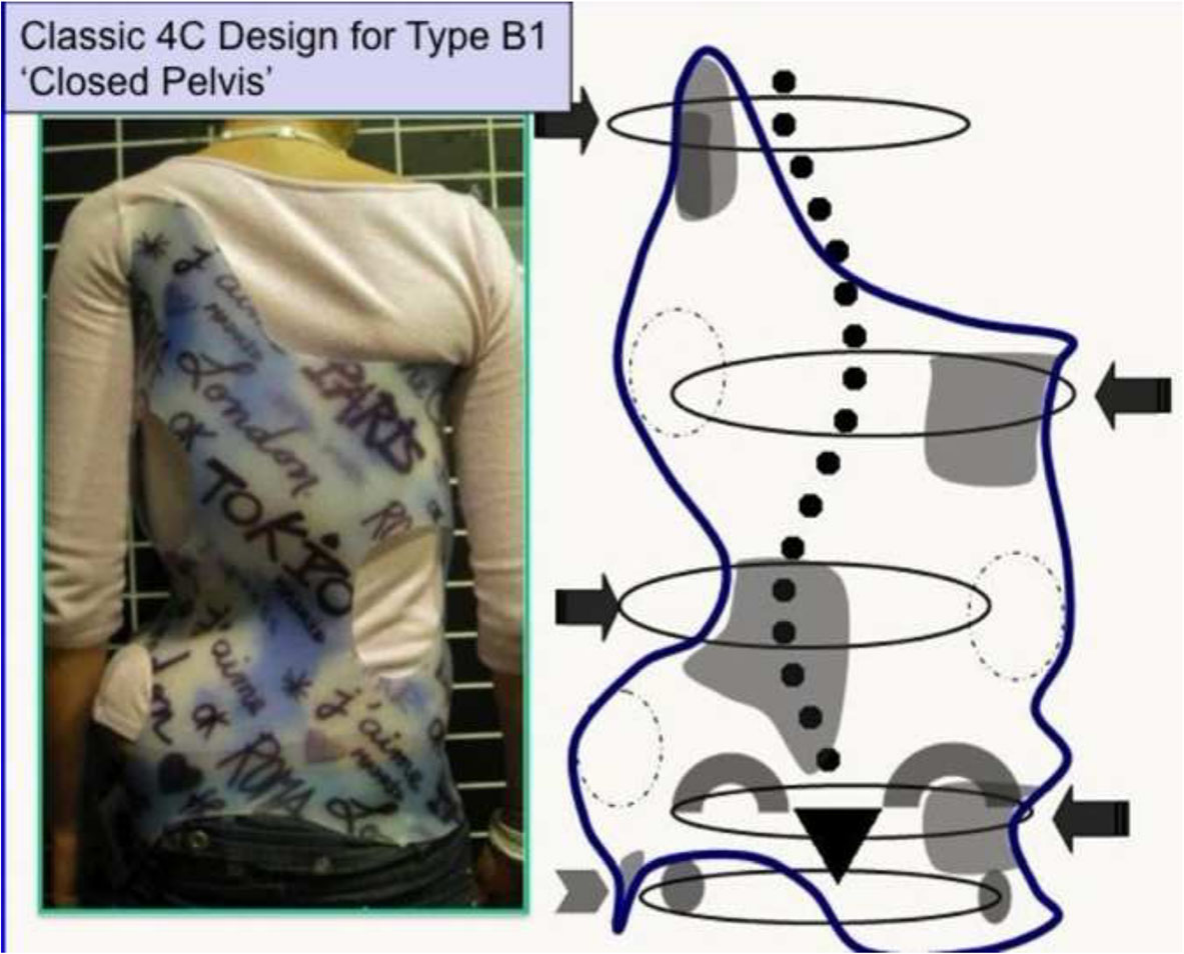
Brace design for B1 type, with pelvis closed to provide a left counter-trochanter pad

**Fig. 45:**
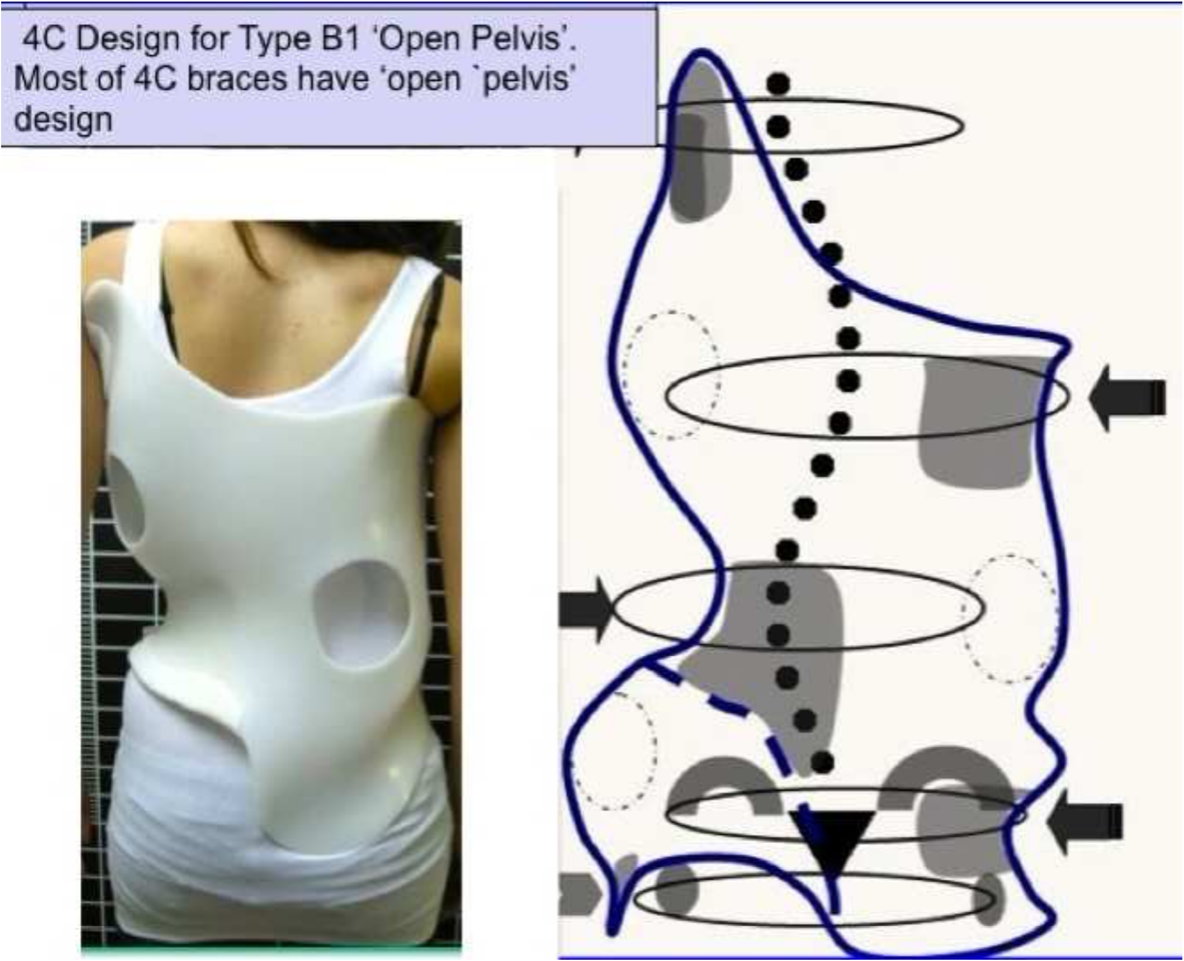
Brace design for B1 type, with pelvis open. Nowadays, we do most of B1 braces following this design

**Fig. 46:**
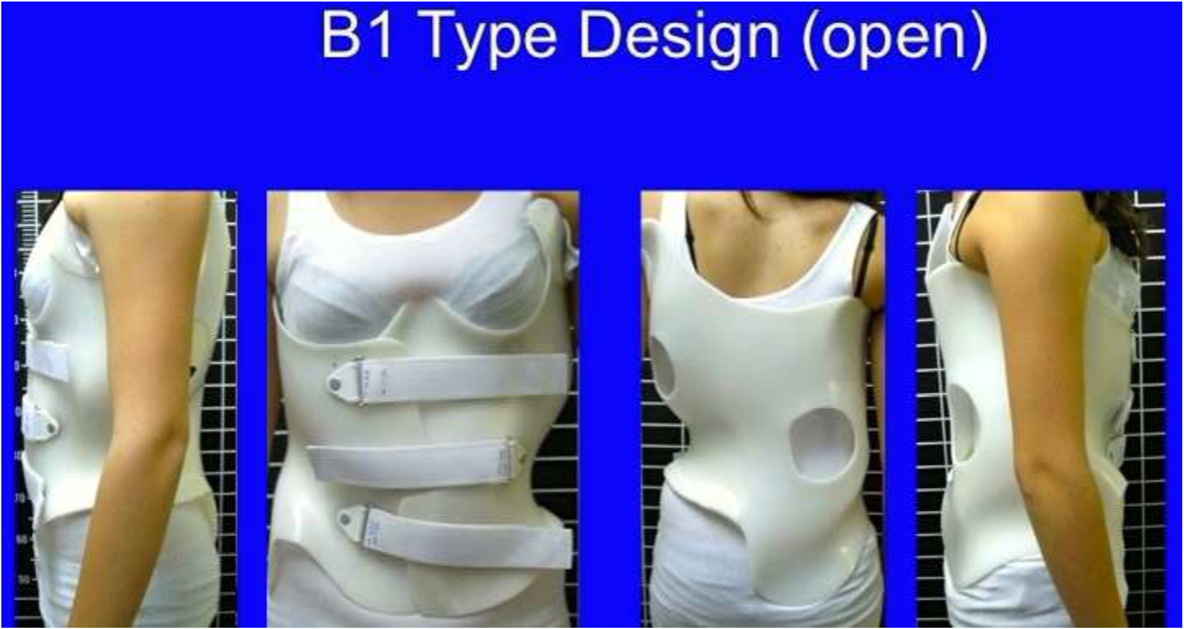
Brace design for B1 type observed from ventral, dorsal, and both sides. Both sides show a different sagittal profile due to the asymmetric design. From the *left* it looks hyper-kypho-lordotic, from the *right* it looks hypo-kypho-lordotic. The brace is more or less physiologic just in the middle sagittal plane

**Fig. 47:**
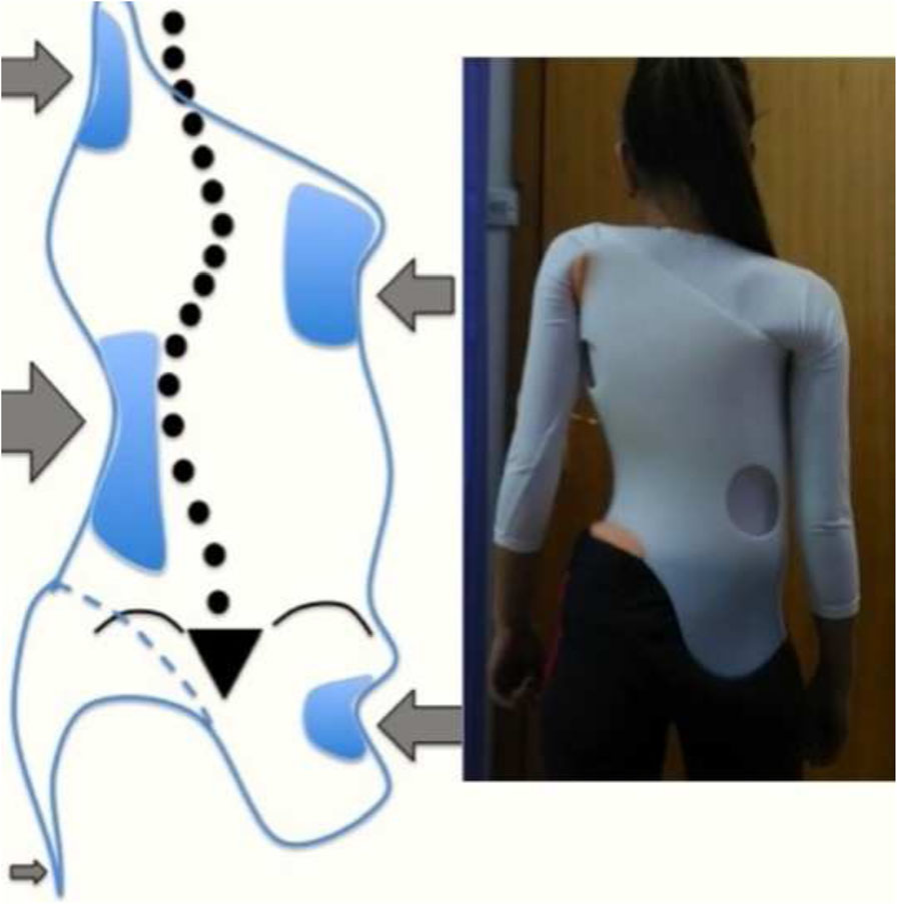
Brace design for B2 type. There is a large 3D-shaped thoracolumbar pad, very difficult to perform when correcting the positive mould by hand. On the *right*, a B2-type brace made by the junior author MJ (left thoracic/right TL curve, mirrored)

Blueprints to treat B1 and B2 are the same (Figs. 44, 45, 46, and 47). The main difference is the size and shape of the lumbar/thoracolumbar pad. B1 is a more or less wide pad in the cranio-caudal direction, depending on the apical level, the most typical L2 and L1. We design a real pad, which brings, with a highly anatomical shape, the whole prominent region to a more ventral and medial position. Covering the lower ribs with the pad has historically not been a problem when allowing enough room ventrally at the same level and dorsally in a lower level, and allowing pelvis ante-version, especially at the gluteus region on the same side. The pad in B2 is a very large thoracolumbar pad, which has to provide the maximum derotational effect at the T12 level. The pad has a very accurate and difficult-to-achieve 3D shape and orientation, contributing in its lower part to create lumbar lordosis, in its upper part to allow thoracic kyphosis, and as a whole to maintain the thoracolumbar region as a geometrical transitional region.

#### Specific designs and construction for “C” types

**Fig. 48:**
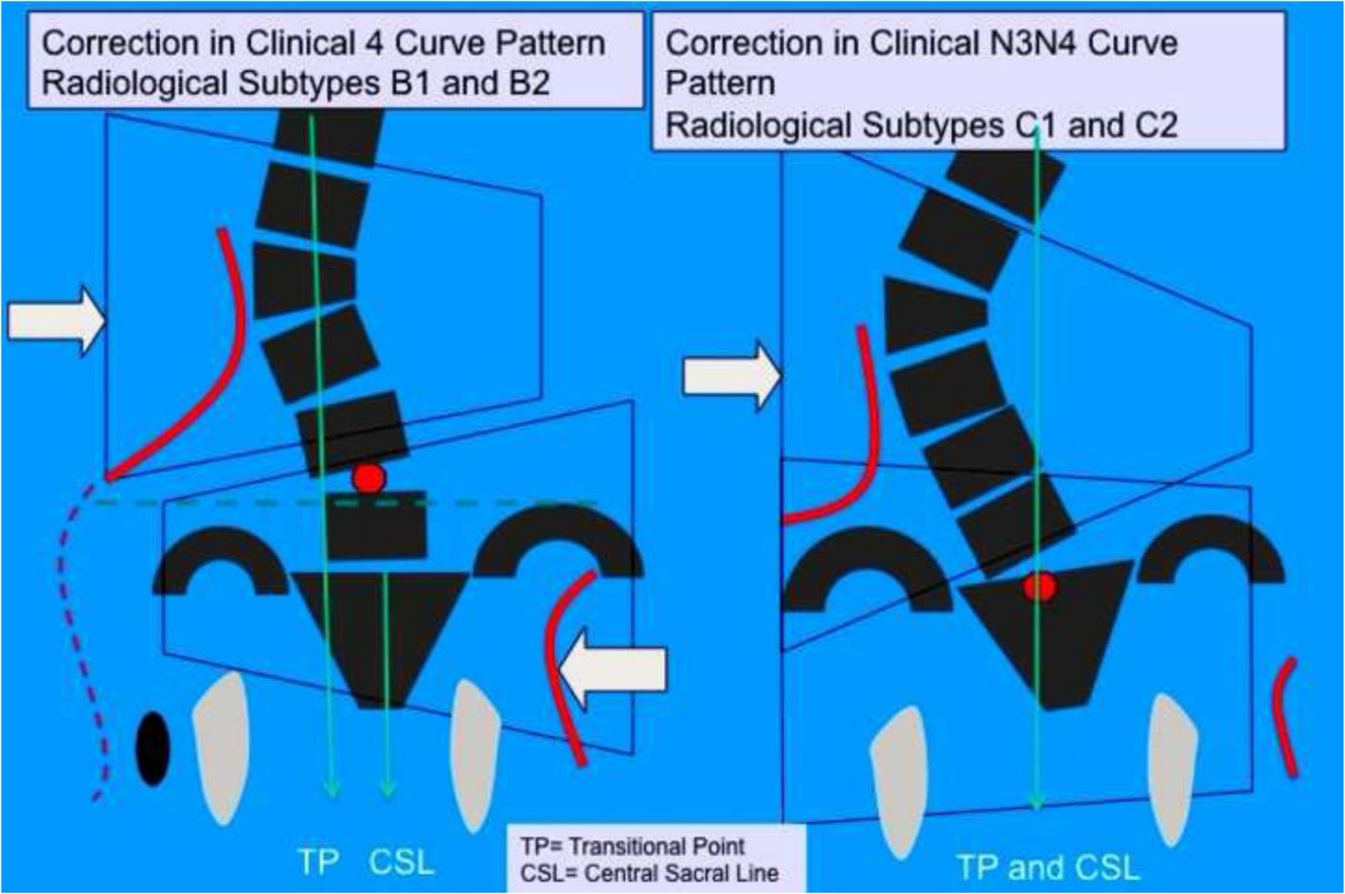
This figure shows the main difference in designing B and C types. In B type, the pelvis region is first translated around 8 cm, taking off plaster from the pelvis region at the convex thoracic side. This is not necessary in C type. Later, the pads are designed. In both types, pelvic pad is very similar, if perhaps it works lightly more caudal, just supra trochanteric, in C type. This pad braces the whole hemi-pelvis on the convex thoracic side, as it is extended to dorsal. On the concave thoracic side, B type presents a real lumbar pad reaching its maximum pressure at the apical level of the lumbar curve and approaching this level coming inclined from caudal to cranial and from lateral to medial. This pad works as a counter-stop point at the apical level against the pelvis a low lumbar region translation. The room caudal to the apex is necessary allowing translation. In C type, pelvis cannot be translated because it is a coupled lumbar region, so correction can be only achieved by keeping the pelvis stable and pushing the lumbar curve from caudal to cranial. The lumbar support goes practically horizontal from lateral to medial and reached the maximum pressure in the lower lumbar hemi-convexity

**Fig. 49:**
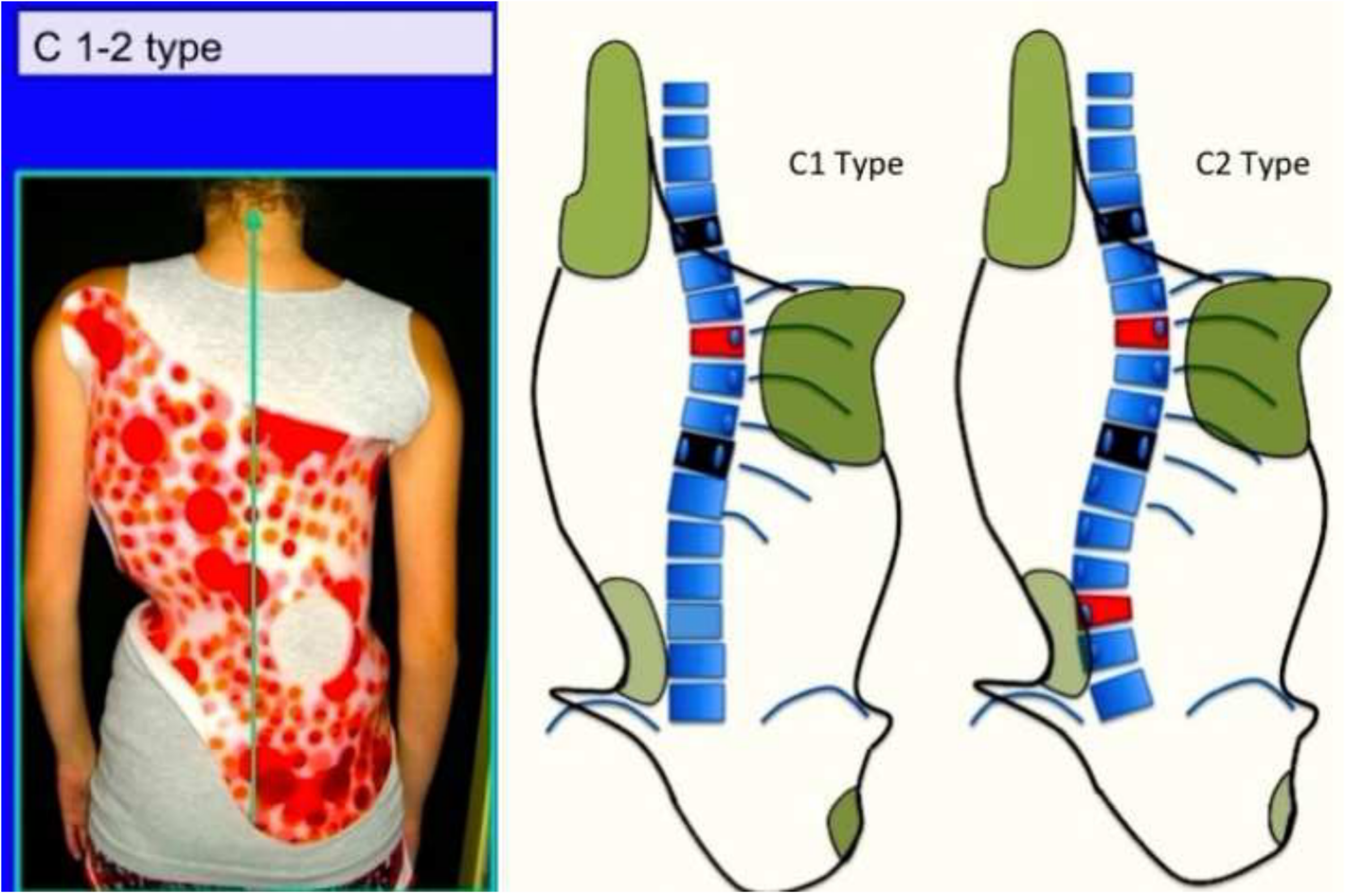
An example of C-type brace can be seen on the *left side* of this figure. No significant difference exists between the C1 and C2 brace design, as showed on the *right side* of the figure

C1–2 braces are built now most with the pelvis open, like 4C, at the concave thoracic side, but while modifying the positive mould, the pelvis section is considered the neutral caudal reference and consequently shall not be translated like in 4C. Pelvis can be fixed between a lumbar support and a counter supra-trochanter pad on the convex thoracic side. Although it looks like in B type, it is not. There is a significant difference in the design of the B lumbar pad and the C lumbar support as can be seen in Figs. 48 and 49. Lumbar support and counter-supra-trochanter pad form a system to block the pelvis in a stable position, balanced on the polygon of sustentation and well oriented in the frontal plane.

#### Specific designs and construction for “E” types

**Fig. 50:**
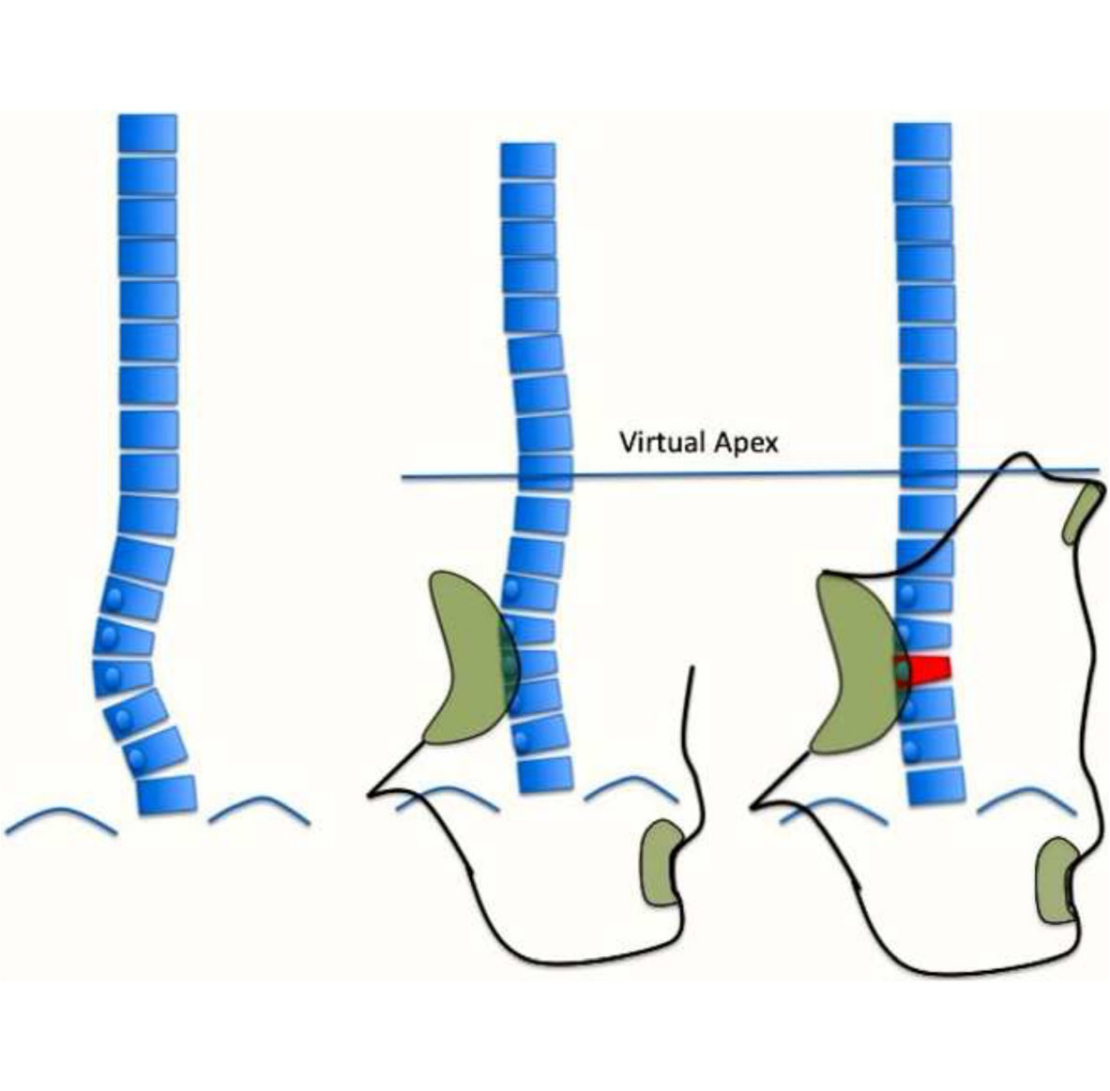
Brace design for E type. The difference between E1 and E2 is the same difference in the size of the main pad. A long-high thoracolumbar with apex at T12—E2 type—needs a larger pad in comparison with E1. The main thoracic pad works mainly as a counter-rotation force and a counter lateral to medial force worn the virtual apex of the compensatory functional thoracic curve. Although this curve does not exist originally it tends to appear when the structural lumbar or thoracolumbar curve are corrected

**Fig. 51:**
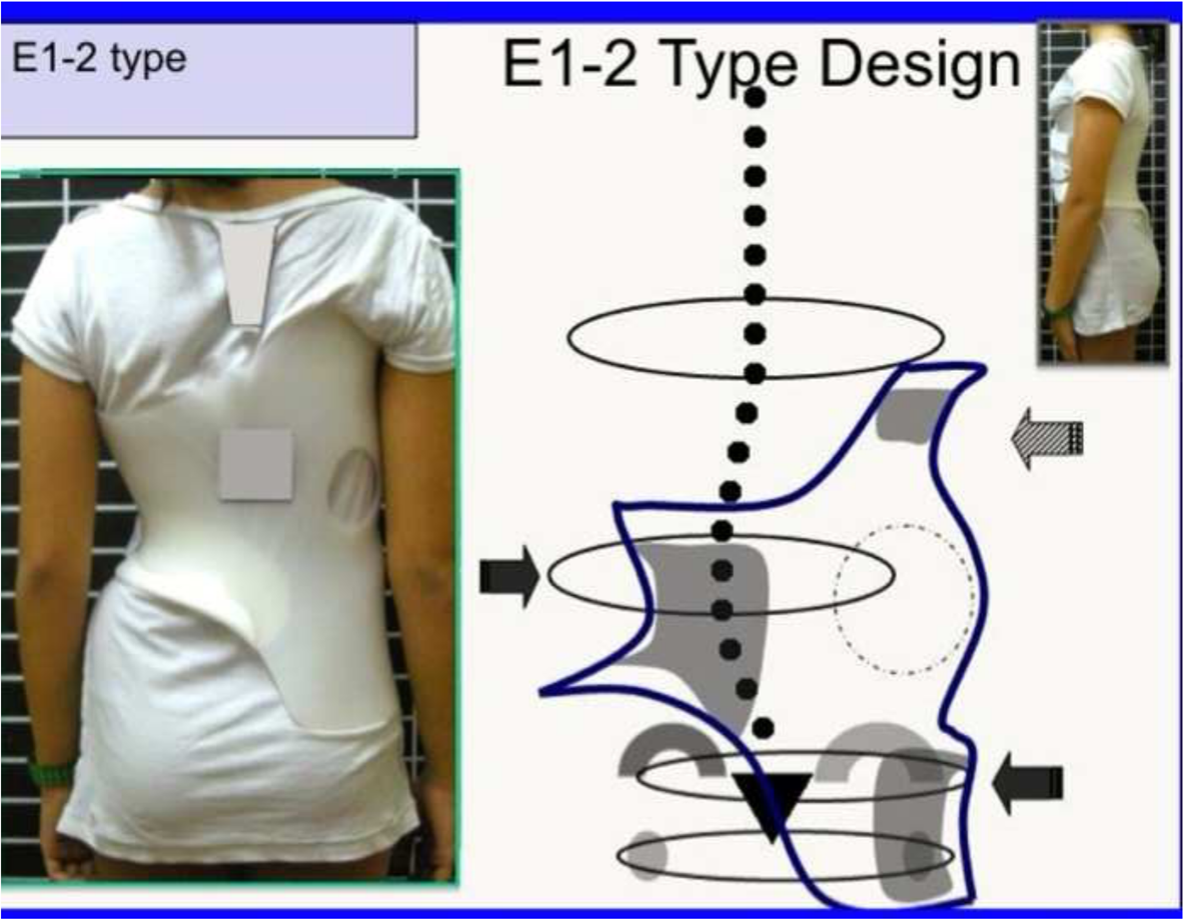
This figure shows an example of E1-type brace

The brace design for E1 and E2 is a short one, with a single three-point system to correct the single lumbar/thoracolumbar curve. At lumbo-pelvic level, the brace has exactly the same design as B1 and B2, respectively, with or without the counter-trochanter pad. However, the short brace is not just a long brace where the proximal thoracic pad has been eliminated. Since there is no structural main thoracic curve, it is not necessary to design a “deflection-derotation system” acting on the main thoracic region. At the main thoracic region, simply a counter-thoracic lateral pad is necessary to prevent the secondary formation of a functional thoracic curve, which could become structural later. This counter-thoracic pad has to act from lateral to medial and just caudal to the virtual apex of the secondary curve produced in the main thoracic region. Dorsally, the brace can extend from cranial to the virtual apex to produce a stopping counter-rotation effect, but laterally it has to be cut caudal to the level of the virtual thoracic apex; otherwise, it will facilitate the formation of a secondary curve in the main thoracic region that can become structural and potentially progressive (Figs. 50 and 51).

#### How to prescribe the brace?

It is a more or less generalized rule in this field that different curve patterns require different brace concepts or orthopedic products, which can be prescribed according to the doctor’s specific knowledge, experience, and preferences. However, although a prescription of “Chêneau brace” exists with its own reference code number in the list of orthopedic products covered by the public health system in many countries, this does not guaranty the minimum quality and standard required treating the patient effectively, efficiently, and safely. RSC® has a number, but only in Germany, and cannot be used by custom-made braces and other CAD CAM systems. Thus, the Rigo-Chêneau brace can and must be prescribed under the name “Chêneau brace” according to Rigo principles and classification. However, the so-called Chêneau brace is a “highly specific corrective device” that has to be built not only according to the principles for each curve pattern but also taking into consideration individual factors like the patient’s morphology and correctibility. The whole concept was inspired in the plaster cast technique applied by the old masters, so it is not possible to build a good standard Chêneau brace with repeatability and consistency without specific and deep knowledge of the 3D nature of idiopathic scoliosis and extensive experience with scoliosis correction. A good Chêneau brace can only be constructed and fitted by an experienced medical doctor with specific knowledge of scoliosis correction, assisted by an orthotist, or by a highly educated and experienced orthotist working in full collaboration with a multidisciplinary team coordinated by a medical doctor, who is also highly educated in this technique, and a scoliosis physiotherapist, who provides his/her specific knowledge about the patient’s correction throughout posture and movement. Historically, the contamination by other brace concepts has produced unacceptable failures in the technique.

#### How to build the brace?

Following are the three different ways to construct the Rigo-Chêneau brace:

1. Classical hand-made technique, which is based on the modification or correction of a positive mould of the patient’s trunk from a negative mould taken directly on the patient using plaster bands. Eventually, the positive mould can be reproduced by CAD CAM after a laser capture of the patient’s trunk. The modification of the positive mould consists in shaping all pad areas and expansion spaces according to the desired curve pattern-specific design. It is generally assumed that pad areas are built by removing plaster from the positive, while adding plaster forms the expansion spaces. However, depending on the design, it is common for pad areas to be shaped by adding plaster and for spaces to be created by removing plaster. The brace itself is built by modeling a thermoplastic structure, which is commonly 4 mm of polypropylene-copolymer, on the modified positive mould. The knowledge about how to affix the plastic to the mould is part of the general knowledge of a certified orthotist and is not explained in this paper. The trim lines, however, are essential to the success of the brace and are determined during the fitting process. Improper trim lines can destroy a well-constructed brace. Generally speaking, the plastic is prepared by the orthotist for the first fitting such that all trim lines are slightly higher (on the top) or lower (on the bottom) than necessary for the initial fitting.
2. Similar to the classical procedure, the CAD CAM system enables the orthotist to laser capture the patient’s trunk and use a software program to modify the virtual mould. The program offers a partially predesigned mould according to the two basic types: 3C and 4C. The authors are not familiar with this procedure and, although many orthotists use this system to build Chêneau-type braces and derivatives, they know of no orthotist who uses it to specifically build a Rigo-Chêneau-type brace.
3. Using a CAD CAM system from a predesigned library, a fully predesigned mould is selected and modified according to the patient’s specifications, including static as well as dynamic measurements. The library can be based on a somewhat complex and complete set of models and the selection can be based on a somewhat complex and complete classification.

This paper describes the principles that provide a better understanding of how to build a custom brace. CAD CAM procedures are not described herein.

#### How to check the brace?

In our particular case, the orthotist and MD make the first fitting. First, in case the patient is fitted for the first time (no previous brace treatment), the doctor explains the brace objectives to the patient. Depending on curve flexibility, the patient feels more or less pressure from the pads. The more rigid the curve, the more pressure the patient will note, and the more marked the trunk posture will be changed. From one side, pressure produces physical discomfort and, depending on the patient’s sensitivity, this first contact with the brace can be a determinant for brace acceptance. Alternatively, the change of trunk posture creates a neurological discomfort by changing suddenly, without adaptation, the body schema. As a result, the patient must be managed calmly and respectfully. For the first trials, the orthotist will help the patient put the brace on, but the brace will be closed only with the patient lying in the supine position. Typically, the brace is finished with three straps in the front, but for the fitting phase, two straps are adequate. The two straps are closed gradually until the right side of the plastic overlaps the left side (for a right thoracic curve). The brace has been built such that extra volume will guaranty the life of the brace for at least 10 to 12 months, even in the accelerated growth phase before and during the peak of growth. A well-constructed brace adapted after menarche should usually work until the end of treatment. The brace is fitted alternating the caudal and cranial strap, and once it is finally fixed in the lumbo-pelvic region, we ask the patient to breathe deeply while observing thorax expansion. At a certain point, the patient has to fight against the brace to make a full inspiration. When this happens, we use the exhalation phase to completely fasten the straps to their final position and mark the straps with the “maximum” closing point. In flexible curves, this position is well accepted from the beginning. In rigid curves, fitting the brace at the maximum position can be stressful for the patient, so we settle on a “minimum” fitting point. To find this point, we unfasten the upper strap but keep the brace closed in its maximum position, asking the patient to fully inhale while carefully releasing the strap, finally fastening it in the minimum position, where the patient can achieve full inspiration with practically no resistance from the brace. At this time, we leave the patient in the lying position for a couple of minutes and move away to observe his/her reactions. Then, we help the patient get up and observe how she/he stands at the very first moment. The patient should be able to stand balanced in the frontal as well as in the sagittal plane for a couple of minutes if the brace is well designed. During this time, the orthotist can mark the trim lines, taking care not to cut more than necessary. It is better to cut too little than too much in this first trial. The brace is then prepared for a second trial and the procedure is repeated. In the second trial, the patient is usually able to stay in the upright position longer so the orthotist can spend more time deciding on the position of the final trim lines in both the upright and sitting positions. The orthotist and MD then consider whether the combined pressure points bring the patient into the maximum possible correction in all three planes. Pads should be touching the body at the right points and in the right direction according to the aforementioned principles. One of the most controversial issues is how high the brace should be on the convex thoracic side. There is no clear rule but, generally speaking, in flexible scoliosis the brace can be cut lower, slightly cranial to the apical level of the thoracic curve (warming the plastic and releasing pressure up to the apex, but maintaining some plastic to prevent the brace from being too short when the patient grows). In rigid curves, it is better to leave more plastic cranially. In this case, the maximum pressure goes to the more prominent part of the rib hump and, as long as the curve is not immediately corrected, the upper plastic will not push the ribs, so there is no need to warm and release pressure there. Do not cut the plastic because the plastic will not be visible, and it is always better to have additional plastic cranially to the apex to continue reaching the apex after the patient grows. The counter-pressure point working at the proximal thoracic region has to be totally adapted to the upper ribs, pushing to medial but with a very light dorsal direction to form a pair-of-force for deflection with the main thoracic pad. It should not be possible to bring the patient passively with our hands or actively by asking him/her to bend to the convex thoracic side to a bigger correction. The patient has to be blocked in the maximum possible correction in the frontal plane through these two forces provided by the main thoracic pad and the upper thoracic counter pad. If this correction is not achieved, consideration should be given to increasing the pressure by adding internal pads or cutting the upper counter pad to bring it to a more medial position by using metal extensors (Fig. 58). Sometimes, especially in very flexible curves, the correction is poor enough to consider re-making the brace as the best and most practical option.

The brace action also must be checked at the lumbo-pelvic region. Lumbar support in A and C types has to push on the lumbar convexity from below the apex and just reaching the apex, with no need to go over the apex. This support is, in a way, integrated into the pelvis section. The difference between the lumbar support and the real lumbar pad from B types has been explained above. In B types, it is necessary to check that the pelvis is brought fully to the left (in the example of a right thoracic/left lumbar scoliosis) and maintained by the brace in the frontal plane of reference (with 0° of axial rotation). Cranially, the lumbar region has to be derotated and translated to the right, reaching the maximum possible correction. The anterior design of the brace is essential to achieve this effect. The lumbar pad, which pushes from dorso-lateral to ventro-medial, needs an “escaping” space exactly at the same level in the anterior-lateral part. The body will totally fill this space in the upright position, and abdominal expansion during breathing mechanics will occur mostly laterally and back on the lumbar concavity. The anterior abdominal pad on the right side pushes from ventro-lateral to dorso-medial, exactly in the opposite direction of the lumbar pad, but at a lower level, to prevent any compression or “sandwich effect.” Otherwise, the best correction cannot be reached because the translation between the pelvis and lumbar regions is blocked. The anterior abdominal pad, which works in combination with the lateral pelvic pad, should help to stabilize the pelvis into the best possible 3D correction.

The brace is then finished by the orthotist and MD, who indicate a schedule for adaptation after training the patient/parents to put the brace on and take it off by herself/himself.

#### Protocols and everyday usage

We currently recommend the following three schedule alternatives:

1. Full-time: the patient must wear the brace 20–23 h per day
2. Part-time: the patient must wear the brace 14–16 h per day
3. Nighttime: the patient wears the brace only at night

Full-time is the most common recommendation for patients with a scoliotic curve over 25° and in the rapid period of growth at Tanner 2–3, Risser 0–2, pre-menarche, or 1 year after menarche (in girls).

Two different schedules for adaptation are indicated to reach full-time, but this aspect is flexible and many variations are made according to individual characteristics. In general, we recommend that the patient first sleep in the brace, then adapt to daytime wearing at home, and finally wearing the brace outside the home.

Historically, one to five trials has been necessary to sleep a full night in the brace. When the patient can wear the brace the entire first night, we recommend a rushed adaptation schedule. When the patient needs more than one night, we recommend a slower adaptation schedule. In the rushed schedule, after the first night, the patient wears the brace at home for 1 h the first day, then doubling the time each day until fully filling all the hours at home. Then she/he can go to the school wearing the brace, reaching full-time in a few days. In the slower schedule, the patient repeats each step one or two times (e.g., wearing the brace for 2 days only at night, then wearing the brace 1 h for 2 days at home, then 2 h for 2 days, 4 h for 2 days, and so on). It can be even slower, repeating daytime during 3 or 4 days. Basically, the schedule depends on the scoliosis rigidity.

One month after reaching full-time, the in-brace correction is checked in a radiograph. To reduce the number of radiographs as much as possible, we check in-brace correction in the AP/PA radiograph, while the sagittal plane alignment is followed out of brace by surface topography.

The brace progress is checked every 3 months during the period of rapid growth, especially before menarche. Clinical control is made every 6 months by measuring anthropometrics, ATI, breathing function, surface topography, clinical photos, self-perception, and HRQL (in our protocol TAPS and SRS-22). We do not repeat a new radiograph every 6 months when patients/parents report good compliance and the clinical picture has improved. No clinical changes or worsening or suspicion of a change in the curve pattern is considered a reason to repeat a radiograph out of brace. At this point, it is not necessary for the patient to remove the brace many hours before the radiograph is taken; 2–4 h is enough. Failure of bracing or a change in the curve pattern necessitates the development of a new strategy.

The life of the brace, on average, is around 1 year during the period between 1 year pre-menarche and half a year post-menarche. One year and a half/2 years before this period, and even longer when the brace is made 6 months after menarche.

When the brace becomes too small due to growth and development, a new brace is indicated when necessary and a new in-brace radiograph is prescribed to check correction. It is not rare to find a loss of correction into the second or third brace, but in our experience the out-of-brace value of the Cobb angle is not far from the in-brace value in those cases. Good responders use to show continued improvement of correction.

The full-time regime is followed by most patients until 2 years after menarche and Risser sign 3 (European)/4 (American). The patient is then recommended to wear the brace part-time. An out-of-brace radiograph is prescribed after 1 month of part-time wear (8 h out of the brace before the radiograph). When values are acceptable, part-time is maintained for 3 to 6 months and the patient then wears the brace only at night for 1 year. Out-of-brace radiographs are repeated 1 month after wearing the brace only at night, as well as 1 month and 1 year after weaning.

Outside of the weaning period, part-time wear is indicated for patients who will not wear the brace outside of the home. These patients are informed about the dose-effect response of bracing and are made aware of the risks for failure.

Nighttime use was formerly recommended in pre-puberal cases with good-to-excellent in-brace correction and rapid improvement of the clinical values. Nighttime bracing often allows us to increase the brace correction, but we follow the same basic principles.

A radiograph every 6 months is recommended when the patient is under the partial or nighttime regime or for full-time non-compliant patients, unless clinical values improve in a relevant way.

#### Exercises

Most patients combine bracing with a regular regime of exercises according to the Barcelona Scoliosis Physical Therapy School (BSPTS), which basically follows Schroth’s principles [30, 33]. The patient removes the brace to perform her/his Schroth exercises. In fact, the principles of correction used by the Rigo-Chêneau-type brace come from the evolution of the Schroth principles established by the BSPTS. We do not use to prescribe specific exercises in-brace, but patients can practice physical activities with the brace. It is recommended to remove the brace to participate in-group sports to avoid injuring others (in case of competition).

## Discussion

In spite of its growing popularity, literature about the Chêneau-type brace is limited in comparison with other popular brace concepts. With the exception of some well-designed prospective studies, the methodology of most of the published series is low in quality. It is first necessary to provide some background on the efficacy of brace treatment, in general terms, and related to the initial in-brace correction as a predictive factor of the end result.

Weinstein et al. recently published the results of a multicenter study on the effects of TLSO bracing in adolescents with idiopathic scoliosis, enrolling both a randomized cohort and a preference cohort, concluding that bracing significantly decreased the progression of high-risk curves to the threshold for surgery [3]. The external evidence for bracing, when a TLSO brace is used, strongly supports its effectiveness. Thus, the question is not whether bracing works or not but how to achieve the best possible result in terms of preventing surgery as a main goal, preventing progression as a primary goal, and permanently decreasing the pre-treatment angle as a secondary goal, all while improving the trunk shape and back asymmetry with no significant deterioration of function and, generally speaking, health-related quality of life (HRQL). The Weinstein paper also corroborated, in this case, the highest methodological quality, the previously suspected strong brace dose-response relationship. Previous prospective studies had shown the relationship between the short-term in-brace correction and end result. For whatever reason, in-brace correction is not reported in the Weinstein paper so, unfortunately, this relationship has not been confirmed in this paper. Nevertheless, even admitting its low quality, the existing evidence cannot be ignored.

In one of the classical references on brace treatment published in 1980, Carr et al. suggested that an initial in-brace correction of more than 50% was a predictive factor for a significant and permanent final correction [34]. In this study, however, 133 patients were treated with a Milwaukee brace, and by 1980, it was already known that the Milwaukee brace rarely achieved such a high in-brace correction on a regular basis. In a short series of 62 patients treated with the Milwaukee brace, Heine and Gotze [35] showed a very poor in-brace correction of less than 10%. In-brace correction as a predictor of the end result was also supported in the study from Noonan et al. [36], where patients treated with a Milwaukee brace and a progressing curve that required surgery showed a very poor in-brace correction of 8%, while those not needing surgery were initially corrected by the brace with a mean percentage of 20. Surprisingly, in this last series, a good result could still be expected with a poor in-brace correction based on today’s standards.

Later, three papers have stressed the relationship between initial in-brace correction and final outcome. Katz et al. [37] investigated the factors that could be predictive of the final outcome in patients with large curves treated with the Boston brace. The analyzed factors were Cobb angles, vertebral tilt angles, coronal decompensation, apical vertebral translation, apical vertebral rotation, lateral trunk shift, rib vertebral angle difference, pelvic tilt, and lumbar-pelvic relationship. Katz et al. concluded that patients with a double curve pattern in which the thoracic curve is over 35° Cobb and the lumbar-pelvic relationship is higher than 12° were significantly more likely to show curve progression. They also found that in-brace correction of at least 25% in double curves significantly increased the likelihood of success. Landauer et al. [38] predicted a final average curve correction of 7° in a child at growth when an in-brace correction of 40% could be reached with a Chêneau-type brace. Finally, Castro [39] concluded that brace treatment was not recommended in patients whose curves did not correct at least 20% in a TLSO. Most of the papers on braces that can be classified into the TLSO group have reported historically higher in-brace corrections in comparison with the Milwaukee brace, including the Chêneau-type brace.

The Boston brace has been considered the gold standard of the so-called TLSOs. It is definitely the most popular among scoliosis specialists around the world. Thus, it is obligatory for the authors of this paper to justify their gradual withdrawal from the Boston concept in favor of the Chêneau concept. Early studies on the Boston brace have reported about in-brace corrections of 50 to 60% [40]. Later, Uden et al. compared the in-brace correction of the Boston thoracic brace without superstructure (41%) with the Milwaukee brace (10%) [41]. In its already classical paper, Emans et al. also published a “mean better in-brace correction” of 51% [42]. The results of this last study showed that the Boston brace produced better in-brace corrections in single curves with the apex lower than T8, something also observed in other TLSOs. McCollough et al., reporting on the outcomes of the Miami brace, found that the initial correction was 36% in thoracic curves, 56% in thoracolumbar, and 63% in lumbar [43]. Double major curves showed an initial correction of around 37–38% for both curves, lumbar and proximal. At that time, popular opinion indicated that the Milwaukee brace was the choice for thoracic scoliosis with the apex at T8 or higher as well as for double curves with the thoracic apex cranial to T9. Conversely, a preliminary study from Laurnen et al. [44] showed the higher efficacy of the Boston brace compared with the Milwaukee, even for thoracic scoliosis with the apex at T8 and T7. The authors of this study strongly recommended locating the main thoracic pad pushing on the ribs from above but reaching the apex, in combination with a counter pad extended more cranially to the upper ribs on the concave thoracic side. Jonasson-Rajala et al. [45] and, later, Périé D et al. [46] also reported on the importance of the upper extension in order to create a three-point system to more efficiently correct the scoliosis at the main thoracic region. Also in the old study from Emans et al., the result for scoliosis with the apex lower than T7 was similar no matter if it was added to a superstructure or not [42]. The principle of “pushing at the apical level on the convexity of the main thoracic curve,” in combination with other forces, was also supported by Wynarsky and Schultz [47] and Aubin et al. [48, 49]. Thus, at least these two theoretical biomechanical principles, both present in the original Chêneau concept, eventually found full support in external evidence. However, sometimes theory goes one way and its practical application goes a different one. We still see many Boston braces fitted incorrectly in accordance with this principle, a fact that is clearly detrimental to the efficiency of the Boston concept. Unfortunately, we also see many Chêneau-type braces clearly failing on this principle.

The earliest results with the Chêneau-type brace were published in Germany. Hopf and Heine [50] report the outcomes of 52 patients treated with a Chêneau-type brace between 1979 and 1980. The mean initial in-brace correction, including single thoracic, single lumbar, and combined curves, was 41%. Weiss and Deez-Kraus [51] reported an initial in-brace correction of 39% for the main thoracic curve and 58% for the lumbar curve. Rigo et al. [52] presented a preliminary mean in-brace correction of 34%. Finally, Liljenqvist et al. [53] achieved a mean in-brace correction of 36%. In a further study, Rigo et al. [54] showed a mean in-brace correction of 31% for the major angle and 26% for the secondary angle, and also reported an initial in-brace axial rotation correction of 22% for the major angle.

Comparing the in-brace correction of this series with those related to the Boston brace, a logical question would be: why continue using this theoretically more complex concept when the Boston concept offered an existing good-to-excellent in-brace correction with the added benefit of a theoretically better standard?

First, it would be a mistake to consider external evidence in only one sense—the in-brace correction of the Cobb angle—when the series are hardly comparable. In-brace correction depends on many factors; some related to the brace but others related to the patient. Flexibility is one of, if not the most important, factors. Weiss has discussed his experience with an 11-year-old girl treated with one of his versions of the Chêneau-type brace, the Chêneau light® brace [55]. With a Cobb angle of 38° at the start of treatment, she was over-corrected and, after 2 years, had a Cobb angle of 19° with part-time bracing (16 h), which was sufficient to halt further progression. Thus, theoretically, any significant difference when comparing in-brace correction from two different studies could be due to both brace quality and patient quality. The ideal way to make studies comparable would be to match age, gender, and initial Cobb angle as well as curve pattern distribution and flexibility in each determined curve pattern. Thus, in-brace correction as an indicator in comparing brace quality between two different or similar brace concepts should only be partially considered, unless the methodology is strictly comparable.

On the other hand, when considering the discussion above, a significant increase of the in-brace correction reported by the same team over two different periods of time could be considered a good indicator of improved brace quality during the “learning curve” process after transitioning from one brace concept to a different one. Maruyama reported the outcomes of a first series of patients treated with a Rigo-Chêneau brace. His in-brace correction was similar to the first series reported by other authors in their preliminary series [56]. However, the pioneers of the Chêneau brace concepts have gradually increased the percentage of in-brace correction, suggesting that the correction and subsequent improved end results could be due to enhanced brace quality gained from clinical experience. We recently compared the in-brace correction of our own handmade braces (positive moulds corrected personally by the main author MR) with those from a CAD CAM system producing braces from models included in a library of pre-corrected moulds [57]. In this study, a group of 27 patients (26 female) with a mean age of 11.8 years (±2.1), Risser sign of 0.2 (±0.6), and an initial Cobb angle in the major curve of 33° (±7.2), all with no previous treatment and treated with a handmade brace, was compared with a matched group of 41 patients (39 female) treated with the CAD CAM system. In-brace correction—53% in the handmade group and 52.6% in the CAD CAM group—was not significantly different for the major curve. However, 53% is significantly higher than our first in-brace correction of 34% reported in 1995. The in-brace correction achieved with the CAD CAM version has been independently reported as 43, 42, 48, and 37% for thoracic, lumbar, major, and minor curves, respectively, in a group of 147 patients; a sub-group of patients fulfilling the more restrictive SOSORT criteria reportedly achieved corrections of 54, 59, 61, and 52% for thoracic, lumbar, major, and minor curves, respectively [58]. Notwithstanding, as discussed previously, the Chêneau-type brace is not just an orthopedic product but also a brace concept that is permanently evolving in pursuit of the highest possible standard. Brace design can suffer relevant changes and still be respectful to the original concept and theoretical principles. We presented a study comparing two different designs to treat the A1 curve type [59]. The A1 curve type is characterized by a long thoracic curve extending into the lumbar region, with a low apex in the main thoracic region (T9–T11). The study concluded that an over-corrected translation between the pelvis, including the coupled low lumbar region, and the main thoracic region significantly increased the correction when compared to the previously used classical design. The classic brace was built with a fully closed pelvic section, while the modern brace leaves one side totally open. A comparison of the in-brace correction in two similar groups of patients diagnosed with this A1 curve pattern showed a highly significant increase in-brace correction treated with the modern design in comparison with the group treated with the classic design (76.6 versus 45.3%, *p* < .001).

With this perspective, the fact that our own reported initial in-brace correction was not reaching 50% did not force us to give up. The main reason that we changed from the standard concepts used in Spain (Milwaukee, Boston, and Lyon) to the Chêneau concept around 1989 was the observed correlation between the use of the standard braces and the thoracic and lumbar morphological and functional flat back syndrome. Knowledge about the 3D nature of idiopathic scoliosis and its application in scoliosis treatment became very popular among scoliosis surgeons at that time. Jean Dubousset, in his already classic lecture entitled “Importance of the three-dimensional concept in the treatment of scoliotic deformities” (at the Montreal International Symposium on 3D Scoliotic Deformities joined with the VIIth International Symposium on Spinal Deformity and Surface Topography), pointed out the cause-effect relationship between the use of the Milwaukee, Boston, and Lyon braces and flat back syndrome [12]. This relationship has been confirmed and reported primarily by populations treated with the Boston brace [48, 49, 60–62], but this undesirable effect is also produced by other brace concepts, including the Chêneau-type brace. However, according to Dubousset, the only braces in use at the beginning of the 1990s that had the potential to correct scoliosis in 3D were the 3D brace from Graf and Dauny and the Chêneau brace. All these combined arguments, external evidence, and preferences of some relevant clinicians reinforced our attraction to the Chêneau-type brace and forced us to gradually abandon other brace concepts. However, as discussed previously, the standard of the Chêneau-type brace is poor and, in spite of the claim made by the first promoters, the potential to correct in 3D has been studied rarely. Three-dimensional correction makes reference to (1) frontal plane component, the lateral curvature as measured by the Cobb angle; (2) transversal plane component, the axial rotation of the apical vertebrae, as measured by different methods; and (3) sagittal plane component, related to a highly variable amount of altered spinal geometries impossible to measure with a single angle, which “should be decreased or increased.” In other words, in a progressive scoliosis, the torsional phenomenon gradually increases the lateral translation in the frontal plane, with the consequent increase of the Cobb angle; it also gradually increases the axial rotation, no matter which angle might be measured. Thus, reducing those angles is a direct action of the brace correction that can be easily assessed. However, in the sagittal plane, there is no single angle in the lateral radiograph to be decreased or increased always in the same direction for all the cases, which could be used to show the capability of the brace to correct in this plane. Sagittal parameters can be individually assessed according to pelvic incidence; sagittal values will need to be decreased in some cases and increased in others but this should be taken in consideration when designing studies. When we talk about a correction of the flat back component, what are we talking about? Morphological as well as geometrical lordotization of the main thoracic spine most likely happens in most cases of thoracic scoliosis; however, in a variable way and, depending on the orientation of the “plane of maximum deformity,” it is only sometimes visible in the lateral radiograph. The “paradoxical kypho-scoliosis,” a hyper-rotated lordo-scoliosis with a paradoxical kyphotic geometry in the lateral radiograph, although most typically related to severe “early onset scoliosis,” is also observed in adolescent idiopathic scoliosis (AIS) with a relatively mild Cobb angle and a very low morphological lordotic component. Also this should be taken in consideration when designing studies.

Very few studies report on the in-brace correction of the axial rotation. We showed an initial in-brace correction of the axial rotation in the major curve of 22% [54]. Later, in the comparison study of two brace designs—classical and open pelvis—to treat A1 type, the percentage of correction or the axial rotation (Perdriolle) was 29% for the classical design and 59% for the new open pelvis design [59].

The general claim about the Chêneau-type brace correcting flat back syndrome is not adequately supported by external evidence of quality. Cahuzac JP et al. presented outcomes in 161 patients treated with a Chêneau-Toulouse-Münster (CTM) brace. In this study, 55% of patients were pre-pubertal at the initiation of treatment and the initial main Cobb angle of 27.5° was reduced to 22.5° at the end of treatment, with 70% of the patients stabilized or improved, and 30% showing some progression [63]. The sagittal angle between T4 and T12 decreased during the treatment and returned to the initial value at the end of treatment, concluding that the “thoracic lordosis” was temporary modified by the brace. However, these results are hardly interpretable according to the previous discussion related to the sagittal regional or sub-regional values. Other studies have shown also the tendency to reduce the kyphotic angle at the main thoracic region [64–66]. However, the Chêneau-type brace design used in these studies could be significantly different to the design described in this current paper. As mentioned previously, the pelvic section of the brace is not built in retroversion, as defined in old brace concepts and seen in some Chêneau versions, but maintains a physiological anterior inclination. The Milwaukee concept, as well as the first-generation Boston brace, was based on the popular principle of “obliteration of the sagittal postural curvatures to achieve a better correction of the pathological lateral deviation.” A better understanding of the 3D nature of idiopathic scoliosis proved this principle incorrect, as it has been associated, in many cases, with an undesirable secondary flat back effect, in both the lumbar and thoracic regions [67]. Pelvis retroversion was considered the first step in the application of this principle due to its delordosant effect on the lumbar spine. Abdominal ventral pressure to ensure the delordosant effect was also very popular among orthotists. Soon after the introduction of the first-generation Boston brace, Willner [60, 61] emphasized the importance of reduction of the lumbar lordosis in the correction of the lumbar scoliosis. J. Chêneau was very critical of this very popular principle, recommending from the very beginning against unselective abdominal pressure and pelvis retroversion, but many orthotists used it in the past and continue to use it when constructing their braces under the name of Chêneau. Later, in a study of the 3D immediate effect of the Boston brace on the scoliotic lumbar spine, Labelle et al. [62] showed that the brace produced a distraction of the lumbar spine similar to that produced by the Harrington instrumentation by correcting the frontal plane deformity at expenses of a significant reduction of the physiological lumbar lordosis. They were not able to demonstrate any significant effect on rotation of the apical vertebra or “detorsion.” Modern Boston brace has abandoned the principle of pelvis retroversion and delordosis but still uses the unselective abdominal pressure. We must admit that at the current state of the art of the Chêneau-type brace, the principle of constructing the pelvis section with a physiological anterior inclination of the pelvis and physiological lumbar sagittal profile with selective abdominal expansion-pressure is subjective but not based on objective assessment. Notwithstanding, the concept of physiological pelvis anterior inclination is not a general one but has a high individual variability. Pelvis indexes (pelvic incidence; sacral slope, and pelvic tilt), in relationship with the sagittal geometry of the spine, more or less recoverable in the brace depending on the lordotic morphological component, could be used as a guide to define the amount of inclination the pelvic section of the brace should have case by case. We are now developing on this issue but cannot offer any information yet aside of the already explained three versions according to a normal, high, or low individual pelvic incidence.

Thus, the question about whether using Chêneau principles in brace construction can prevent the flat back or not is still open. In a relatively old study, we analyzed the 3D geometry of the spine in a group of patients treated with the first version of the Chêneau-type brace [68] and, although a significant number of patients showed improved sagittal alignment during brace treatment, some patients had what could be considered deterioration of the sagittal profile. From this experience, some significant changes were introduced in the brace design to prevent deterioration and further clinical observation supported the idea that a well-designed brace can prevent the deterioration of the morphological lordotization of the thoracic spine; further studies are necessary to support this statement. A recent study from Lebel et al. [69], comparing 3D effect from classical TLSO and Chêneau-type brace, has shown that only the Chêneau-type brace is able to reduce rotation of the apical vertebra. Coronal and sagittal correction did not differ significantly when comparing both brace concepts. The authors used EOS technology for spinal 3D reconstruction, but again here, we have no idea about which type of sagittal design they applied to their Chêneau-type version.

Although the end results were reported in some of the old series [50], more recent series support the effectiveness of the Chêneau-type brace when similar standards are observed. In 2003, two papers from independent centers with similar protocols in conservative management combining the Chêneau-type brace and Schroth scoliosis-specific exercises showed comparable effectiveness in preventing surgery. In a retrospective study, which included 343 patients (females only) with a mean Cobb angle of 33.4° treated with a Chêneau-type brace between 1993 and 1996, Weiss et al. found that only 12% of all patients underwent surgery [70]. All the patients were at least 15 years of age at the time they were last investigated. In the second study, Rigo et al. retrospectively analyzed the outcome in patients treated with a Rigo-Chêneau-type brace [71]. The objective was to determine whether a center with an active policy of conservative management had a lower prevalence of surgery compared with a center that had a non-intervention policy. The study included 106 braced patients who were at least 15 years of age at last review. Ultimately, only 14% (in a worst case analysis of all the intents to treat, including non-compliance and considering lost patients as failures) of braced patients underwent spinal fusion, which was statistically significantly lower than the 28% reported by the center with the policy of non-intervention.

Later, Weiss HR et al. [72] compared two brace concepts: the Chêneau-type brace (at that time, according to Rigo-Chêneau principles) and the soft brace concept, SpineCor. They compared the survival rates of the two different brace concepts with respect to curve progression and duration of treatment during pubertal growth spurt in two cohorts of patients. All girls in the study were pre-menarchial with the first clinical signs of maturation (Tanner 1–3). Twelve girls with an initial mean Cobb angle of 21.3° were treated with the SpineCor, compared to 15 girls matched in age with an initial mean Cobb angle of 33.7°. During the pubertal growth spurt, most of the patients (11 out of 12) with the SpineCor progressed clinically and radiologically. Progression was halted after the patients transitioned from the SpineCor to the Chêneau-type brace in seven of the progressive cases. The sample treated initially with the Chêneau-type brace showed no progression. After 24 months of treatment time, 73% of the patients with a Chêneau-type brace and 33% of the patients with the SpineCor were still under treatment with their original brace concepts. After 42 months of treatment, 80% of the patients with the Chêneau-type brace and 8% of the patients with the SpineCor survived with respect to curvature progression.

Cinnella et al. [73] presented at the SOSORT meeting in Lyon a retrospective series of 152 patients treated with a Chêneau-type brace, with a minimum of 20 months of follow-up (mean 56 months). At the end of treatment, the authors observed an average initial curve improvement of 23.3%. At follow-up, they observed an average improvement of 15% from the beginning of treatment. In this study, however, the protocol was different to further published series because 79% of the population was previously treated with a cast. Thus, we are not adding this to the rest of the series discussed in the paper.

Zaborovska-Sapeta et al. [74] conducted a prospective observational study according to SOSORT and SRS recommendations. The study included 79 patients with initial Cobb angles between 20° and 45°, no previous treatment, Risser 4 or higher at final evaluation, and a minimum 1-year follow-up after weaning from the brace. Results showed that 25% of all patients improved, 23% were stable, 39% progressed below 50°, and 13% progressed beyond 50°. This study suggested that conservative treatment with the Chêneau-type brace and physiotherapy (again, with similar brace standards and similar treatment approaches) can change the natural history of scoliosis, as 48% of patients did not progress.

Another retrospective cohort study from Ovadia et al. [75] was preformed to identify factors that could predict the therapeutic success or failure of the Rigo-Chêneau brace. Ninety-three patients with an average age of 13 years, Cobb angle of 32°, and Risser 1 were followed. All patients were treated with a Rigo-Chêneau-type brace during a mean treatment period of 36 months, and all had a 2-year follow-up after the termination of brace treatment. The authors concluded that the treatment was successful in 84% of patients, which indicates that the brace provides excellent clinical results in the treatment of mild to moderate AIS. Patients also showed a significant reduction of the angle of trunk rotation, suggesting the ability of the brace to correct the 3D trunk deformity, confirming initial observations about clinical improvement [68, 76]. Correction of the 3D trunk deformity can be assessed by using surface topography [68] as well as radiological indexes like the rib index from Grivas [77], although this last has not been used yet in patients treated with a Chêneau-type brace.

More recently, Rivett et al. [78] analyzed the effect of compliance to a Rigo System Chêneau brace and a specific exercise program on idiopathic scoliosis curvature, and compared the quality of life (QoL) and psychological traits of compliant and non-compliant subjects. Fifty-one subjects, all girls aged 12–16, with Cobb angles 20–50° participated in the study. Subjects were divided into two groups, according to their compliance, at the end of the study. The compliant group wore the brace 20 or more hours a day and exercised three or more times per week. The non-compliant group wore the brace less than 20 h a day and exercised less than three times per week. Cobb angle, vertebral rotation, Scoliometer reading, peak flow, QoL, and personality traits were compared between groups. The compliant group wore the brace 21.5 h per day and exercised four times a week, and significantly improved in all the measures compared to the non-compliant subjects, who wore the brace 12 h per day, exercised 1.7 times per week and significantly deteriorated (*p* < .0001). The major Cobb angle in the compliant group improved 10.19° (±5.5) and deteriorated 5.52° (±4.3) in the non-compliant group. Compliant group had a significantly better QoL than the non-compliant subjects. The compliant subjects were significantly more emotionally mature, stable, and realistic than the non-compliant group (*p* < .05). The conclusion of this study was that good compliance of the RSC brace and a specific exercise regime resulted in a significant improvement in curvature, while poor compliance resulted in progression. A poorer QoL in the non-compliant group possibly was caused by personality traits of the group, being more emotionally immature and unstable. Other aspects of QoL like function have not been studied. Based on the theoretical principles of this brace, users claim about no deterioration of breathing function, but we do not know any study supporting this.

As discussed previously, different brace concepts cannot fairly be compared to current state-of-the-art concepts. Thus, the old statement about the best brace being managed with the highest experience by a particular multidisciplinary team could still be defended, at least in terms of patient safety. However, once the efficacy of bracing has been strongly supported, further studies are necessary to demonstrate the ability of a particular brace concept to correct scoliosis in 3D and whether or not 3D in-brace correction is a factor when predicting the success of bracing.

The theoretical disadvantage of the Chêneau-type concept of bracing is the complexity of its principles and fabrication, which has been associated with poor standards. By summarizing previously reported studies and providing supporting published data, this paper attempts to raise awareness and education to improve the future standard of the Chêneau concept. Nevertheless, the fact that consistent and comparable results are reported by at least five independent centers using similar standards cannot be ignored.

### Case reports

#### Case reports 1

**Fig. 52:**
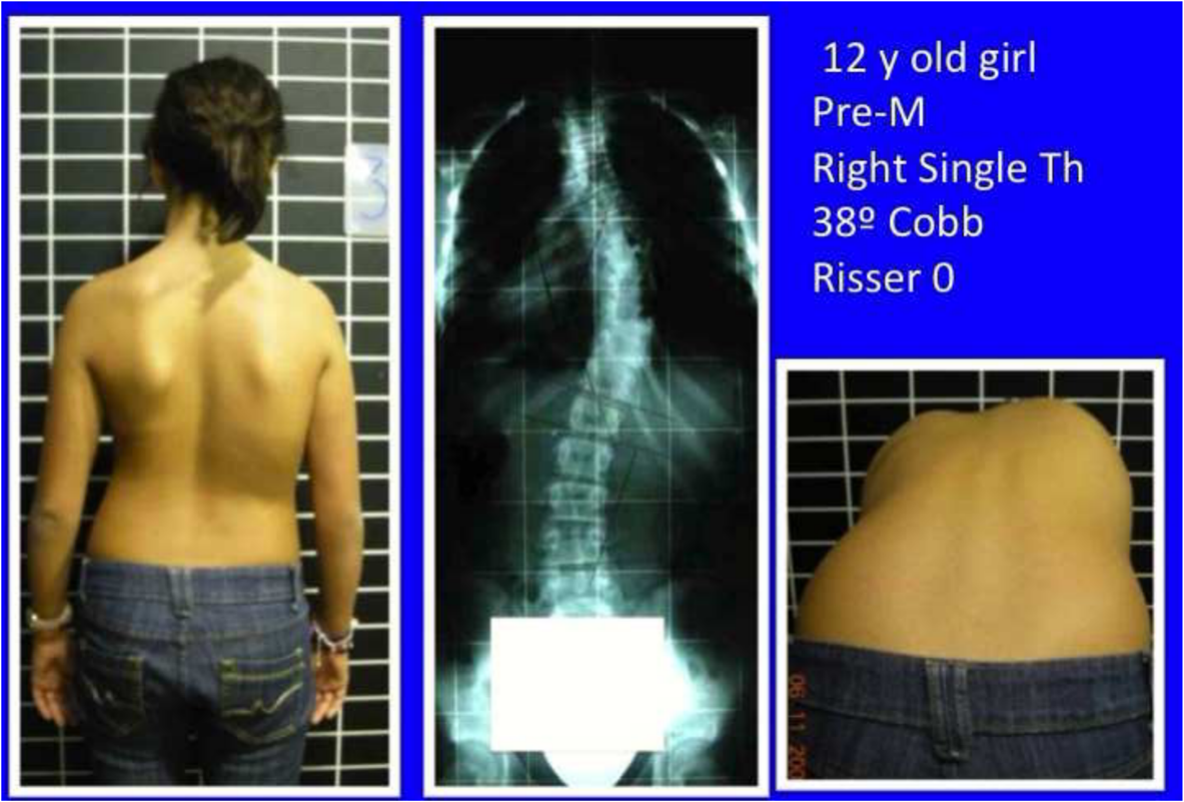
Case reports 1. A 12-year-old girl with a right thoracic scoliosis measuring 38° at Risser 0 and pre-menarche. She had potential to progress to 55° or more at Risser 5

**Fig. 53:**
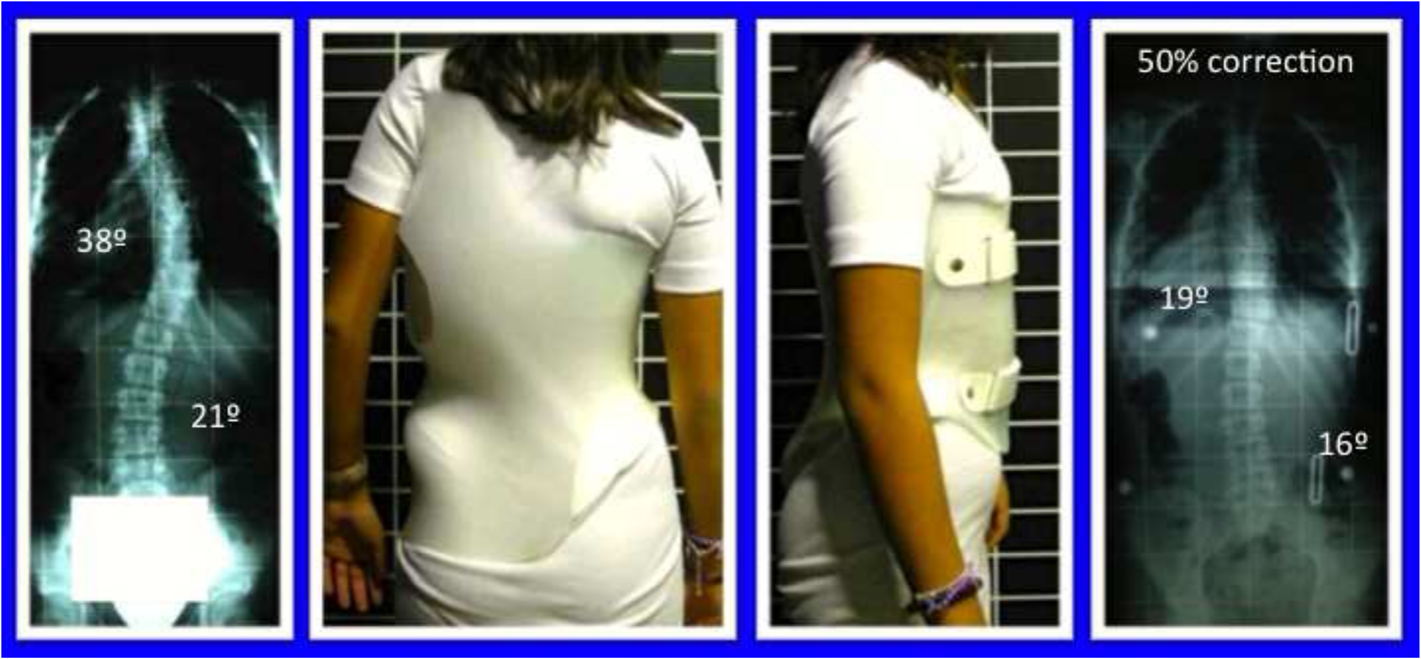
Case reports 1. Excellent in-brace correction (50%) with an A1-type brace

**Fig. 54:**
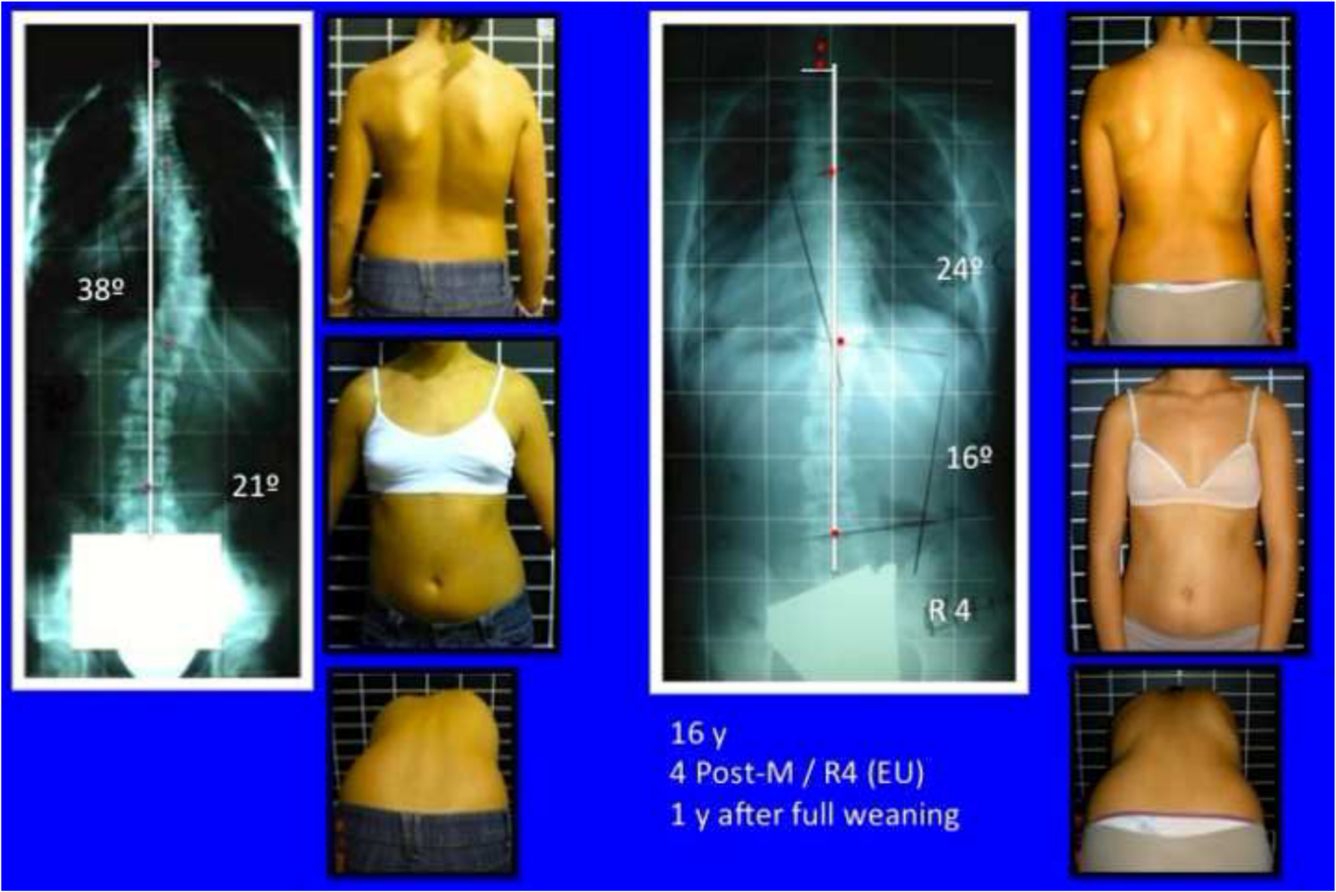
Case reports 1. End result 1 year after full weaning. The girl had a stable curve of 24° (initial curve 38°) at Risser 4 (16 years of age). Clinical improvement is also noticeable

This reports the final radiological and clinical result in a 12-year-old girl diagnosed with adolescent idiopathic scoliosis, 1 year after finishing treatment. She was still pre-menarche at diagnosis and initiation of treatment. Clinical presentation was like 3C and a radiograph confirmed a right long-low thoracic scoliosis measuring 38° Cobb and rotation degree II^1/2^ according to Nash and Moe, and Risser 0. Curve pattern and “transitional point offset” were compatible with A1 type. Initial clinical and radiological presentation is shown in Fig. 52. Being closed to 40° Cobb before reaching Risser 1, the potential for progression of a thoracic scoliosis is reaching Risser 5 with a scoliosis over 55° [79]. Based on prognosis she was recommended to go under full-time bracing and PSSE according to BSPTS-Schroth principles. Figure 53 shows the girl just after first fitting, once the brace was finished, and before adaptation. The same figure shows also the first radiograph in brace with an excellent correction of 50% of the Cobb angle and a mild but relevant correction of the rotational component. She wore the brace full-time for 2 years and one additional year only at night. Figure 54 shows her radiograph 1 year after full weaning, at 16 years of age, Risser 4 (European scale), 4 years after menarche. A well-balanced scoliosis measuring 24° thoracic and 16° lumbar, with no structural proximal curve and just a mild imbalance at T1 was confirmed. Rib cage appeared perfectly balanced on the pelvis. She had her first menstruation few months after starting treatment, still at 12 years of age. Clinical result is also noticeable when looking at both back asymmetry and postural balance in the frontal plane. Angle of trunk inclination (ATI) measured in forward bending from sitting position came down from 14° to 7° (50% correction). Sagittal photos were not documented but a photo from dorsal does not show any deterioration of the morphological flat back. She continued practicing her exercises during the year after full weaning.

#### Case reports 2

**Fig. 55:**
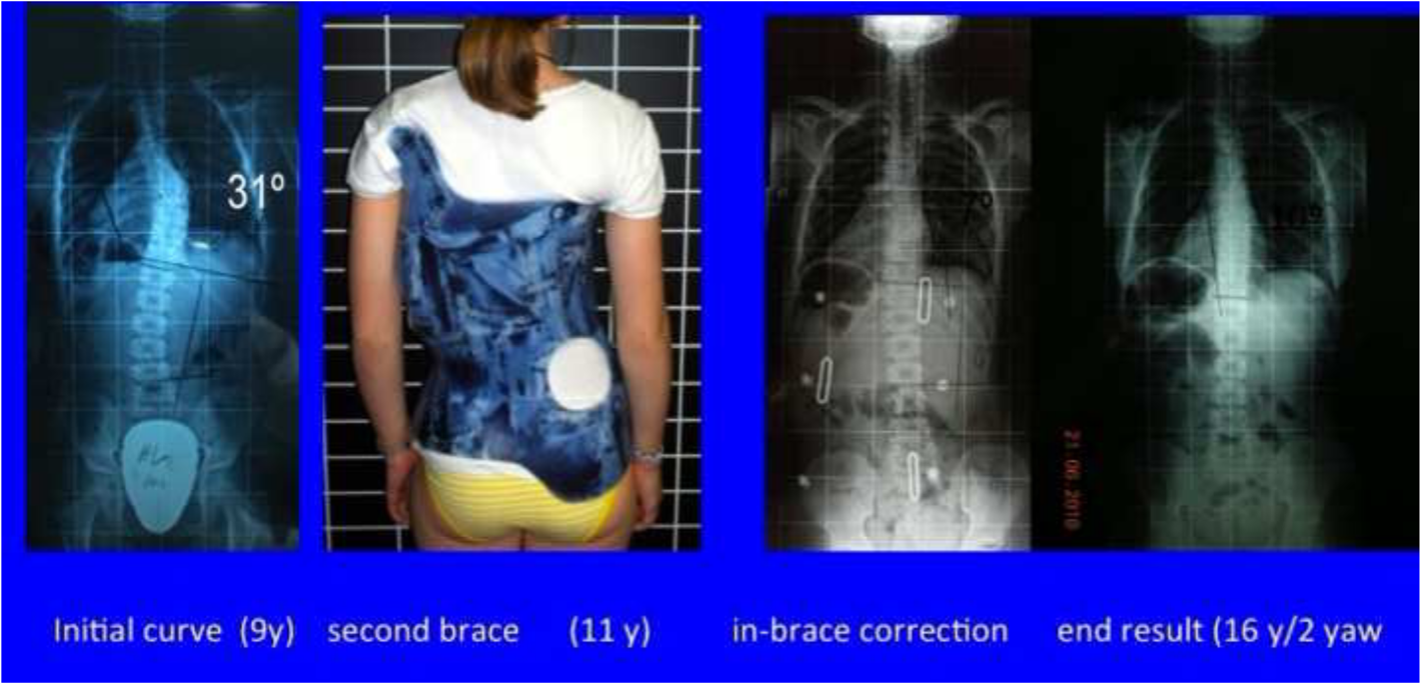
Case reports 2. A 9-year-old girl showing an excellent end result 2 years after full weaning. She was treated with a C1-type brace and PSSE

**Fig. 56:**
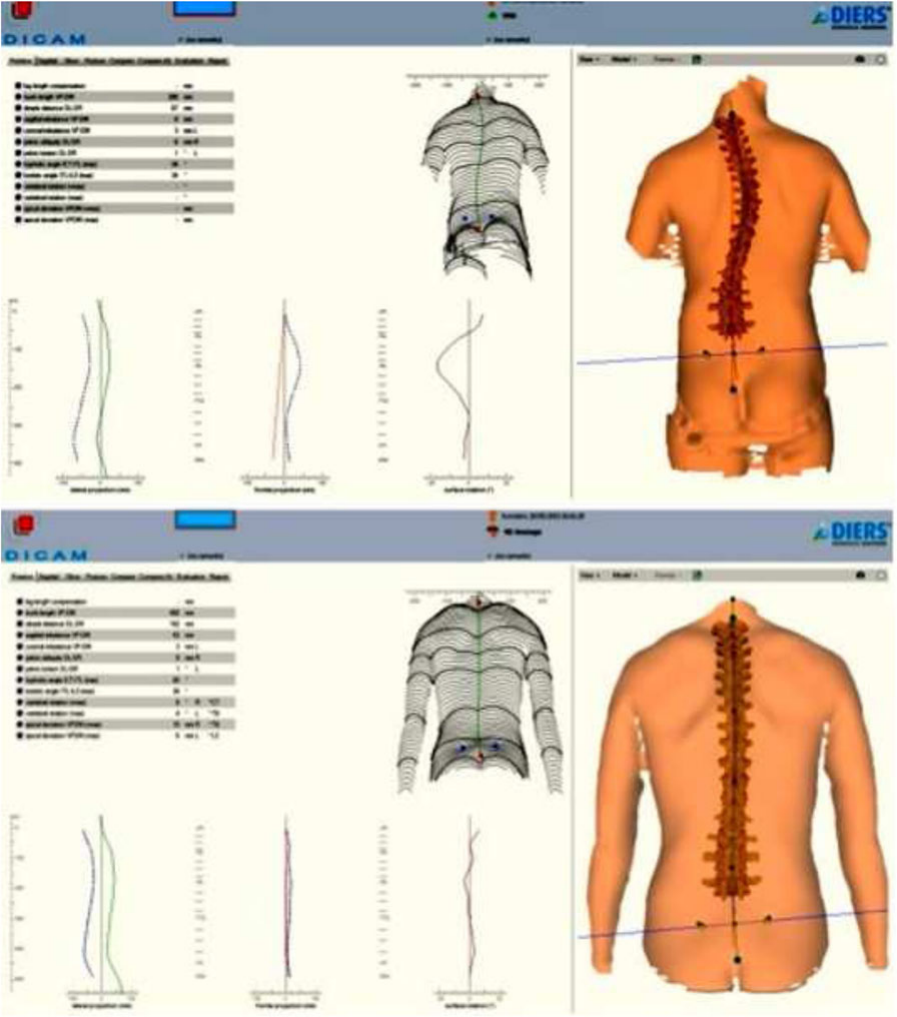
Case reports 2. Clinical result (Formetric) of the girl still noticeable at 20 years of age, 6 years after full weaning

This reports the final radiological result in a 9-year-old girl diagnosed with late-onset juvenile idiopathic scoliosis. She had a clinical presentation like 3C (A2 type) and radiograph showed a single right thoracic scoliosis measuring 31° Cobb. We made a first C1-type brace (at that time still with pelvis closed) with an in-brace correction of around 50% (in-brace Cobb angle was 15°). Prescription was “full-time.” She was also recommended to start physiotherapy scoliosis-specific exercises (PSSE) according to Barcelona School (BSPTS), based on Schroth principles. After 1 year of treatment, she was fitted in a second brace due to growth and development showing an in-brace Cobb angle of 7°. The girl was then 11 years old at Risser 0 and had pre-menarche status. She continued wearing the brace full-time. Her menarche was at 12 years of age. She continued wearing the brace full-time (>20 h) until 13 years of age, and due to an excellent clinical result, with a total reduction of the ATI from 9° to 0°, she was recommended to go under “nighttime” regimen. Total weaning was at 14 years of age. A final radiograph was ordered when she was 16 years of age, 2 years after weaning, showing a very mild right thoracic curve measuring 10° Cobb, at Risser 5. Figure 55 shows the radiological result. Figure 56 shows the esthetic improvement 4 years after her last radiograph, at 20 years of age. Due to the consistent clinical improvement, no radiograph was then prescribed. Her spine looked rectilinear with insignificant right/left asymmetries and a relatively preserved sagittal profile, with a mild lumbar hypo-lordosis and thoracic hypo-kyphosis, but still into the normal range for the Formetric values, with no clinically relevant change in comparison with the initial profile, if perhaps a very mild ventral imbalance.

#### Case reports 3

**Fig. 57:**
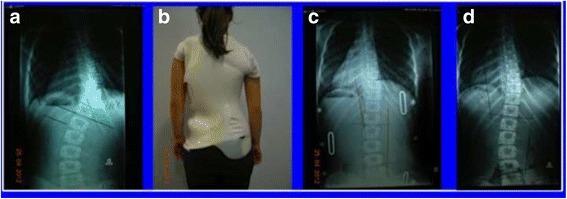
Case reports 3. A 9-years-old girl (menarche at 10 years of age) treated with an A2-type brace (**b**) after being diagnosed with a right convex thoracic scoliosis measuring 44º Cobb (**a**). After excellent in-brace correction (**c**), she showed a short-term correction, out of brace (**d**), which allow us to recommend her for early weaning

**Fig. 58:**
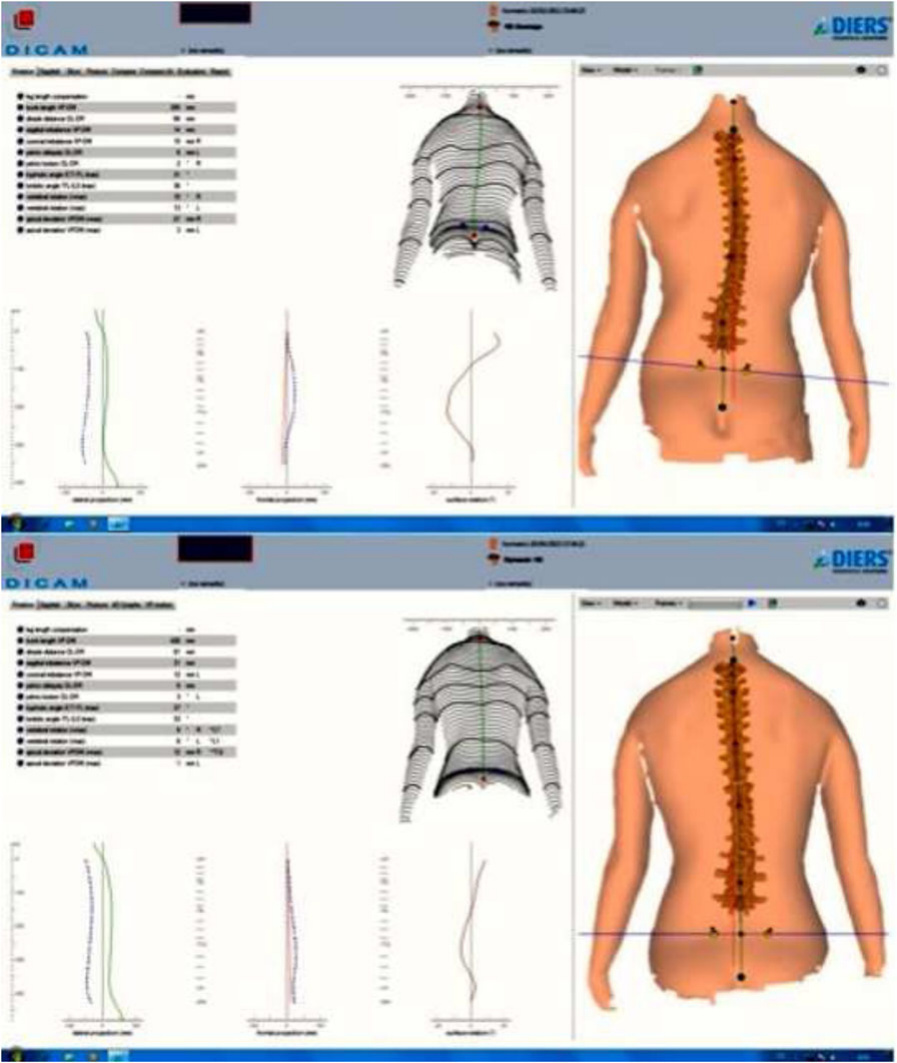
Case reports 3. Clinical improvement (Formetric®) after 3 years of treatment (1 year full-time + 2 years nighttime)

This reports the initial in-brace correction and short-term results in a 9-year-old girl initially diagnosed with juvenile idiopathic scoliosis. Although the initial Cobb angle was 44° and she was 9 years of age, she had menarche at 10 years of age, few months after coming for her first consultation, so her evolution and potential for progression was expected to be like AIS. She was recommended for bracing immediately and was fitted with an A2-type brace. The Cobb angle in-brace (1 month of adaptation after reaching full-time) was 13° (70% in-brace correction) and consequently was strongly recommend wearing the brace in a full-time regimen (>20 h). She practiced also PSSE according to BSPTS regularly and was controlled every 6 months. Due to an excellent clinical response with a total correction of the back asymmetry (Formetric ®) and a very significant reduction of the ATI, she was recommended for partial early weaning, going under a “nighttime regimen” after she showed a significant correction of the Cobb angle in an out-brace radiograph, taken after 1 week using the brace only at night, and 8 h after she took of the brace in the morning. The radiograph showed a Cobb angle of 16°. Two years later she still showed a highly consistent improvement of the back asymmetry with a practical inversion of the expected asymmetry for a right thoracic scoliosis/left lumbar. She was then recommended to stop bracing. Her last radiograph, 3 months after total weaning, shows a quasi-stable situation with a scoliosis still under 20°. Final result will be assessed in a new radiograph 2 years after weaning in 2017. Figure 57 shows the radiological results. Figure 58 shows the clinical changes by surface topography. The asymmetry remained inverted after 2 years of nighttime brace (and just 1 year of full-time bracing). Sagittal profile shows a relatively disharmonic configuration with lower lumbar lordosis/upper thoracic kyphosis and a hypo-kyphotic/hypo-lordotic configuration in the main thoracic and upper lumbar region, but not different to the initial profile. The girl presents a mild sagittal imbalance, but she is doing well in terms of QOL.

#### Case reports 4

**Fig. 59:**
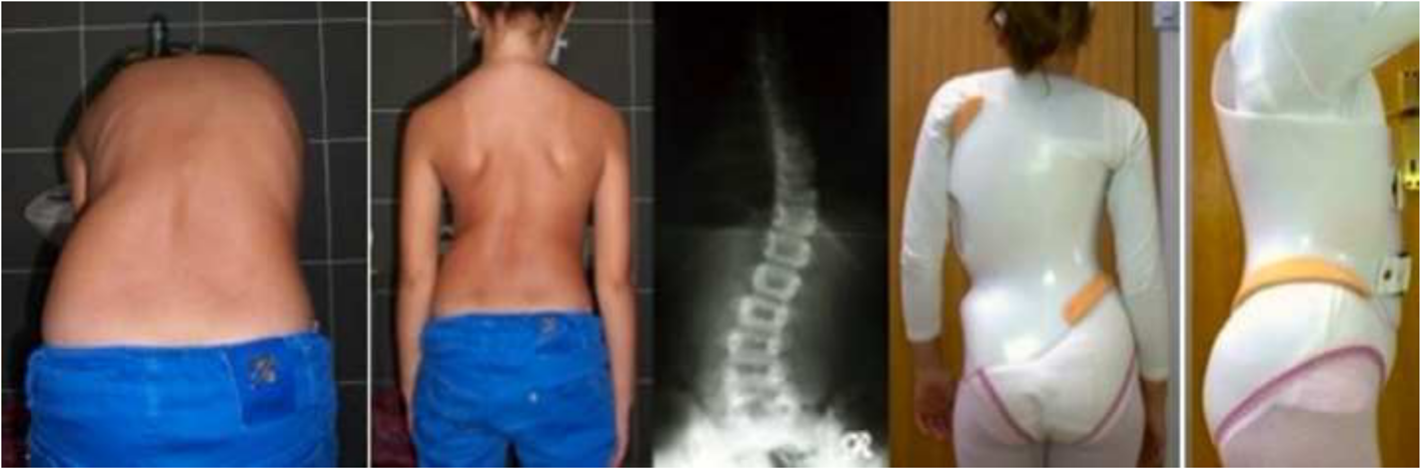
Case reports 4. An 8-year-old girl treated initially with an A2-type brace by the junior author MJ. One year after initiation of treatment she showed some clinical and radiological changes (Fig. 60). The girl is still under treatment

**Fig. 60:**
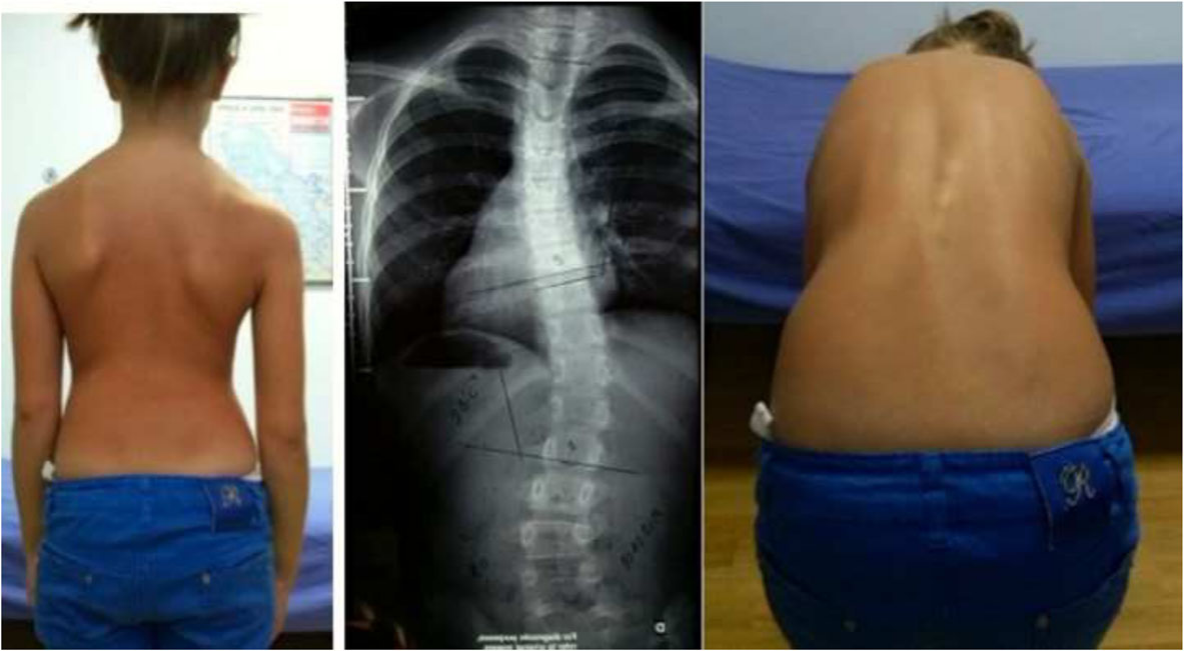
Case reports 4. One year after initiating treatment with an A1-type brace, this girl showed a change in the curve pattern, which was considered to be relevant for this brace concept. The long-low thoracic curve (apex T10) combined with a hidden proximal curve (A1 with D mod), changed to a high thoracolumbar curve (apex disc T11–T12 with aplasia of the 12 rib) combined with a high thoracic curve. She was treated then with a B2-type brace as shown in Fig. 61. The girl is still under treatment

**Fig. 61:**
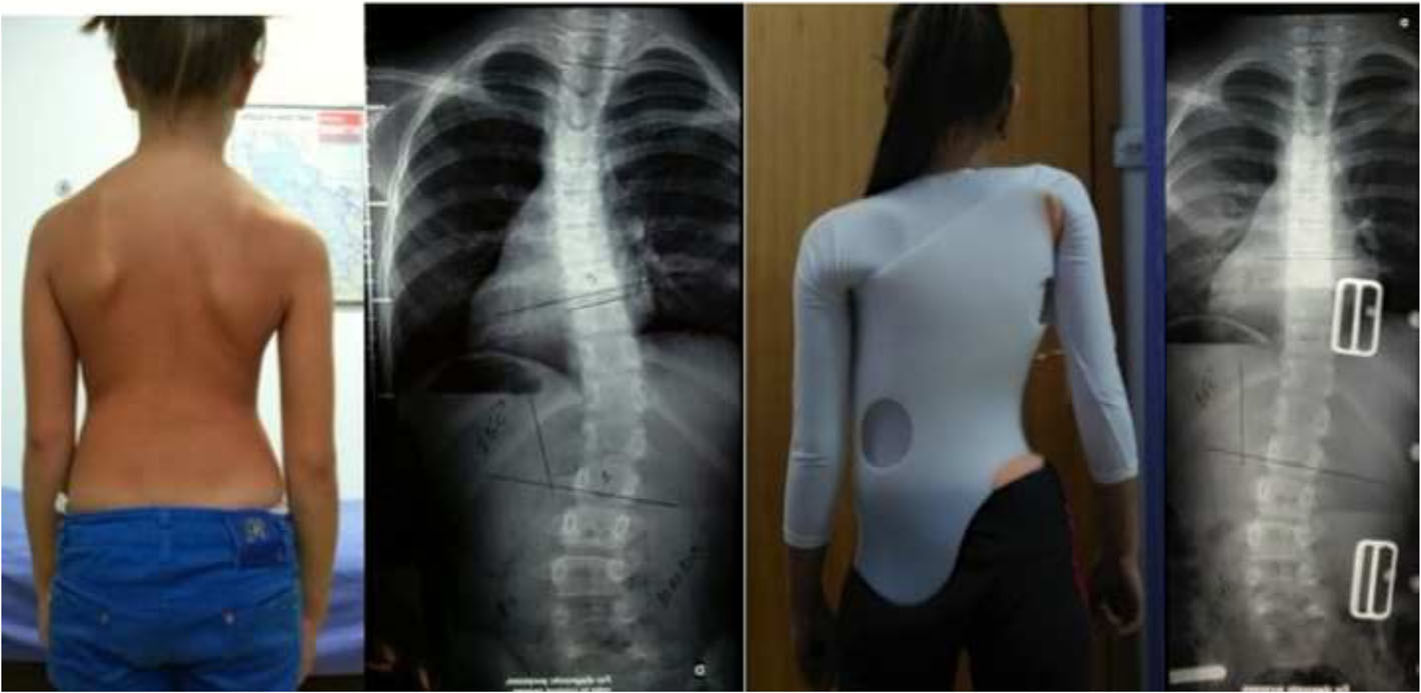
Case reports 4. Excellent in-brace correction with a B2-type brace in a girl who was previously treated with an A2-type brace, which was associated with a change in the curve pattern. The girl is still under treatment

**Fig. 62:**
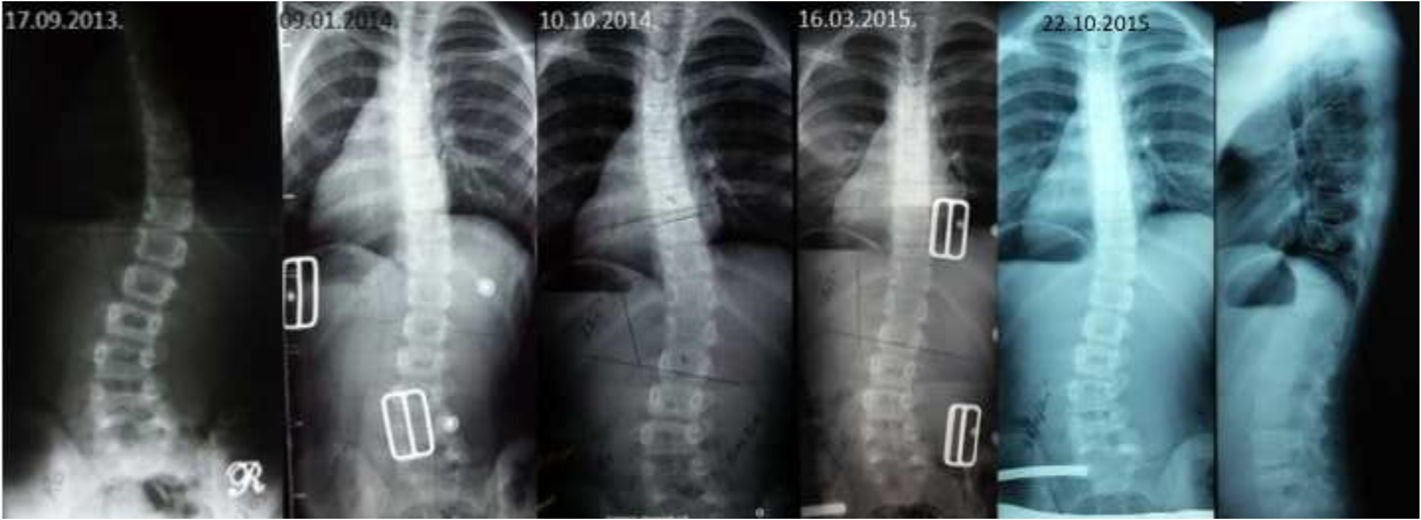
Case reports 4. Radiological evolution from the initial presentation on September 2013, in-brace correction (A1-type brace); 1 year later, out the brace; in-brace correction in her second brace (B2 type) and out the brace on October 2015, 2 years after initiation of treatment. The lateral projection shows a normal sagittal configuration. The girl is still under treatment

**Fig. 63:**
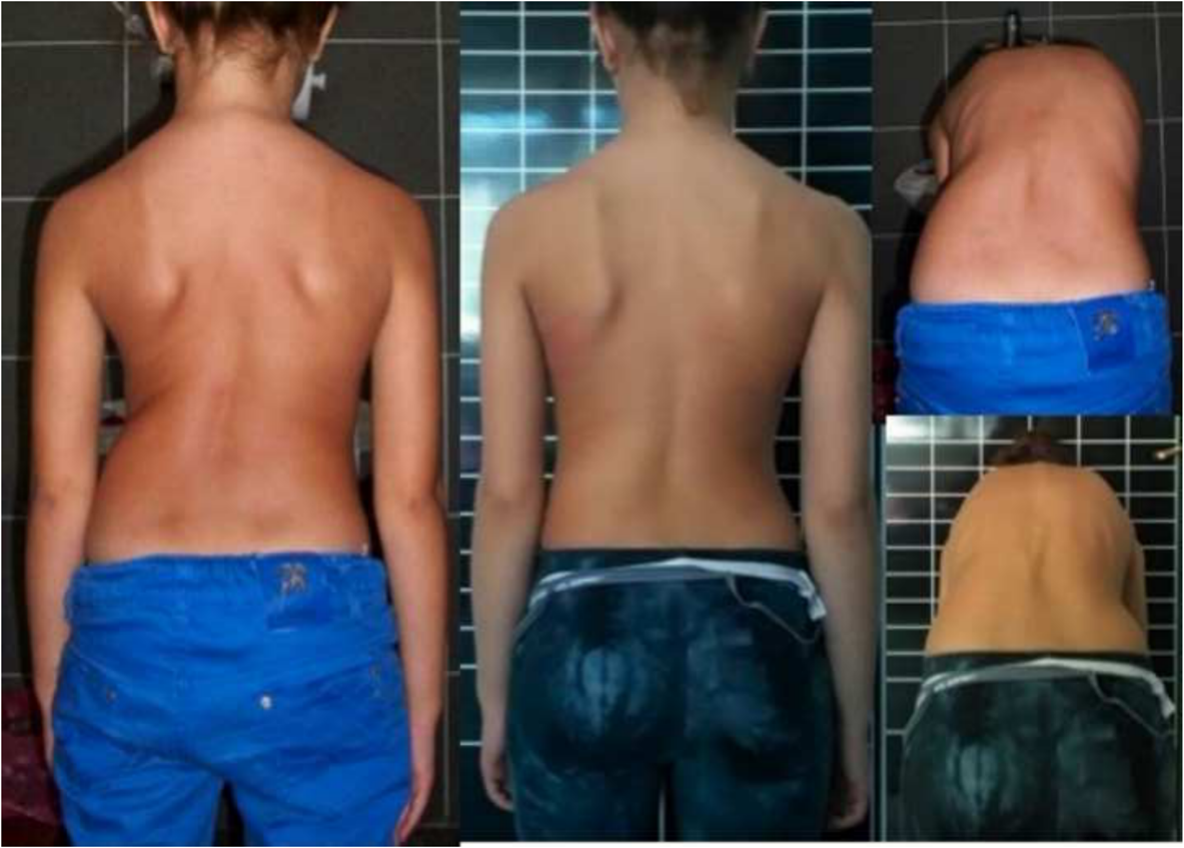
Case reports 4. Short-term clinical result, 2 years after initiation of the treatment, first with an A1-type brace and later with a B2-type brace. Brace had to be remade after a change in the curve pattern. The girl is still under treatment

This reports about a change in the curve pattern observed after 1 year of treatment with an A1-type brace, and the short-term radiological and clinical result in an 8-year-old girl treated by the junior author MJ. The girl was diagnosed with juvenile idiopathic scoliosis at 8 years of age. Clinical and radiological criteria were for A1 type: long-low thoracic curve combined with a hidden proximal thoracic curve (not visible on the radiograph due to bad quality, but clinically) (Fig. 59). She was then treated with an A1-type brace showing an excellent in-brace correction. One year later she had changed her curve pattern. The right long-low thoracic curve became a border case after a caudal migration of the apex to the disc T11–T12, combined with a high main thoracic rather than proximal thoracic curve (Fig. 60). We decided to change her brace design using a B2-type brace, which was made and adapted again by the second author MJ. The in-brace correction was also excellent in the B2-type brace ad clinical improvement after 1 year and half of treatment was also noticeable (Figs. 61, 62 and 63). The girl is still under treatment. The change in curve pattern is not a rare event in early onset scoliosis but in late onset, when using a hyper-corrective brace.

## Conclusions

The Chêneau-type brace according to Rigo principles and classification is a 3D corrective device able to provide excellent in-brace correction as well as radiological and cosmetic end results. This paper offers a vast description of the applied corrective principles as well as a short revision of the specific classification, with the ambitious objective of improving the observed poor standard of the classically called Chêneau brace.

## Abbreviations

3D: Tridimensional
BSPTS: Barcelona Scoliosis Physical Therapy School
BUFA: Bundesfaschule für Orthopadie Technik
CSL: Central sacral line
CTM: Chêneau-Toulouse-Münster
HRQL: Health-related quality of life
PSSE: Physiotherapy scoliosis-specific exercises
RASO: Relative anterior spinal overgrowth
SOSORT: International Society on Orthopaedic and Rehabilitation Treatment
TLS: Toracolumbosacral
TP: Transitional point

## References

[1] Maruyama T, Grivas TB, Kaspiris A (2011). Effectiveness and outcomes of brace treatment: a systematic review. Physiother Theory Pract.

[2] Negrini S, Minossi S, Bettany-Saltikov J, Zaina F, Chockalingam N, Grivas TB, Kotwicki T, Maruyama T, Romano M, Vasiliadis ES (2010). Braces for idiopathic scoliosis in adolescents. Spine.

[3] Weinstein SL, Dolan LA, Wright JG, Dobbs MB (2013). Effects of bracing in adolescent with idiopathic scoliosis. N Engl J Med.

[4] Rigo M, Negrini S, Weiss HR, Grivas TB, Maruyama T, Kotwicki T, the members of SOSORT (2006). SOSORT consensus paper on brace action: TLSO biomechanics of correction (investigating the rationale for force vector selection). Scoliosis.

[5] Negrini S, Aulisa AG, Aulisa L, Circo AB, de Mauroy JC, Durmala J, Grivas TB, Knott P, Kotwicki T, Maruyama T, Minozzi S, O’Brien JP, Papadopoulos D, Rigo M, Rivard CH, Romano M, Wynne JH, Villagrasa M, Weiss HR, Zaina F (2012). 2111 SOSORT guidelines: Orthopaedic and Rehabilitation treatment of idiopathic scoliosis during growth. Scoliosis.

[6] Chêneau J (1994). Corset-Chêneau. Manuel d’orthopédie des scolioses suivant la technique originale.

[7] Weiss HR, Rigo M, Chêneau J (2000). Praxis der Chêneau Korsettversorgung in der Skoliose-Therapie.

[8] Weiss HR, Rigo M (2008). The Chêneau concept of bracing – actual standards. Stud Health Technol Inform.

[9] Rigo M, Weiss HR (2008). The Chêneau concept of bracing – biomechanical aspects. Stud Health Technol Inform.

[10] Rigo M, Villagrasa M, Gallo D (2010). A specific scoliosis classification correlating with brace treatment: description and reliability. Scoliosis.

[11] Negrini S, Grivas TB (2010). Introduction to the “Scoliosis” Journal Brace Technology Thematic Series: increasing existing knowledge and promoting future developments. Scoliosis.

[12] Dubousset J (1992). Importance of the three-dimensional concept in the treatment of scoliosis deformities. Dansereau J ed. International Symposium on 3D Scoliotic Deformities joined with the VII International Symposium on Spinal Deformities and Surface Topography.

[13] Raso VJ, Russell GG, Hill DL, Moreau M, MCivor J (1991). Thoracic lordosis in idiopathic scoliosis. J Pediatric Orthop.

[14] Raso VJ, Lou E, Hill DL, Mahood JK, Moreau MJ, Durdle NG (1998). Trunk distorsion and idiopathic scoliosis. J Pediatric Orthop.

[15] Guo X, Chau WW, Chang YL, Cheng JC, Burwell RG, Dangerfield PH (2005). Relative anterior spinal overgrowth in adolescent idiopathic scoliosis – result of disproportionate endochondral-membranous bone growth? Summary of an electronic focus group debate of the IBSE. Eur Spine J.

[16] Burwell RG, Dangerfield PH (2006). Pathogenesis of progressive adolescent idiopathic scoliosis. Platelet activation and vascular biology in immature vertebrae: an alternative molecular hypothesis. Acta Orthop Belg.

[17] Zhu F, Qiu Y, Yeung HY, Lee KM, Cheng JC (2006). Histomorphometric study of the spinal growth plates in idiopathic scoliosis and congenital scoliosis. Pediatric Int.

[18] Burwell RG, Dangerfield PH, Freeman BJ (2008). Concepts on the pathogenesis of adolescent idiopathic scoliosis. Bone growth and mass, vertebral column, spinal cord, brain, skull, extra-spinal left-right skeletal length asymmetries, disproportions and molecular pathogenesis. Stud Health Technol Inform.

[19] Chu WC, Lam WM, Ng BK, Tze-Ping L, Lee KM, Guo X, Cheng JC, Burwell RG, Dangerfield PH, Jaspan T (2008). Relative shortening and functional tethering of the spinal cord in adolescent idiopathic scoliosis – Result of asynchronous neuro-osseous growth, summary of an electronic focus group debate of the IBSE. Scoliosis.

[20] Burwell RG, Aujla RK, Freeman BJ, Dangerfield PH, Cole AA, Kirby AS, Polak FJ, Pratt RK, Moulton A (2008). The posterior skeletal thorax: rib-vertebral angle and axial vertebral rotation asymmetries in adolescent idiopathic scoliosis. Stud Health Technol Inform.

[21] Gu SX, Wang CF, Zhao YC, Zhu XD, Li M (2009). Abnormal ossification as a cause the progression of adolescent idiopathic scoliosis. Med Hypotheses.

[22] Liu Z, Tam EM, Sun GQ, Lam TP, Zhu ZZ, Sun X, Lee KM, Ng TB, Qiu Y, Cheng JC, Yeung HY (2012). Abnormal leptin bioavailability in adolescent idiopathic scoliosis: an important new finding. Spine.

[23] Schlösser TP, van Stralen M, Brink RC, Chu WC, Lam TP, Vincken KL, Castelein RM, Cheng JC (2014). Three-dimensional characterization of torsion and asymmetry of the intervertebral disc versus vertebral bodies in adolescent idiopathic scoliosis. Spine.

[24] Stokes IA, Spence H, Aronsson DD, Kilmer N (1996). Mechanical modulation of vertebral body growth. Implications for scoliosis progression. Spine.

[25] Burwell RG (2003). Aetiology of idiopathic scoliosis: current concepts. Pediatr Rehabil.

[26] Murray DW, Bulstrode CJ (1996). The development of adolescent idiopathic scoliosis. Eur Spine J.

[27] Duval-Beaupere G, Taussig G, Mouilleseaux B, Pries P, Mounier C (1992). Prognostic factors for idiopathic scoliosis. Dansereau J ed. International Symposium on 3D Scoliotic Deformities joined with the VII International Symposium on Spinal Deformities and Surface Topography.

[28] Legaye J, Orban C (1995). Evolution of scoliosis by optical scanner I.S.I.S.. Stud Health Technol Inform.

[29] Bernhardt M, Bridwell KH (1989). Segmental analysis of the sagittal plane alignment of the normal thoracic and lumbar spines and thoracolumbar junction. Spine.

[30] Lehnert-Schroth C (2000). Dreidimensionale Skoliosebehandlung.

[31] Moe JH, Kettleson D (1970). Idiopathic scoliosis: Analysis of curve patterns and preliminary results of Milwaukee brace treatment in one hundred sixty-nine patients. J Bone Joint Surg Am.

[32] Working Group on 3-D Classification (Chair Larry Lenke, MD), and the Terminology Committee, March 2000. SRS Terminology Committee and Working Group on Spinal Classification Revised Glossary of Terms. http://www.srs.org/professionals/online-education-and-resources/glossary/revised-glossary-of-terms.

[33] Rigo M, Quera-Salvá G, Villagrasa M, Ferrer M, Casas A, Corbella C, Urrutia A, Martínez S, Puigdevall N (2008). Scoliosis intensive out-patient rehabilitation based on Schroth method. T.B. Grivas (Ed.) The Conservative Scoliosis Treatment. Stud Health Technol Inform.

[34] Carr WA, Moe JH, Winter RB, Lonstein JE (1980). Treatment of idiopathic scoliosis in the Milwaukee brace. J Bone Joint Surg Am.

[35] Heine J, Gotze HG (1985). Final results of the conservative treatment of scoliosis using the Milwaukee brace. Z Orthop Ihre Grenzgeb.

[36] Noonan KJ, Weinstein SL, Jacobson WC, Dolan LA (1996). Use of the Milwaukee brace for progressive idiopathic scoliosis. J Bone Joint Surg Am.

[37] Katz DE, Durrani AA (2001). Factors that influence outcome in bracing large curves in patients with adolescent idiopathic scoliosis. Spine.

[38] Landauer F, Wimmer C, Behensky H (2003). Estimating the final outcome of brace treatment for idiopathic thoracic scoliosis at 6-month follow-up. Pediatr Rehabil.

[39] Castro FP (2003). Adolescent idiopathic scoliosis, bracing and the Hueter-Volkmann principle. Spine J.

[40] Watts HG, Hall JE, Stanish W (1977). The Boston brace system for the treatment of low thoracic and lumbar scoliosis by use of a girdle without superstructure. Clin Orthop Relat Res.

[41] Uden A, Willner S, Peterson H (1982). Initial correction with the Boston thoracic brace. Acta Orthop Scand.

[42] Emans JB, Kaelin A, Bancel P, Hall JE, Miller ME (1986). The Boston bracing system for idiopathic scoliosis. Follow-up results in 295 patients. Spine.

[43] MaCollough NC 3d, Schultz M, Javech N, Latta L (1981). Miami TLSO in the management of scoliosis: preliminary results in 100 cases. J Pediatr Orthop.

[44] Laurnen EL, Tupper JW, Mullen MP (1983). The Boston brace in thoracic scoliosis. A preliminary report. Spine.

[45] Jonasson-Rajala E, Josefsson E, Lundberg B, Nilsson H (1984). Boston thoracic brace in the treatment of idiopathic scoliosis. Initial correction. Clin Orthop.

[46] Périé D, Aubin CE, Petit Y, Beauséjour M, Dansereau J, Labelle H (2003). Boston brace correction in idiopathic scoliosis: a biomechanical study. Spine.

[47] Wynarsky GT, Schultz AB (1991). Optimization of skeletal configuration. Studies of scoliosis correction biomechanics. J Biomech.

[48] Aubin CA, Dansereau J, Labelle H (1993). Biomechanical simulation of the effect of the Boston brace on a model of the scoliotic spine and thorax. Ann Chir.

[49] Aubin CE, Dansereau J, De Guise JA, Labelle H (1997). Rib cage-spine coupling patterns involved in brace treatment of adolescent idiopathic scoliosis. Spine.

[50] Hopf C, Heine J (1985). Long-term results of conservative treatment of scoliosis using Chêneau brace. Z Orthop Ihre Grenzeb.

[51] Weiss HR, Deez-Kraus K. Quality criteria of scoliosis bracing assessment of primary correction. Proceedings book of the 20^th^ Annual Meeting of the British Scoliosis Society, Low Wood Hotel Windermere 23th-24^th^ of March 1995;29.

[52] Rigo M (1995). The Chêneau brace. Preliminary results. Résonances Eur Rachis.

[53] Liljenqvist U (1998). Conservative treatment of idiopathic scoliosis with the Chêneau brace.

[54] Rigo M, Quera-Salvá G, Puigdevall N, Martínez M (2002). Retrospective results in immature idiopathic scoliosis patients treated with a Chêneau brace. Stud Health Technol Inform.

[55] Weiss HR, Rigo M (2011). Expert-driven Chêneau applications: description and in-brace corrections. Physiother Theory Pract.

[56] Maruyama T (2012). Early results of Rigo-Chêneau type brace treatment. Scoliosis.

[57] Rigo M, Gallo D, Dallmayer R (2010). In-brace correction of the Cobb angle with RSC-CAD CAM compared with ‘hand made’ from the original author. Scoliosis.

[58] Gallo D, Wood G, Dallmayer R (2011). Quality control of idiopathic scoliosis treatment in 147 patients while using the RSC brace. J Prosthet Orthot.

[59] Rigo M, Gallo D (2009). A new brace design to treat single long thoracic scoliosis. Comparison of the in-brace correction in two groups treated with the new and the classical models. Scoliosis.

[60] Udén A, Willner S (1983). The effect of lumbar flexion and Boston Thoracic brace on the curves in idiopathic scoliosis. Spine.

[61] Willner S (1984). Effect of the Boston thoracic brace on the frontal and sagittal curves of the spine. Acta Orthop Scand.

[62] Labelle H, Dansereau J, Bellefleur C, Poitras B (1992). 3-D study of the immediate effect of the Boston brace on the scoliotic lumbar spine. Ann Chir.

[63] Cahuzac JP, Sales de Gauzy J, Kany J. Traitement de 161 scolioses idiopatique par le corset de C.T.M. In: Proceedings book od the 2^nd^ Congreso Transpirenaico de Rehabilitación. Ed: ICS. Ciutat Sanitària I Universitaria de Bellvitge. L’Hospitalet, Barcelona 1993:55-56.

[64] Périé D, De Gauzy S, Sévely A, Hobatho MC (2001). In vivo geometrical evaluation of Cheneau-Toulouse_Munster brace effect on scoliotic spine using MRI method. Clin Biomech.

[65] Schmitz A, Kandyba J, Jaeger U, Koenig R, Schmitt O (2002). Brace effect in scoliosis in the sagittal plane – an MRI study. Z Orthop Ihre Grenzgeb.

[66] Schmitz A, König R, Kandyba J, Pennekamp P, Schmitt O, Jaeger UE (2005). Visualization of the brace effect on the spinal profile in idiopathic scoliosis. Eur Spine J.

[67] Willers U, Normelli H, Aaro S, Svenson O, Hedlund R (1993). Long-term results of Boston brace treatment on vertebral rotation in idiopathic scoliosis. Spine.

[68] Rigo M (1999). 3D correction of trunk deformity in patients with idiopathic scoliosis using Chêneau brace. Stud Health Technol Inform.

[69] Lebel DE, Al-Aubaidi Z, Shin EJ, Howard A, Zeller R (2013). Three dimensional analysis of brace biomechanical efficacy for patients with AIS. Eur Spine J.

[70] Weiss HR, Weiss G, Schaar HJ (2003). Incidence of surgery in conservatively treated patients with idiopathic scoliosis. Pediatr Rehabil.

[71] Rigo M, Reiter CH, Weiss HR (2003). Effect of conservative management on the prevalence of surgery in patients with adolescent idiopathic scoliosis. Pediatr Rehabil.

[72] Weiss HR, Weiss G (2005). Brace treatment during pubertal growth spurt in girls with idiopathic scoliosis (IS) – A prospective trial comparing two different concepts. Pediatr Rehabil.

[73] Cinnella P, Muratone M, Testa E, Bondente PG (2009). The treatment of adolescent idiopathic scoliosis with Chêneau brace: long-term outcome. Scoliosis.

[74] Zaborowska-Sapeta K, Kowalski IM, Kotwicki T, Protasiewicz-Faldowska H, Kiebzak W (2011). Effectiveness of Chêneau brace treatment for idiopathic scoliosis: Prospective sturdy in 79 patients followed to skeletal maturity. Scoliosis.

[75] Ovadia D, Eylon S, Mashiah A, Wientroub S, Lebel ED (2012). Factors associated with the success of the Rigo System Chêneau brace in treating mild to moderate adolescent idiopathic scoliosis. J Child Orthop.

[76] Rigo M (2003). Radiological and cosmetic improvement 2 years after brace weaning – a case report. Pediatr Rehabil.

[77] Grivas TB (2014). Rib Index. Scoliosis.

[78] Rivett L, Stewart A, Potterton J (2014). The effect of compliance to a Rigo System Cheneau brace and specific exercise programme on idiopathic scoliosis curvature: a comparative study: SOSORT 2014 award winner. Scoliosis.

[79] Perdriolle R, Vidal J (1985). Thoracic idiopathic scoliosis curve evolution and prognosis. Spine.

